# Supplementary material for: Anion-Binding Properties of Short Linear Homopeptides
Source: Int J Mol Sci. 2024 May 11;25(10):5235. doi: 10.3390/ijms25105235 (PMC11121566; doi:10.3390/ijms25105235)
Supplement: Supplementary file 1 [file ijms-25-05235-s001.zip › ijms-3003235-supplementary.pdf]

Supporting Information

Anion-Binding Properties of Short Linear Homopeptides

Matija Modrušan,[a] Lucija Glazer,[a] Lucija Otmačić,[a] Ivo Crnolatac,[b] Nikola Cindro,[a] Nikolina Vidović,[c] Ivo Piantanida,[b] Giovanna Speranza,[d] Gordan Horvat[a,*] and Vladislav Tomišić[a]

[a] Department of Chemistry, Faculty of Science, Horvatovac 102a, Zagreb, Croatia

[b] Department of Organic Chemistry and Biochemistry, Ruđer Bošković Institute, Bijenička cesta 54, Zagreb, Croatia

[c] Faculty of Biotechnology and Drug Development, University of Rijeka, Radmile Matejčić 2, 51000 Rijeka, Croatia

[d] Department of Chemistry, University of Milan, Via Golgi 19, Italy

*ghorvat@chem.pmf.hr

Synthesis of compound L3

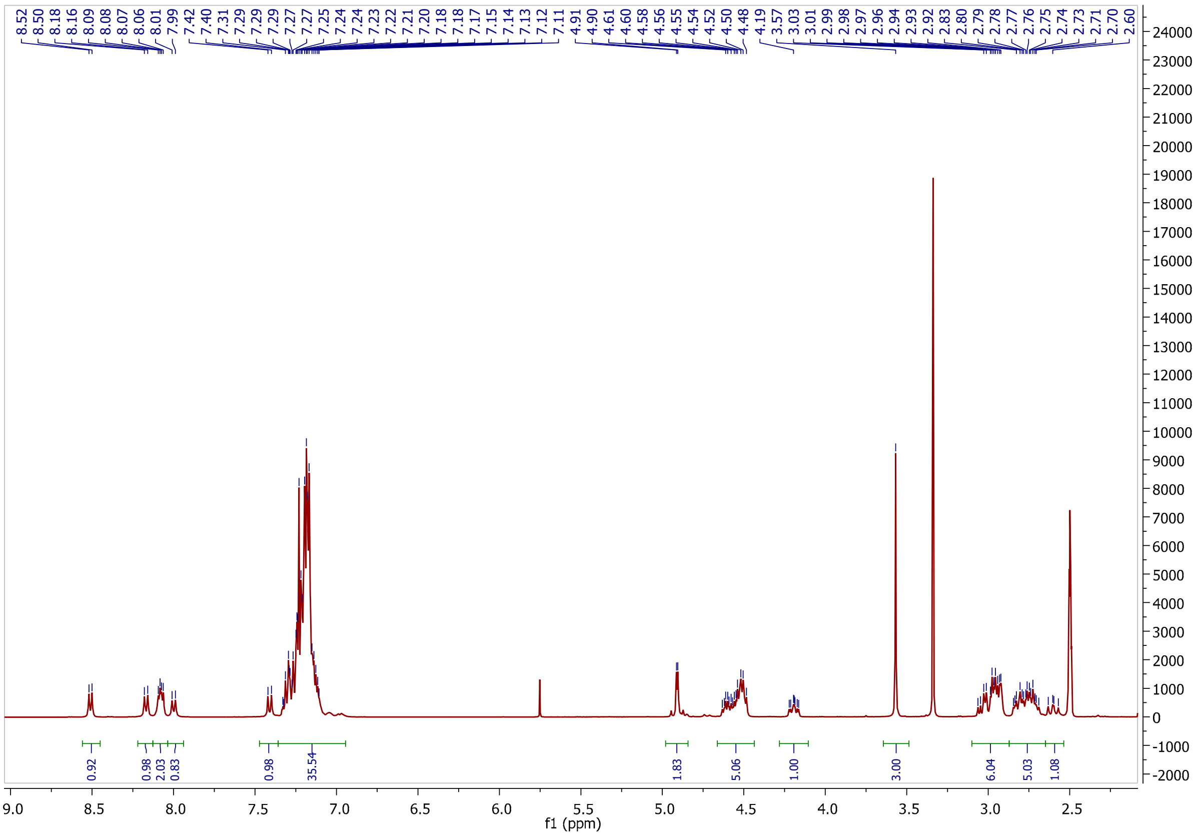

Figure S1. ^{1}H NMR spectrum of compound **1**.

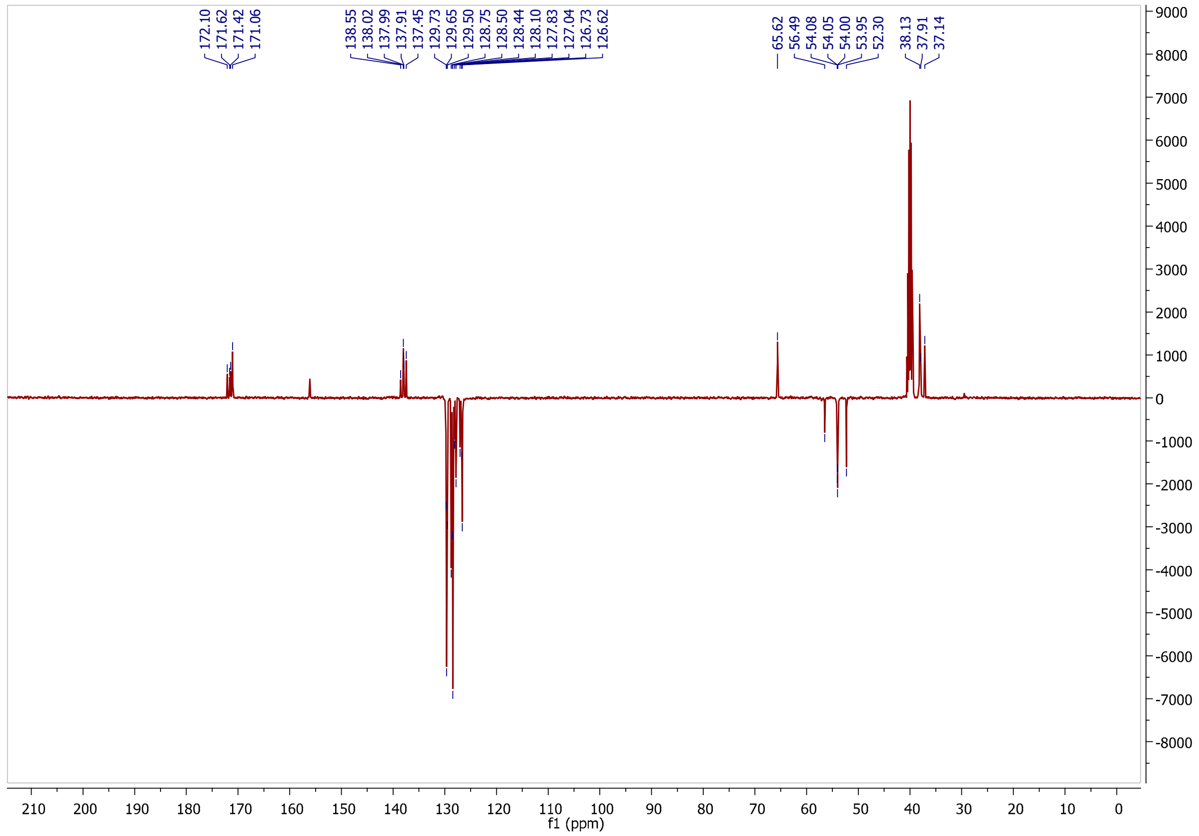

Figure S2. ^{13}C NMR spectrum of compound **1**.

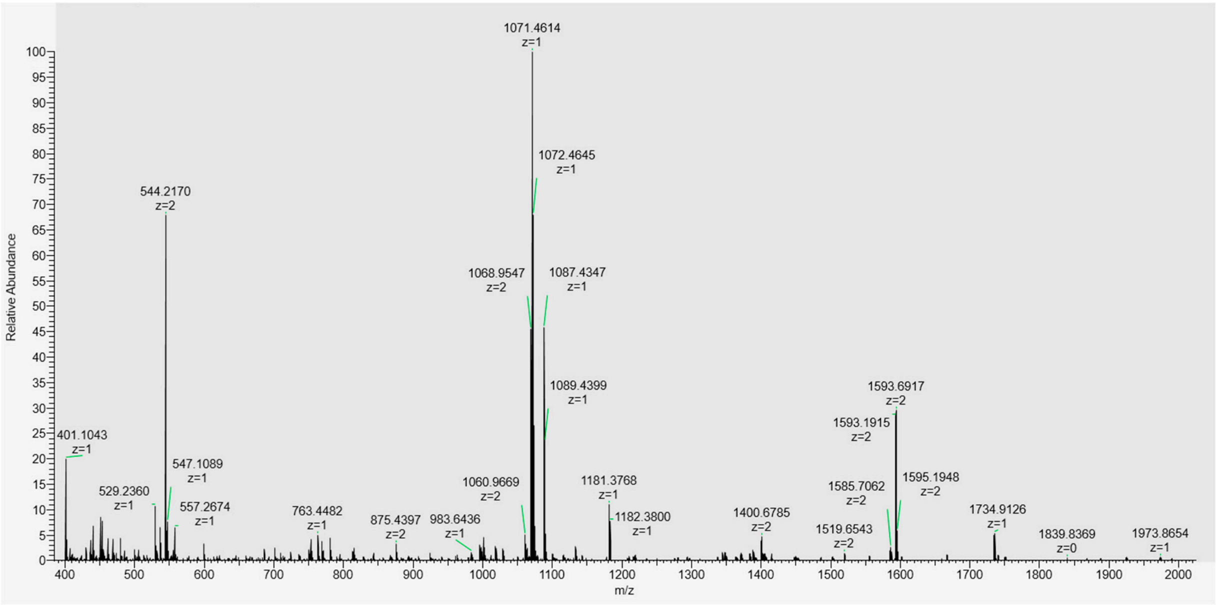

Figure S3. HRMS (ESI^+) spectrum of compound **1**.

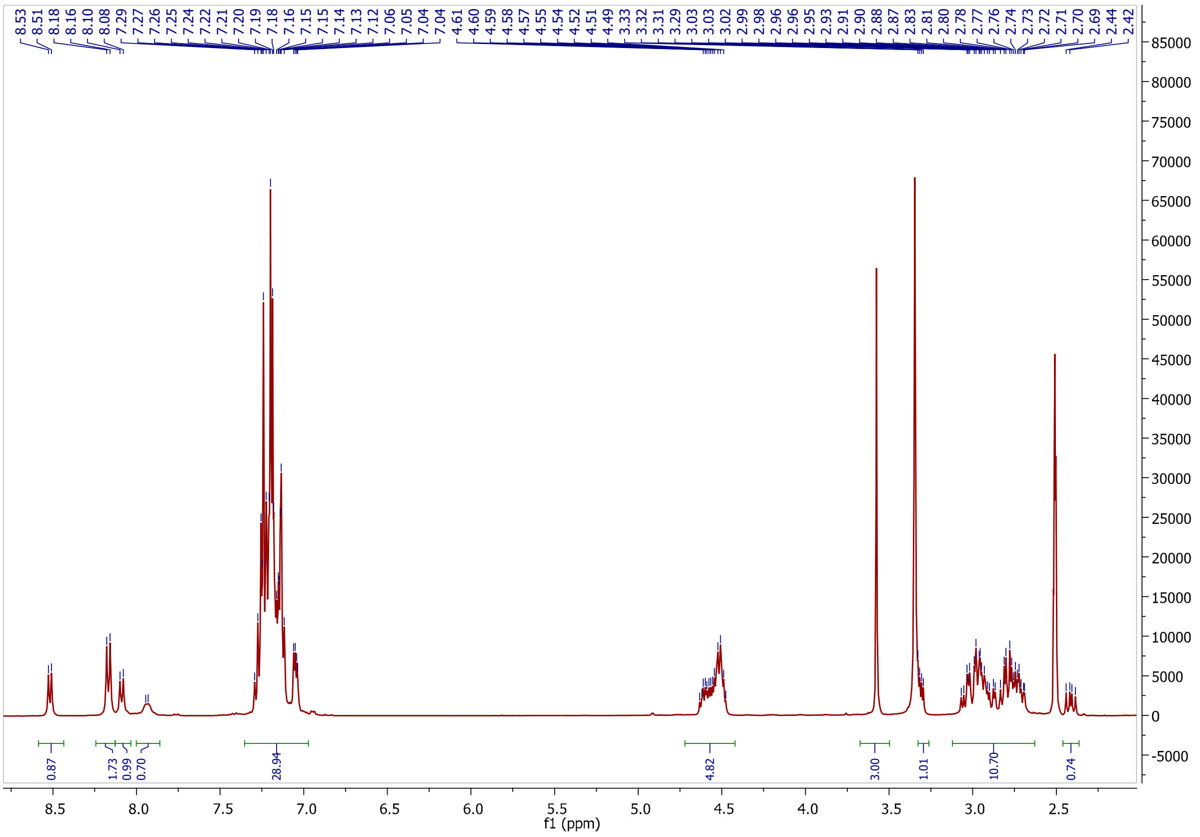

Figure S4. ^{1}H NMR spectrum of compound **L3**.

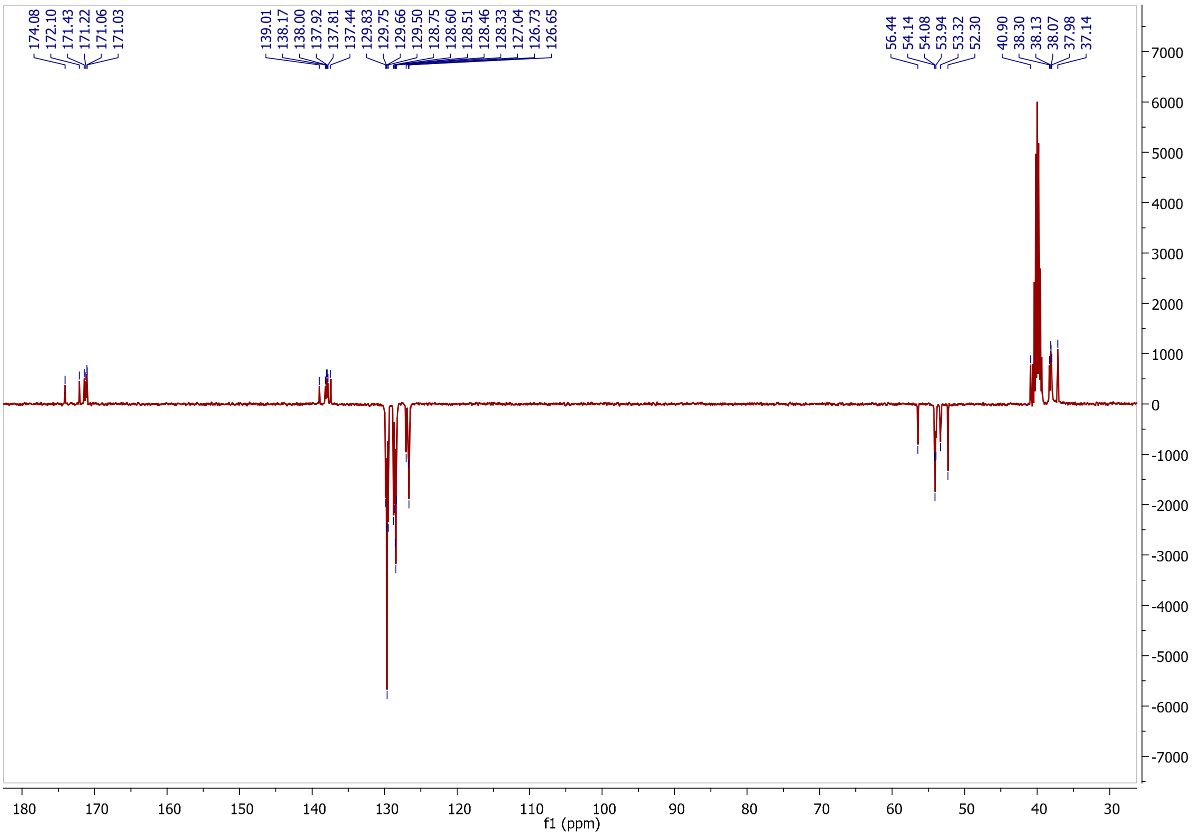

Figure S5. ^{13}C NMR spectrum of compound **L3**.

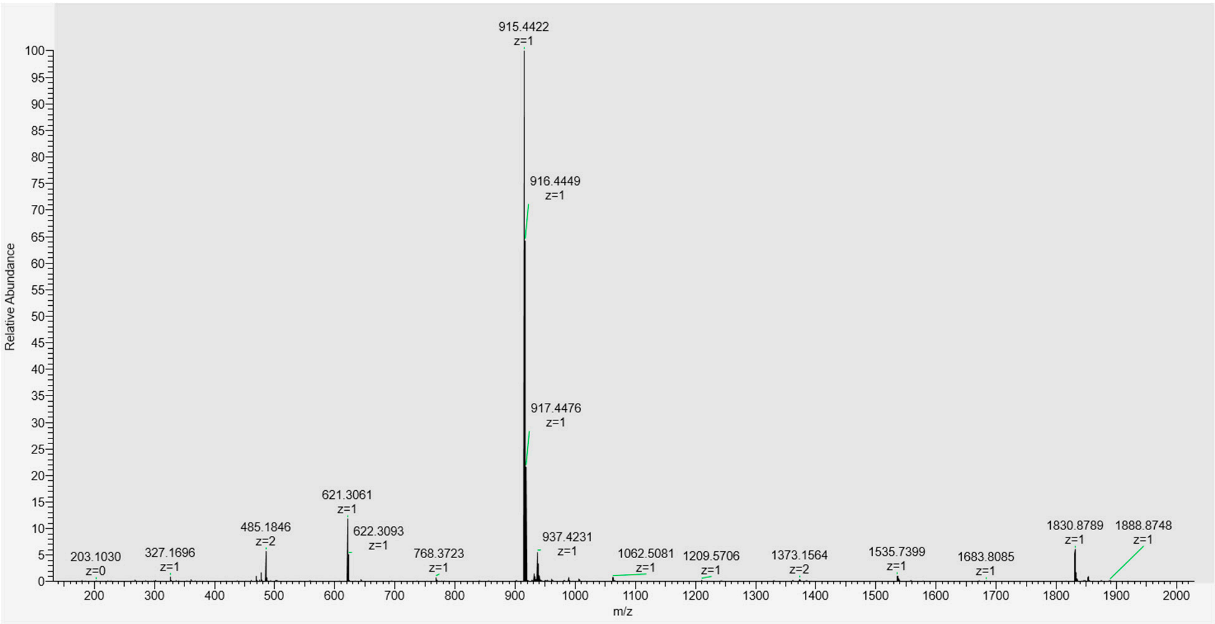

Figure S6. HRMS (ESI^{+}) spectrum of compound **L3**.

Anion complexation by peptides in MeCN

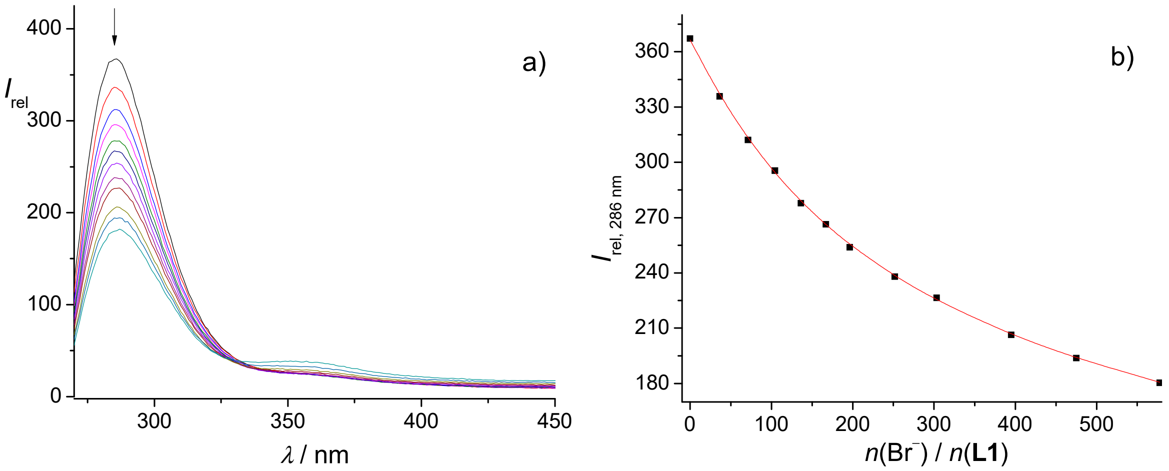

Figure S7. a) Spectrofluorimetric titration of **L1** ($c = 8.80 \times 10^{-5}$ mol dm^{-3}) with TBABr ($c = 0.14$ mol dm^{-3}) in acetonitrile at 25.0 °C; V_0(**L1**) = 2.20 cm^3; $\lambda_{ex} = 260$ nm; excitation slit 5 nm, emission slit 10 nm. Spectra are corrected for dilution. b) Relative fluorescence intensity at 286 nm as a function of anion to peptide molar ratio. ■ experimental; – calculated.

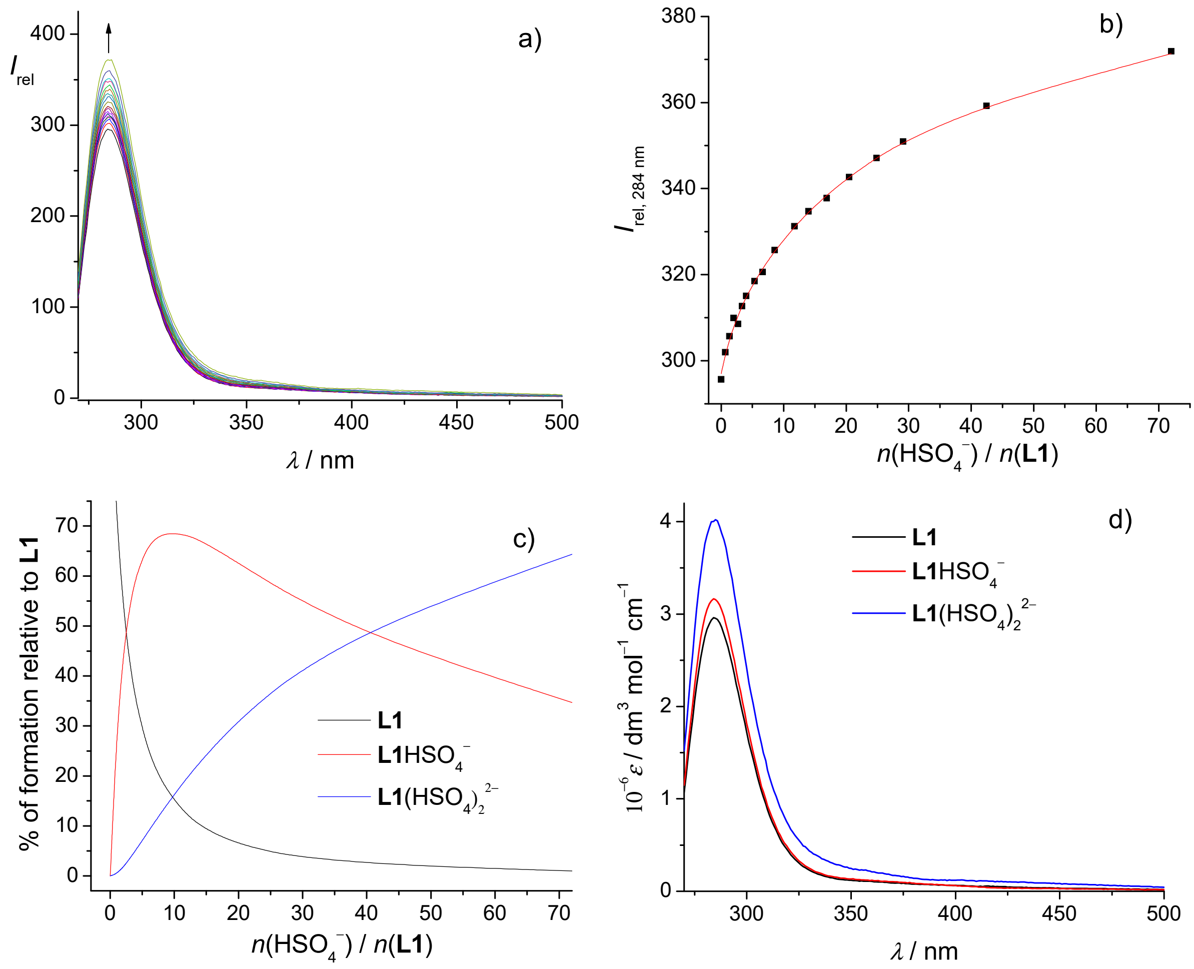

Figure S8. a) Spectrofluorimetric titration of **L1** ($c = 1.00 \times 10^{-4}$ mol dm^{-3}) with **L1** containing solution of TBAHSO$_4$ ($c = 0.030$ mol dm^{-3}) in acetonitrile at 25.0 °C; V_0(**L1**) = 2.20 cm^3; λ_{ex} = 260 nm; excitation slit 5 nm, emission slit 10 nm. Spectra are corrected for dilution. b) Relative fluorescence intensity at 284 nm as a function of anion to peptide molar ratio. ■ experimental; – calculated. c) Distribution of complex species during the titration of peptide **L1** with TBAHSO$_4$. d) Molar spectra of free peptide **L1** and its complexes with HSO_4^- anion.

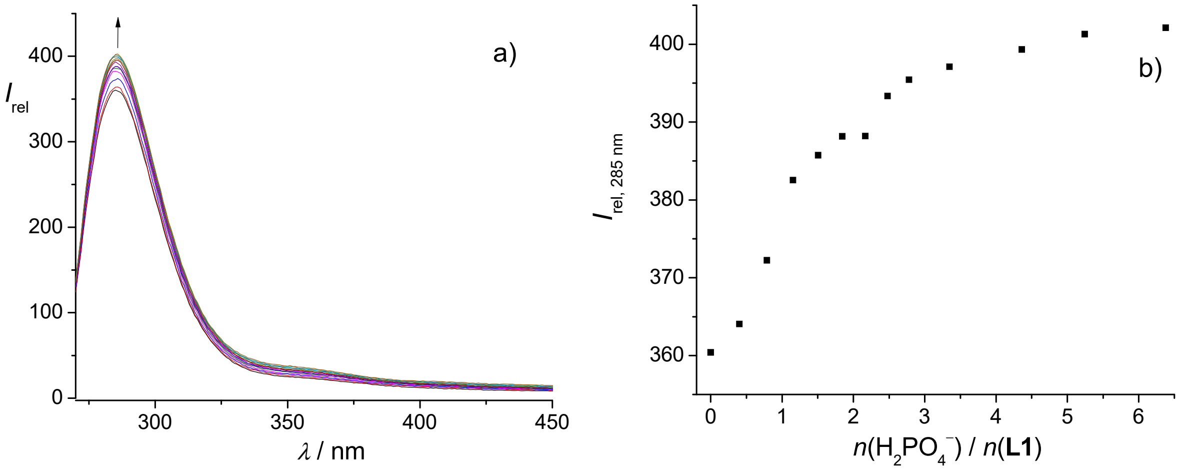

Figure S9. a) Spectrofluorimetric titration of **L1** ($c = 8.80 \times 10^{-5}$ mol dm^{-3}) with TBAH$_2$PO$_4$ ($c = 1.59 \times 10^{-3}$ mol dm^{-3}) in acetonitrile at 25.0 °C; V_0(**L1**) = 2.20 cm^3; λ_{ex} = 260 nm; excitation slit 5 nm, emission slit 10 nm. Spectra are corrected for dilution. b) Relative fluorescence intensity at 285 nm as a function of anion to peptide molar ratio. ■ experimental.

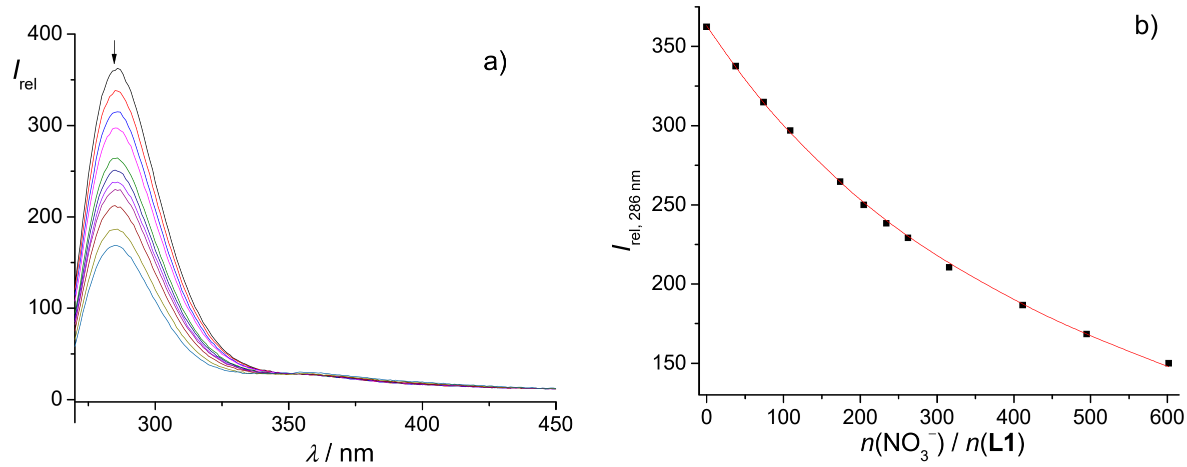

Figure S10. a) Spectrofluorimetric titration of **L1** ($c = 8.80 \times 10^{-5}$ mol dm^{-3}) with TBANO$_3$ ($c = 0.15$ mol dm^{-3}) in acetonitrile at 25.0 °C; V_0(**L1**) = 2.20 cm^3; λ_{ex} = 260 nm; excitation slit 5 nm, emission slit 10 nm. Spectra are corrected for dilution. b) Relative fluorescence intensity at 286 nm as a function of anion to peptide molar ratio. ■ experimental; – calculated.

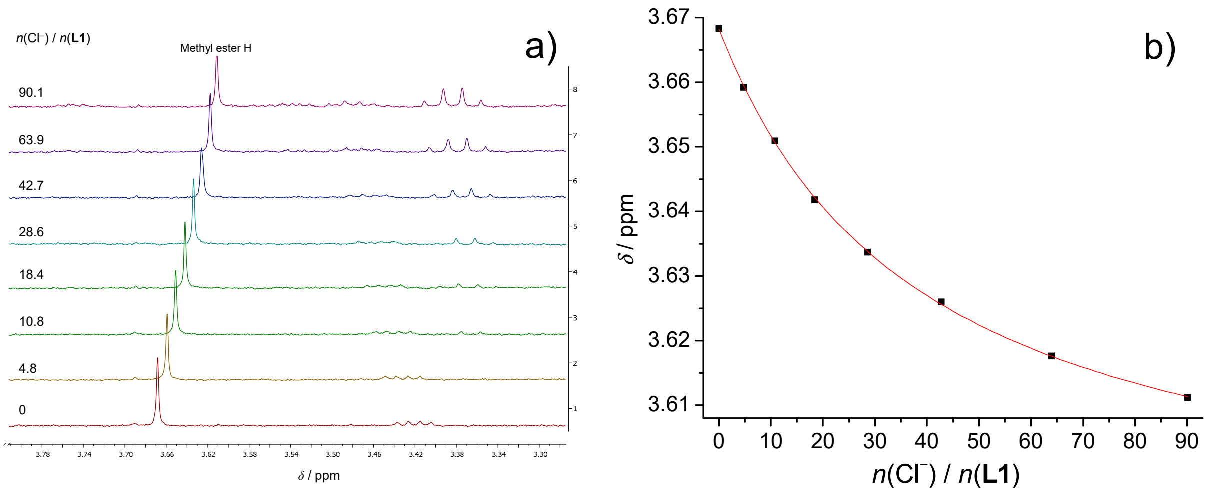

Figure S11. a) ^{1}H NMR titration of **L1** (c = 1.49 × 10^{-4} mol dm^{-3}) with TEACl (c = 0.033 mol dm^{-3}) in deuterated acetonitrile at 25.0 °C; V_0(**L1**) = 0.50 cm^3. b) Chemical shift of methyl ester protons at 3.67 ppm as a function of anion to peptide molar ratio. ■ experimental; – calculated.

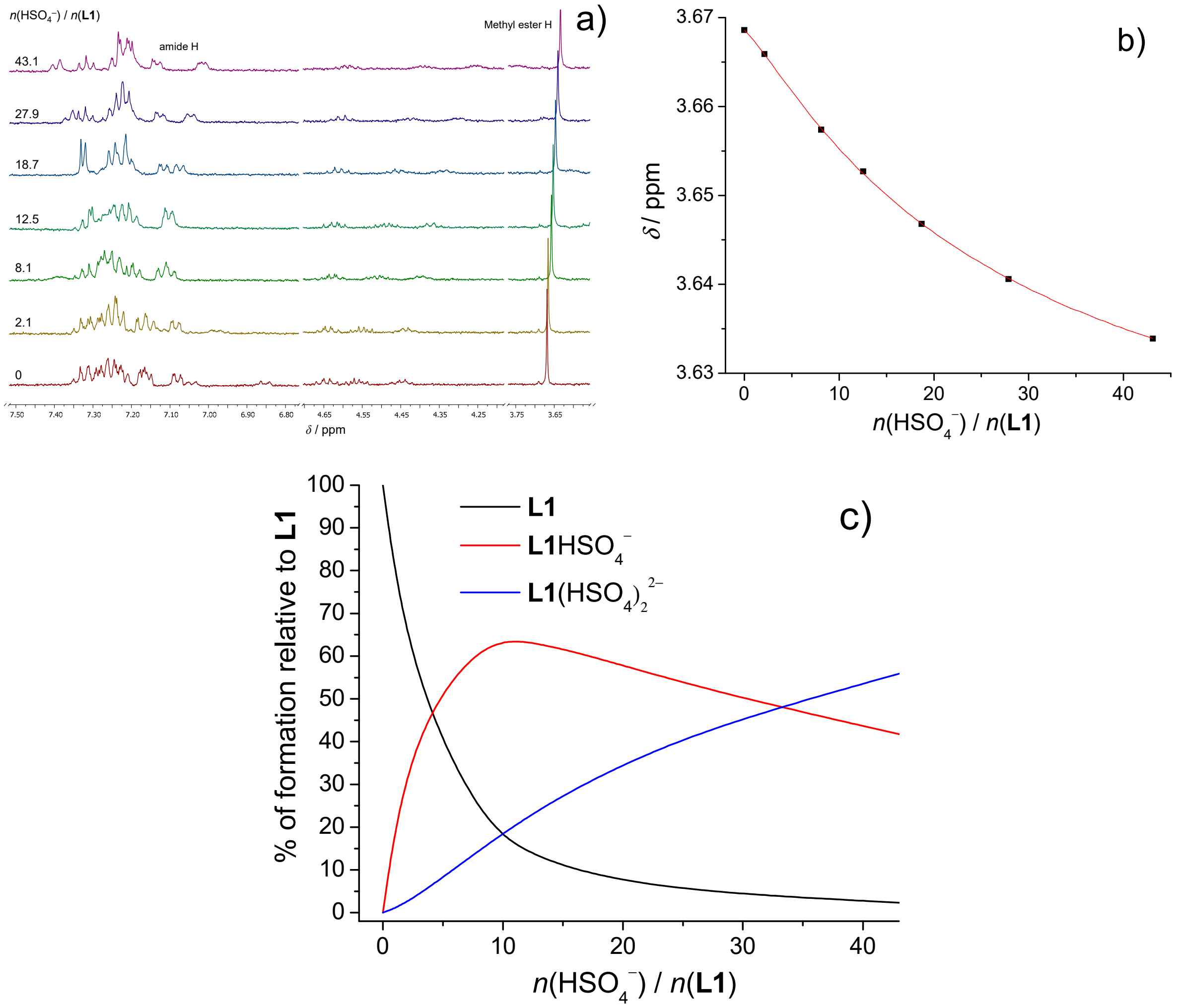

Figure S12. a) ^{1}H NMR titration of **L1** ($c = 1.73 \times 10^{-4}$ mol dm^{-3}) with TBAHSO$_4$ ($c = 0.021$ mol dm^{-3}) in deuterated acetonitrile at 25.0 °C; V_0(**L1**) = 0.50 cm^3. b) Chemical shift of methyl ester protons at 3.67 ppm as a function of anion to peptide molar ratio. ■ experimental; – calculated. c) Distribution of complex species during the titration of peptide **L1** with TBAHSO$_4$.

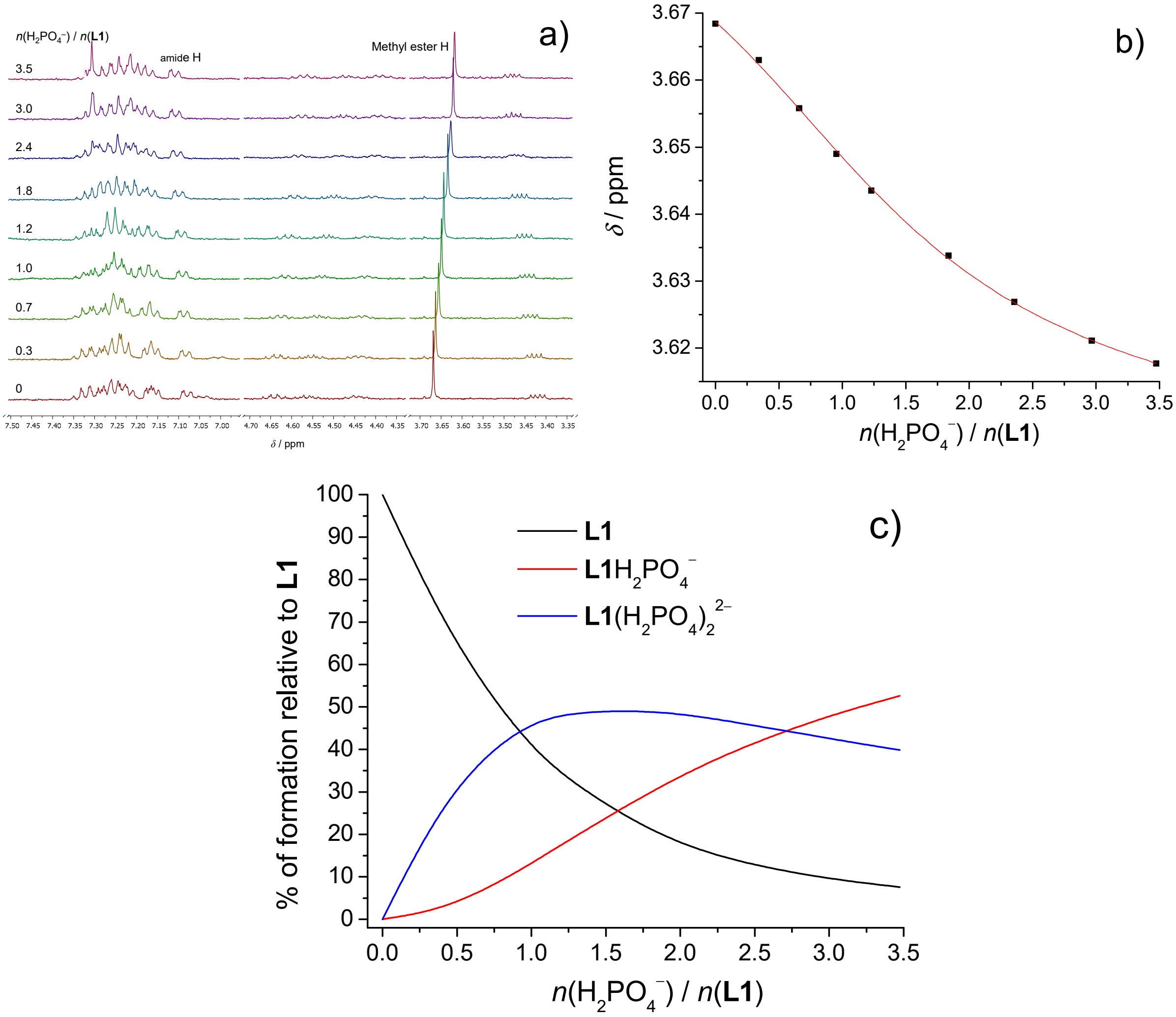

Figure S13. a) ^{1}H NMR titration of **L1** ($c = 1.73 \times 10^{-4}$ mol dm^{-3}) with TBAH$_2$PO$_4$ ($c = 1.54 \times 10^{-3}$ mol dm^{-3}) in deuterated acetonitrile at 25.0 °C; V_0(**L1**) = 0.50 cm^3. b) Chemical shift of methyl ester protons at 3.67 ppm as a function of anion to peptide molar ratio. ■ experimental; – calculated. c) Distribution of complex species during the titration of peptide **L1** with TBAH$_2$PO$_4$.

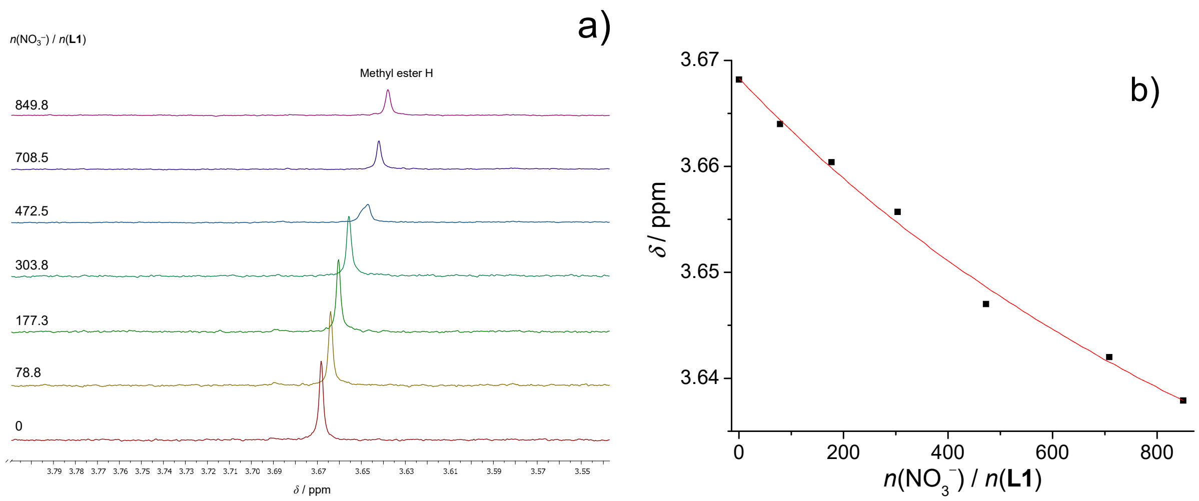

Figure S14. a) ¹H NMR titration of **L1** ($c = 1.01 \times 10^{-4}$ mol dm^{-3}) with $TBANO_3$ ($c = 0.15$ mol dm^{-3}) in deuterated acetonitrile at 25.0 °C; V_0(**L1**) = 0.50 cm^3. b) Chemical shift of methyl ester protons at 3.67 ppm as a function of anion to peptide molar ratio. ■ experimental; – calculated.

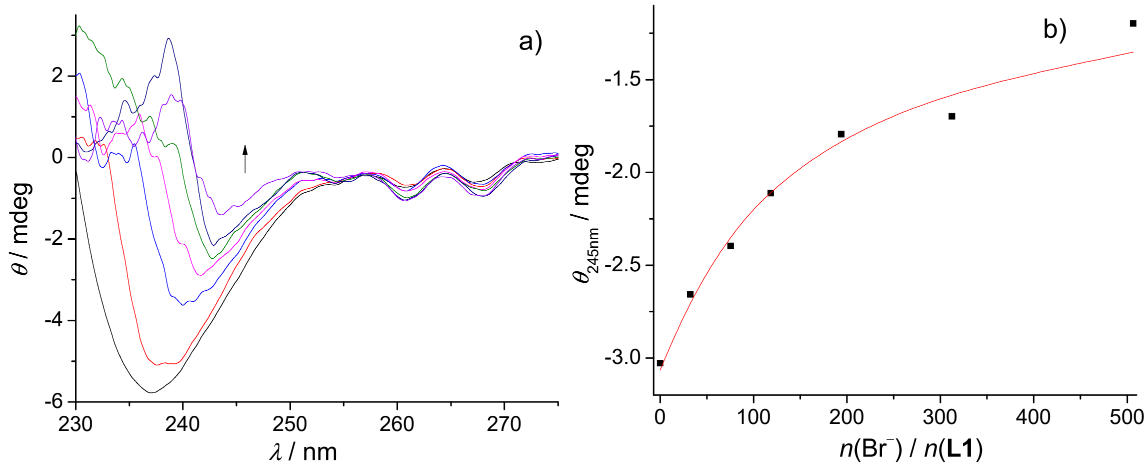

Figure S15. a) CD titration of **L1** ($c = 1.16 \times 10^{-4}$ mol dm^{-3}) with TBABr ($c = 0.25$ mol dm^{-3}) in acetonitrile at 25.0 °C; V_0(**L1**) = 2.00 cm^3; $l = 1$ cm. b) Dependence of ellipticity at 245 nm as a function of anion to peptide molar ratio. ■ experimental; – calculated.

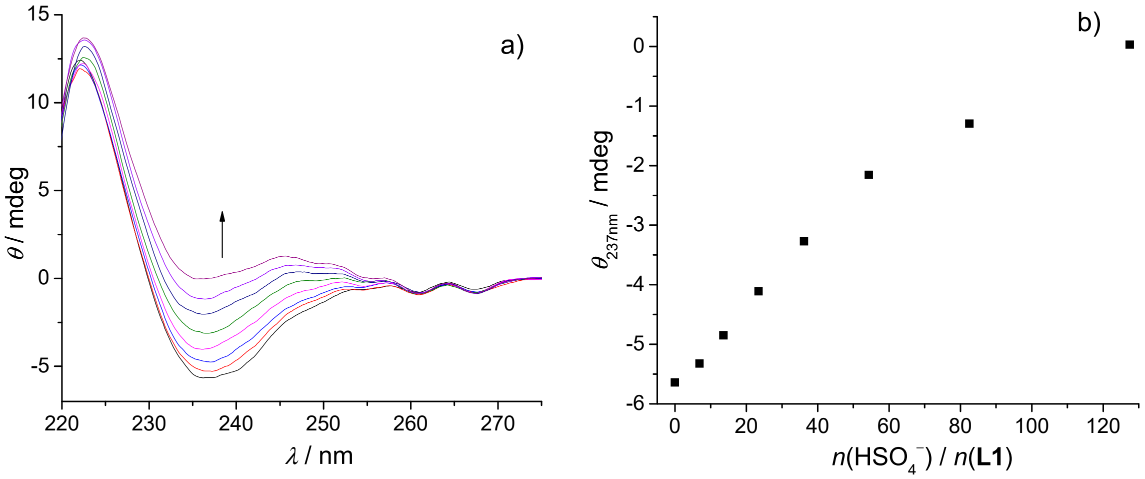

Figure S16. a) CD titration of **L1** ($c = 1.16 \times 10^{-4}$ mol dm^{-3}) with TBAHSO$_4$ ($c = 0.081$ mol dm^{-3}) in acetonitrile at 25.0 °C; V_0(**L1**) = 2.00 cm^3; l = 1 cm. b) Dependence of ellipticity at 237 nm as a function of anion to peptide molar ratio.

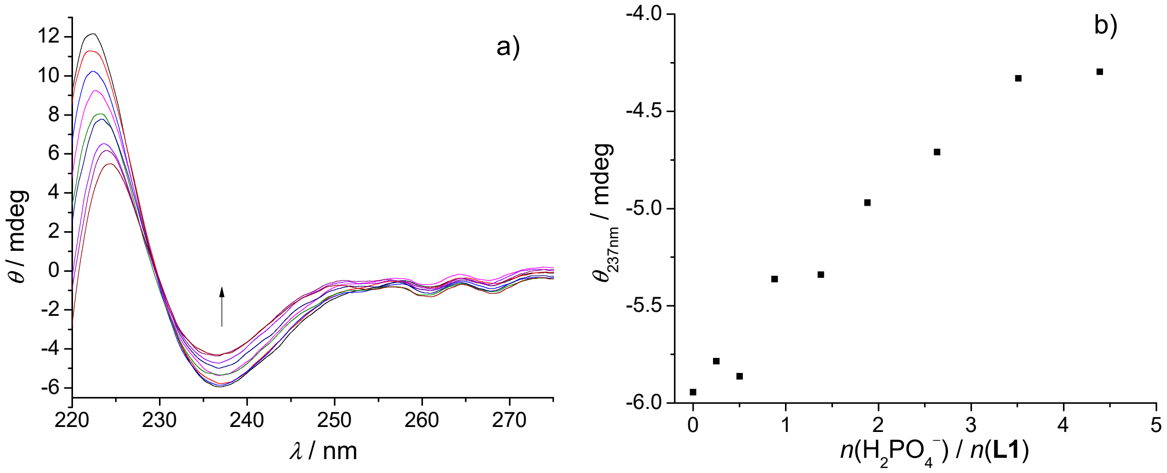

Figure S17. a) CD titration of **L1** ($c = 1.16 \times 10^{-4}$ mol dm^{-3}) with TBAH$_2$PO$_4$ ($c = 2.91 \times 10^{-3}$ mol dm^{-3}) in acetonitrile at 25.0 °C; V_0(**L1**) = 2.00 cm^3; l = 1 cm. b) Dependence of ellipticity at 237 nm as a function of anion to peptide molar ratio.

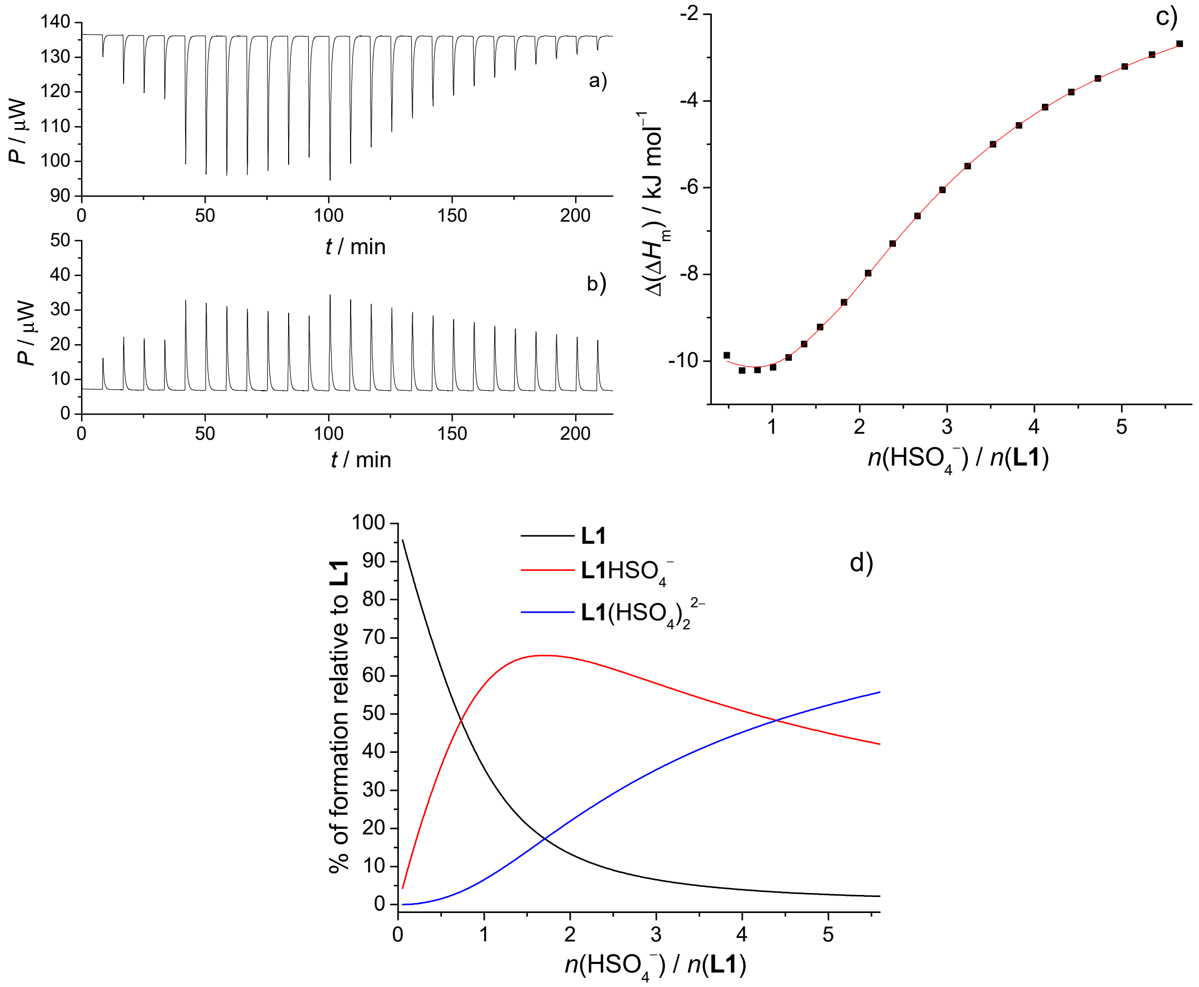

Figure S18. a) Microcalorimetric titration of **L1** ($c = 1.03 \times 10^{-3}$ mol dm^{-3}. $V = 1.4295$ cm^3) with $TBAHSO_4$ (c = 0.0249 mol dm^{-3}) in acetonitrile; ϑ = 25 °C. b) Microcalorimetric titration of acetonitrile with $TBAHSO_4$ (c = 0.0249 mol dm^{-3}); ϑ = 25 °C. c) Dependence of molar successive enthalpy change on $n(HSO_4^-)$ / n(**L1**) ratio. ■ experimental; — calculated. d) Distribution of complex species during the titration of peptide **L1** with $TBAHSO_4$.

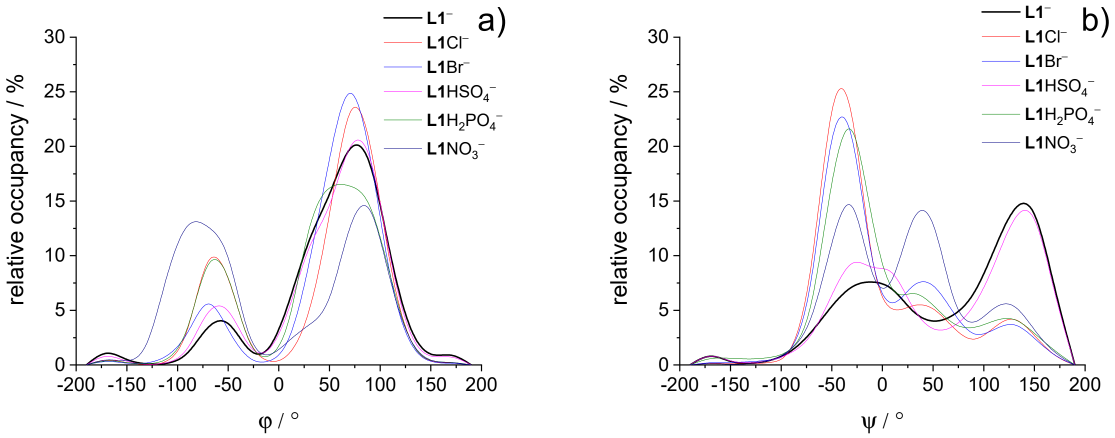

Figure S19. Distribution of a) *φ*, b) *ψ* angles of free peptide **L1** and its complexes with anions obtained by MD simulation in acetonitrile at 25 °C.

Table S1. Energies of interactions between different species (**L1**, A^-, and MeCN) obtained by MD simulations in acetonitrile at 25 °C.

	free	Cl^-	Br^-	HSO_4^-	$H_2PO_4^-$	NO_3^-
E(**L1**–A^-) / kJ mol^{-1}	–	–195	–180	–132	–222	–192
E(**L1**–MeCN) / kJ mol^{-1}	–392	–296	–296	–315	–297	–295
E(A^-–MeCN) / kJ mol^{-1}	–	–74	–74	–113	–93	–99

Table S2. Distances between nitrogen atom of N terminus and carbon atom of C terminus on free peptide **L1** and its complexes with anions obtained by MD simulations in acetonitrile at 25 °C.

	free	Cl^-	Br^-	HSO_4^-	$H_2PO_4^-$	NO_3^-
d(C–N) / Å	9.9 ± 1.4	5.2 ± 0.9	5.7 ± 1.0	7.1 ± 1.4	5.5 ± 1.0	8.5 ± 1.0

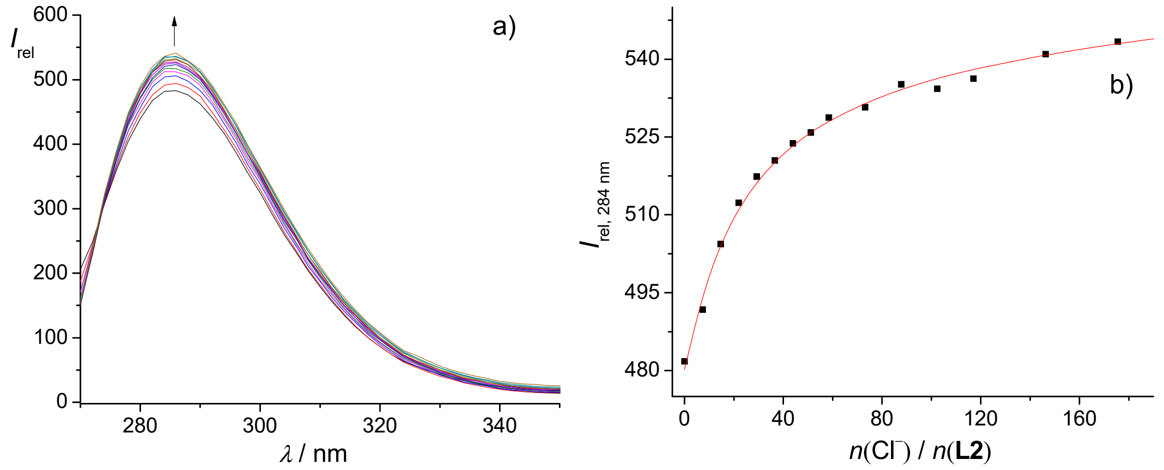

Figure S20. a) Spectrofluorimetric titration of **L2** ($c = 4.53 \times 10^{-5}$ mol dm^{-3}) with TEACl ($c = 0.029$ mol dm^{-3}) in acetonitrile at 25.0 °C; V_0(**L2**) = 2.20 cm^3; $\lambda_{ex} = 260$ nm; excitation slit 10 nm, emission slit 10 nm. Spectra are corrected for dilution. Spectra are corrected for dilution. b) Relative fluorescence intensity at 284 nm as a function of anion to peptide molar ratio. ■ experimental; – calculated.

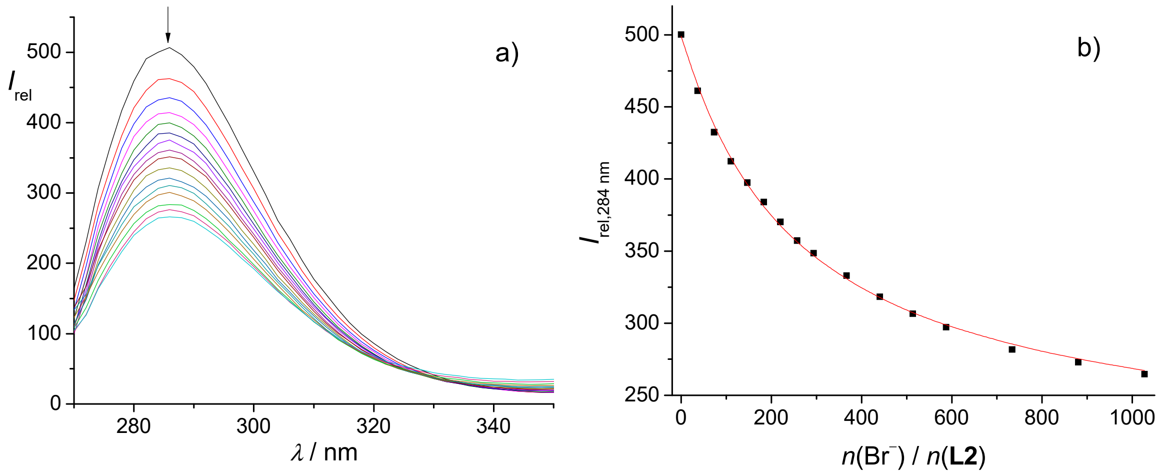

Figure S21. a) Spectrofluorimetric titration of **L2** ($c = 4.53 \times 10^{-5}$ mol dm^{-3}) with TBABr ($c = 0.15$ mol dm^{-3}) in acetonitrile at 25.0 °C; V_0(**L2**) = 2.20 cm^3; $\lambda_{ex} = 260$ nm; excitation slit 10 nm, emission slit 10 nm. Spectra are corrected for dilution. Spectra are corrected for dilution. b) Relative fluorescence intensity at 284 nm as a function of anion to peptide molar ratio. ■ experimental; – calculated.

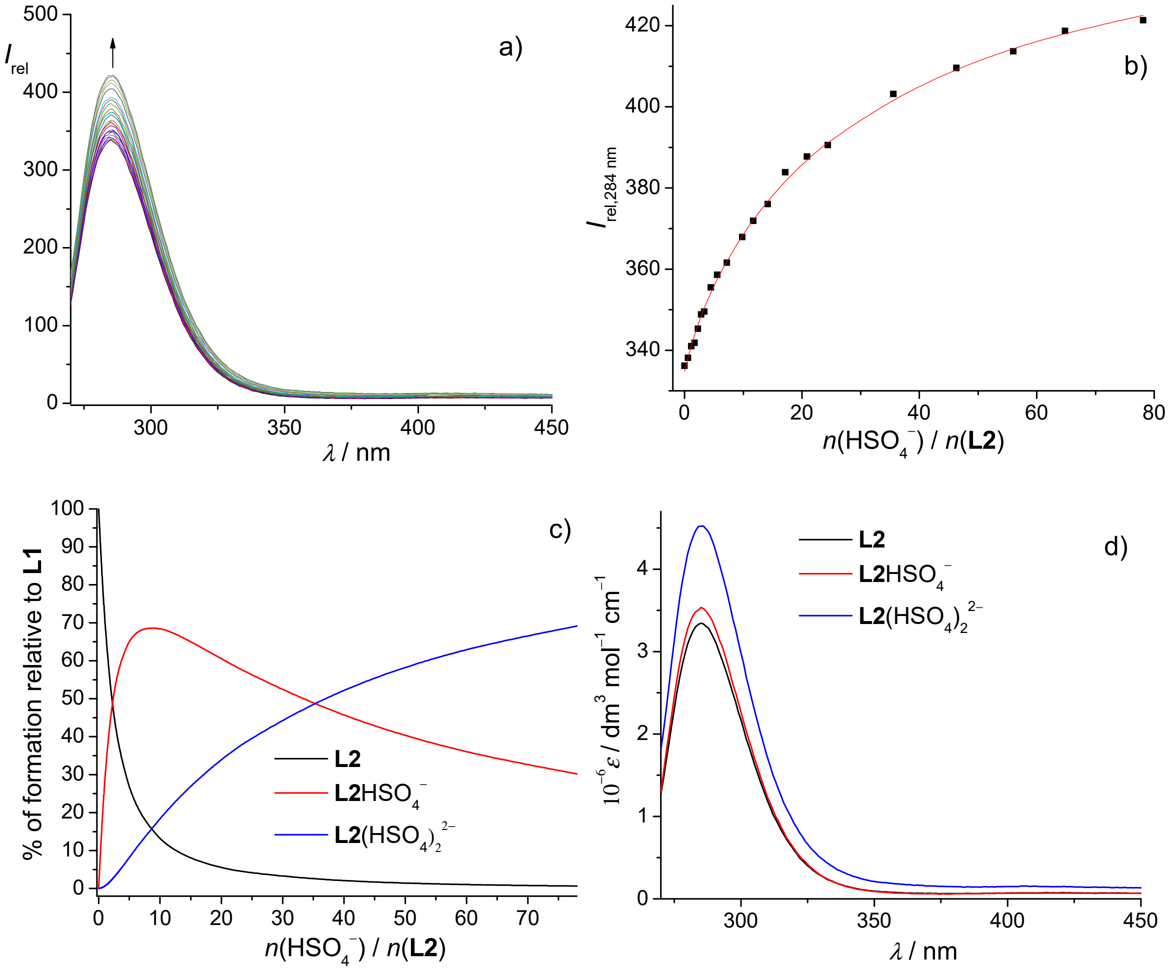

Figure S22. a) Spectrofluorimetric titration of **L2** ($c = 1.01 \times 10^{-4}$ mol dm^{-3}) with **L2** containing solution of $TBAHSO_4$ (c = 0.025 mol dm^{-3}) in acetonitrile at 25.0 °C; V_0(**L2**) = 2.20 cm^3; λ_{ex} = 260 nm; excitation slit 5 nm, emission slit 10 nm. Spectra are corrected for dilution. b) Relative fluorescence intensity at 284 nm as a function of anion to peptide molar ratio. ■ experimental; – calculated. c) Distribution of complex species during the titration of peptide **L2** with $TBAHSO_4$. d) Molar spectra of free peptide **L2** and its complexes with HSO_4^- anion.

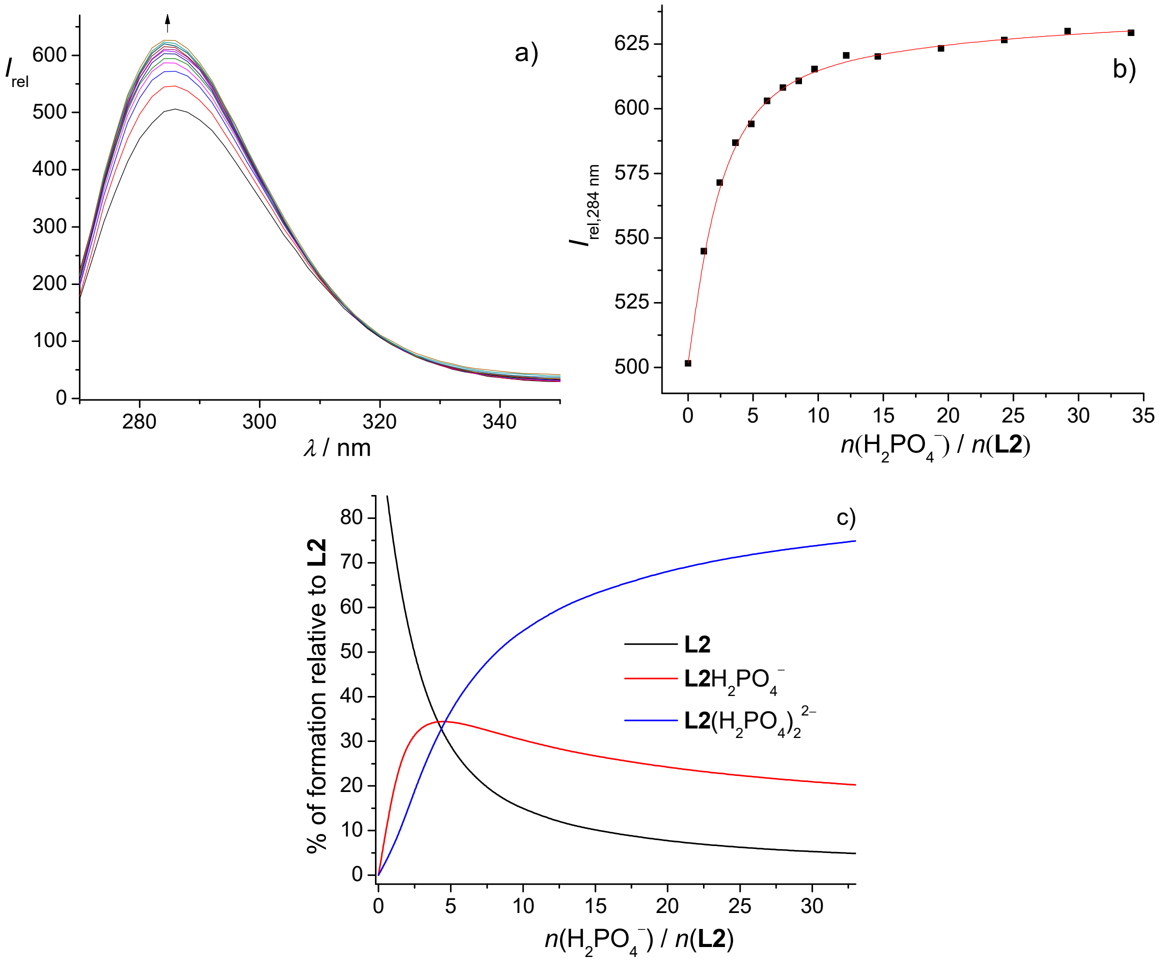

Figure S23. a) Spectrofluorimetric titration of **L2** ($c = 4.53 \times 10^{-5}$ mol dm^{-3}) with $TBAH_2PO_4$ ($c = 4.84 \times 10^{-3}$ mol dm^{-3}) in acetonitrile at 25.0 °C; V_0(**L2**) = 2.20 cm^3; λ_{ex} = 260 nm; excitation slit 10 nm, emission slit 10 nm. Spectra are corrected for dilution. Spectra are corrected for dilution. b) Relative fluorescence intensity at 284 nm as a function of anion to peptide molar ratio. ■ experimental; – calculated. c) Distribution of complex species during the titration of peptide **L2** with $TBAH_2PO_4$.

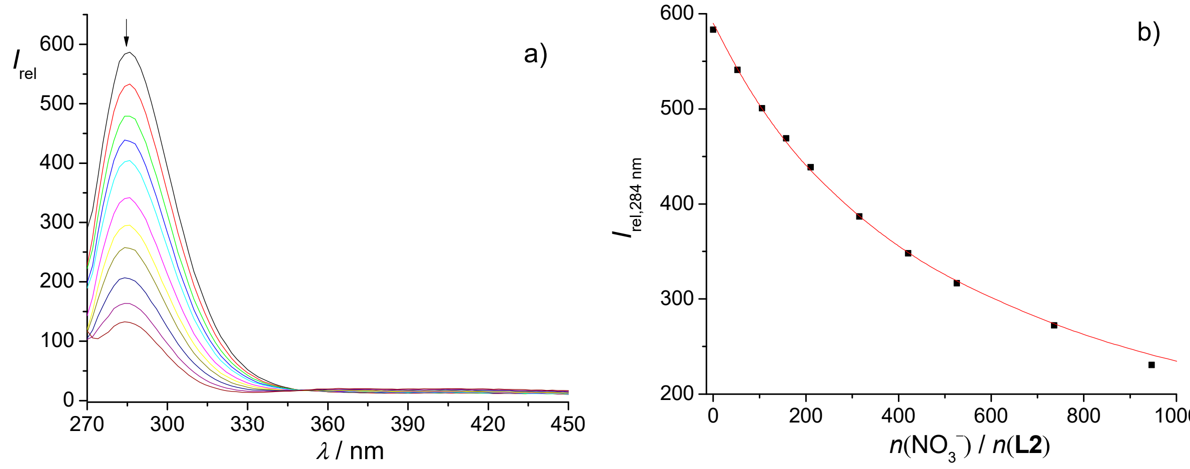

Figure S24. a) Spectrofluorimetric titration of **L2** ($c = 1.16\times\ 10^{-4}$ mol dm^{-3}) with $TBANO_3$ ($c = 0.15$ mol dm^{-3}) in acetonitrile at 25.0 °C; V_0(**L2**) = 2.20 cm^3; $\lambda_{ex} = 260$ nm; excitation slit 10 nm, emission slit 10 nm. Spectra are corrected for dilution. Spectra are corrected for dilution. b) Relative fluorescence intensity at 284 nm as a function of anion to peptide molar ratio. ■ experimental; – calculated.

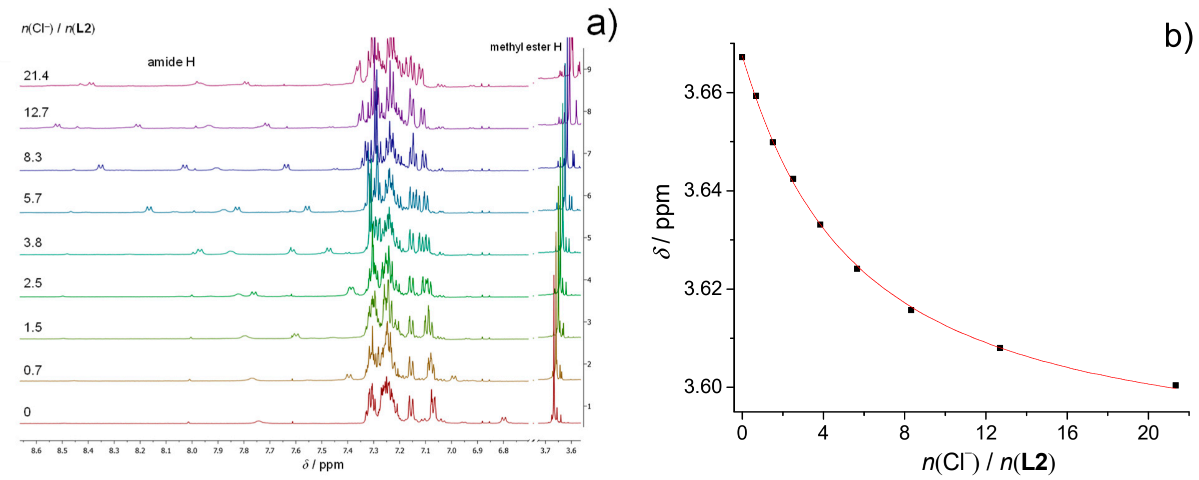

Figure S25. a) ^{1}H NMR titration of **L2** ($c = 5.01\ \times\ 10^{-4}$ mol dm^{-3}) with TEACl ($c = 0.052$ mol dm^{-3}) in deuterated acetonitrile at 25.0 °C; V_0(**L2**) = 0.50 cm^3. b) Chemical shift of methyl ester protons at 3.67 ppm as a function of anion to peptide molar ratio. ■ experimental; – calculated.

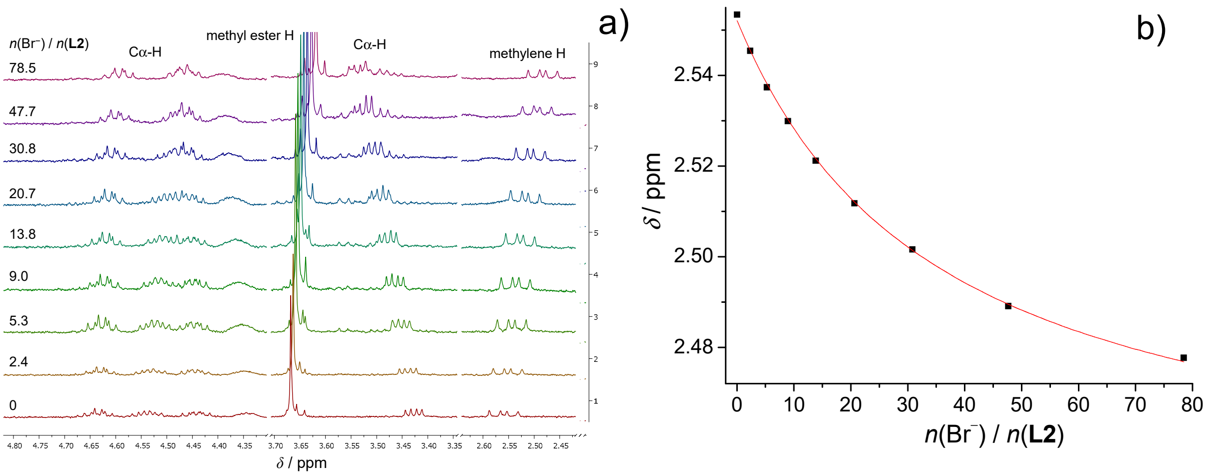

Figure S26. a) ^{1}H NMR titration of **L2** ($c = 4.95 \times 10^{-4}$ mol dm^{-3}) with TBABr ($c = 0.083$ mol dm^{-3}) in deuterated acetonitrile at 25.0 °C; V_0(**L2**) = 0.50 cm^3. b) Chemical shift of methylene protons at 2.56 ppm as a function of anion to peptide molar ratio. ■ experimental; – calculated.

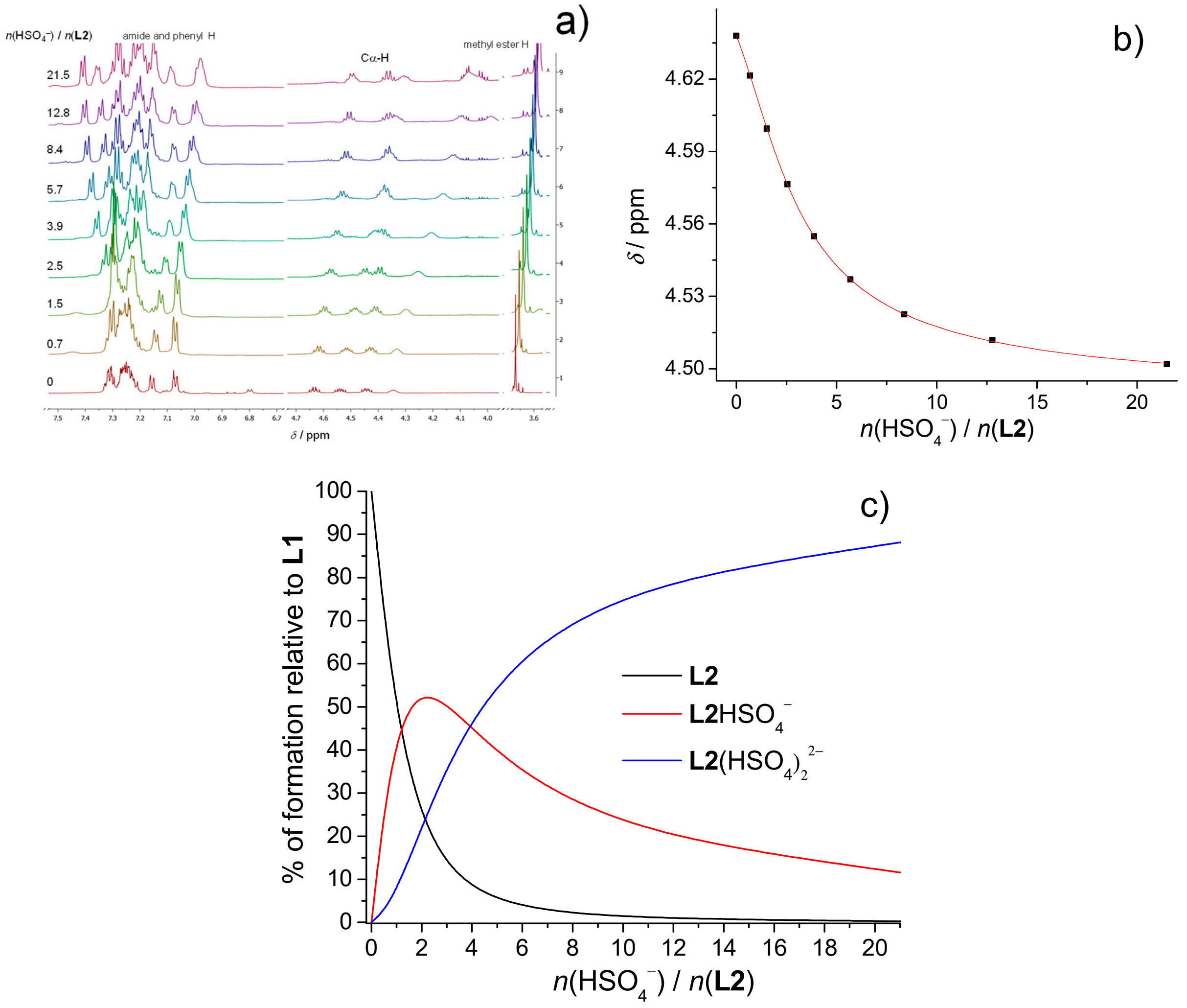

Figure S27. a) ^{1}H NMR titration of **L2** (c = 5.01 × 10^{-4} mol dm^{-3}) with TBAHSO4 (c = 0.052 mol dm^{-3}) in deuterated acetonitrile at 25.0 °C; V_0(**L2**) = 0.50 cm^3. b) Chemical shift of C–α protons at 4.64 ppm as a function of anion to peptide molar ratio. ■ experimental; – calculated. c) Distribution of complex species during the titration of peptide **L2** with TBAHSO4.

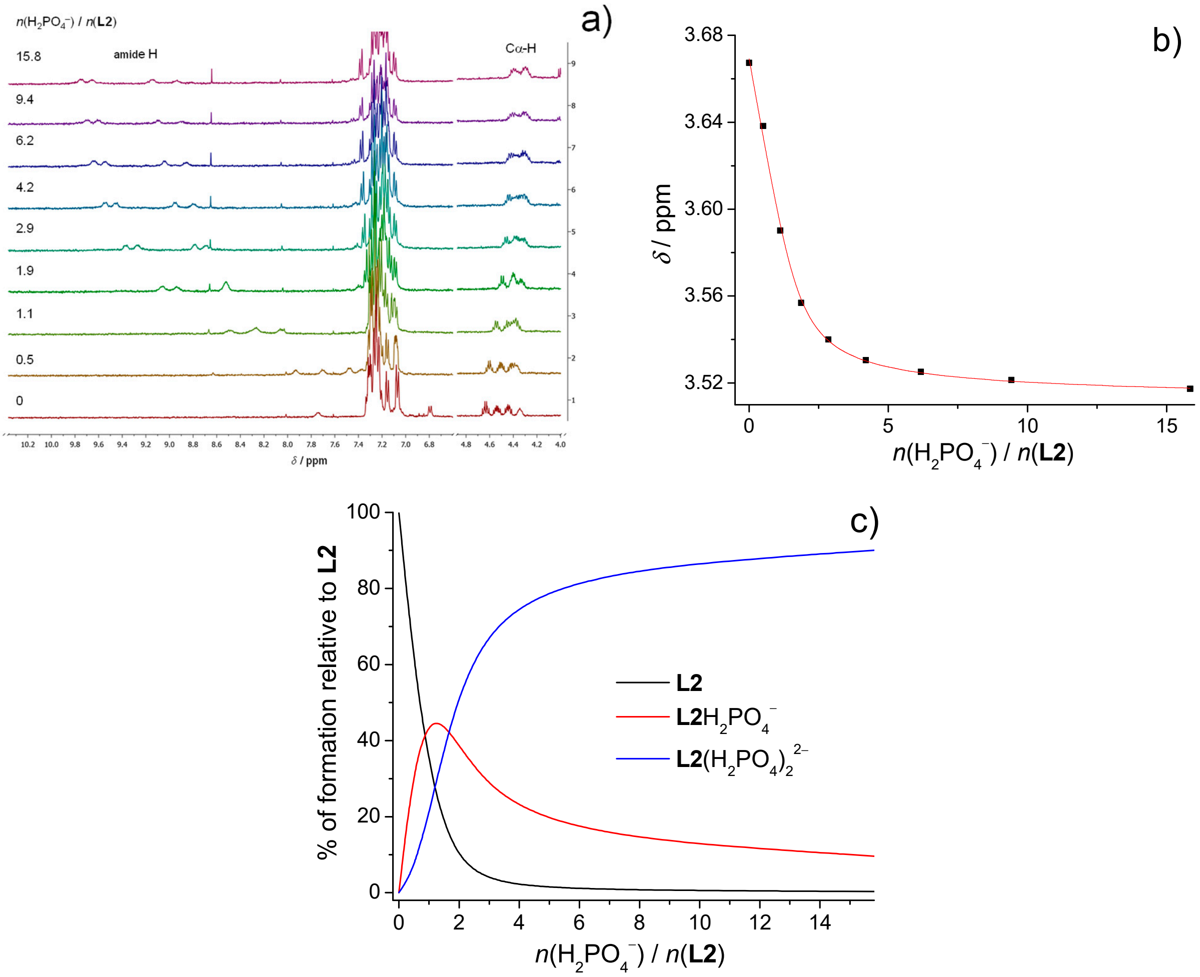

Figure S28. a) ^{1}H NMR titration of **L2** (c = 6.64 × 10^{-4} mol dm^{-3}) with TBAH$_2$PO$_4$ (c = 0.051 mol dm^{-3}) in deuterated acetonitrile at 25.0 °C; V_0(**L2**) = 0.50 cm^3. b) Chemical shift of methyl ester protons at 3.67 ppm as a function of anion to peptide molar ratio. ■ experimental; – calculated. c) Distribution of complex species during the titration of peptide **L2** with TBAH$_2$PO$_4$.

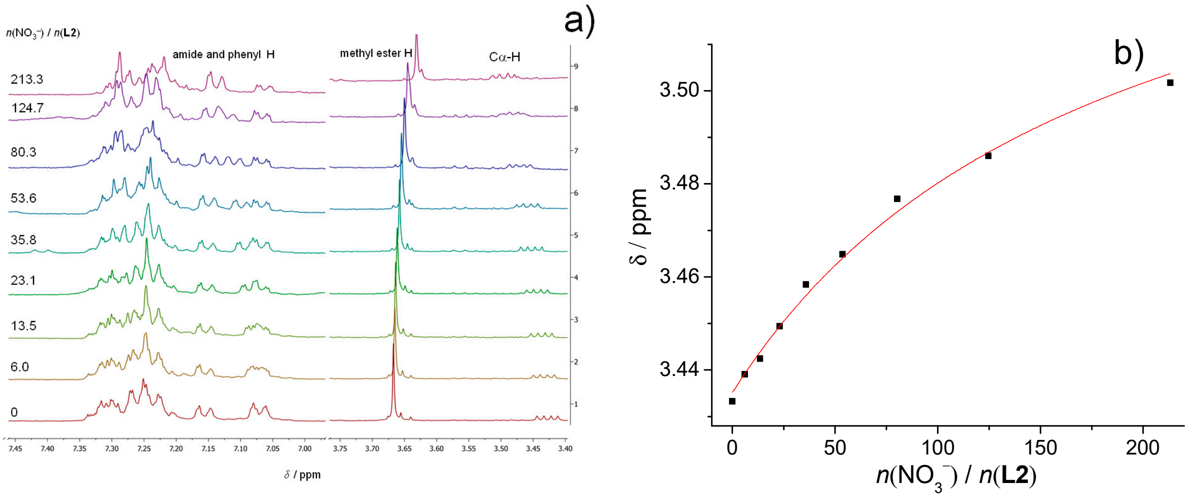

Figure S29. a) ^{1}H NMR titration of **L2** (c = 5.21 × 10^{-4} mol dm^{-3}) with TBANO$_3$ (c = 0.18 mol dm^{-3}) in deuterated acetonitrile at 25.0 °C; V_0(**L2**) = 0.50 cm^3. b) Chemical shift of C–α protons at 3.43 ppm as a function of anion to peptide molar ratio. ■ experimental; – calculated.

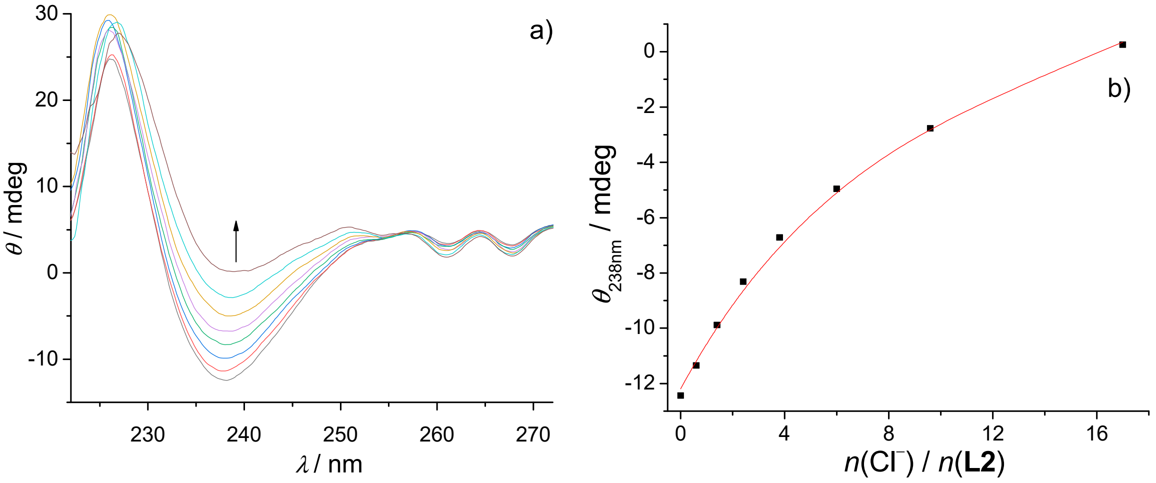

Figure S30. a) CD titration of **L2** (c = 5.00 × 10^{-4} mol dm^{-3}) with TEACl (c = 0.10 mol dm^{-3}) in acetonitrile at 25.0 °C; V_0(**L2**) = 2.00 cm^3; l = 1 cm. b) Dependence of ellipticity at 238 nm as a function of anion to peptide molar ratio. ■ experimental; – calculated.

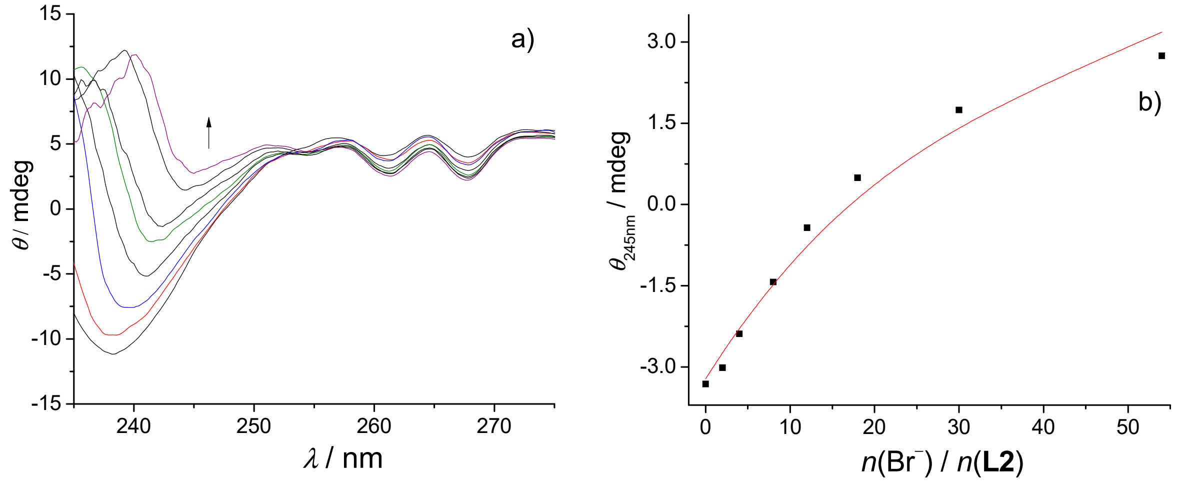

Figure S31. a) CD titration of **L2** (c = 5.00 × 10^{-4} mol dm^{-3}) with TBABr (c = 0.20 mol dm^{-3}) in acetonitrile at 25.0 °C; V_0(**L2**) = 2.00 cm^3; l = 1 cm. b) Dependence of ellipticity at 245 nm as a function of anion to peptide molar ratio. ■ experimental; – calculated.

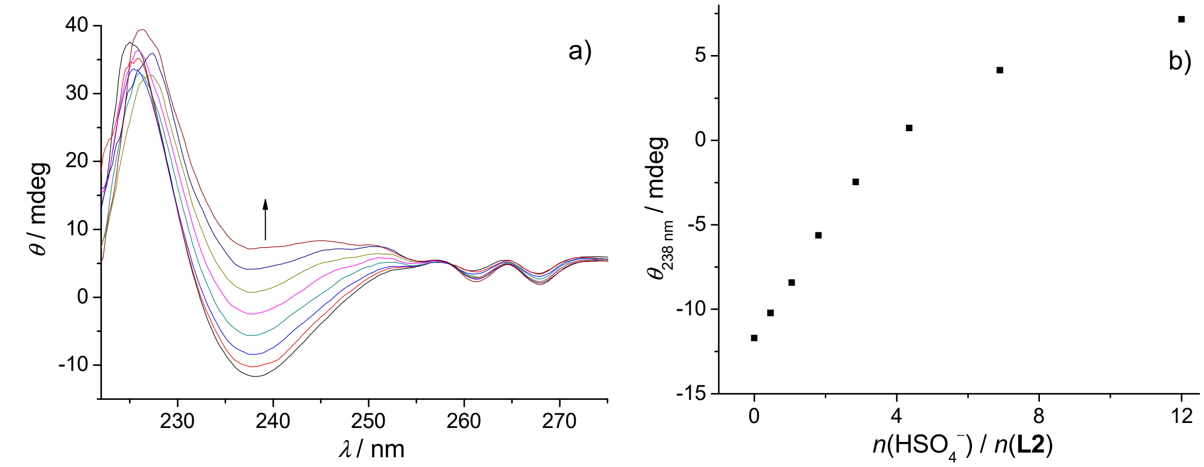

Figure S32. a) CD titration of **L2** (c = 5.00 × 10^{-4} mol dm^{-3}) with $TBAHSO_4$ (c = 0.100 mol dm^{-3}) in acetonitrile at 25.0 °C; V_0(**L2**) = 2.00 cm^3; l = 1 cm. b) Dependence of ellipticity at 238 nm as a function of anion to peptide molar ratio.

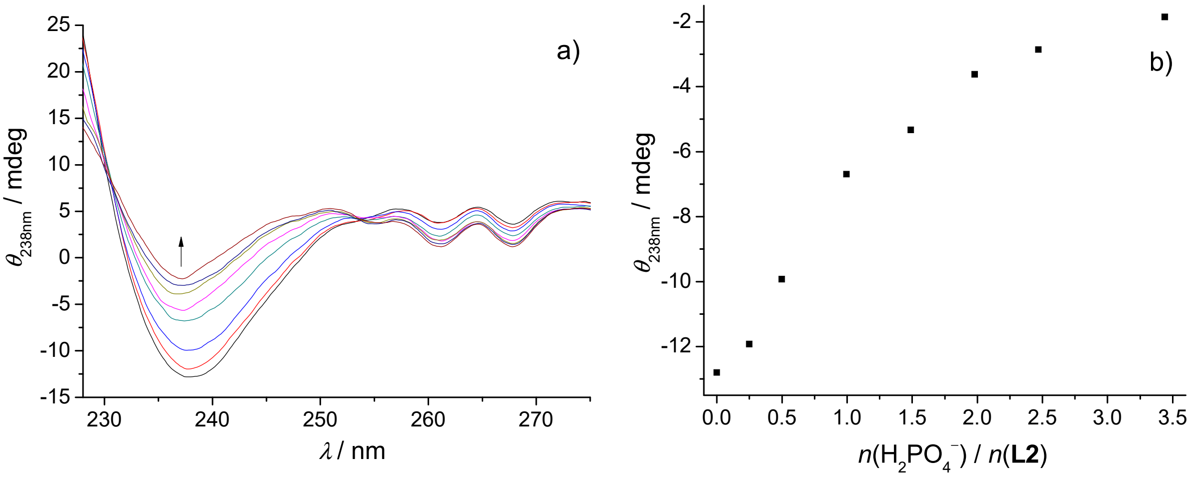

Figure S33. a) CD titration of **L2** (c = 5.00 × 10^{-4} mol dm^{-3}) with TBAH$_2$PO$_4$ (c = 0.100 mol dm^{-3}) in acetonitrile at 25.0 °C; V_0(**L2**) = 2.00 cm^3; l = 1 cm. b) Dependence of ellipticity at 238 nm as a function of anion to peptide molar ratio.

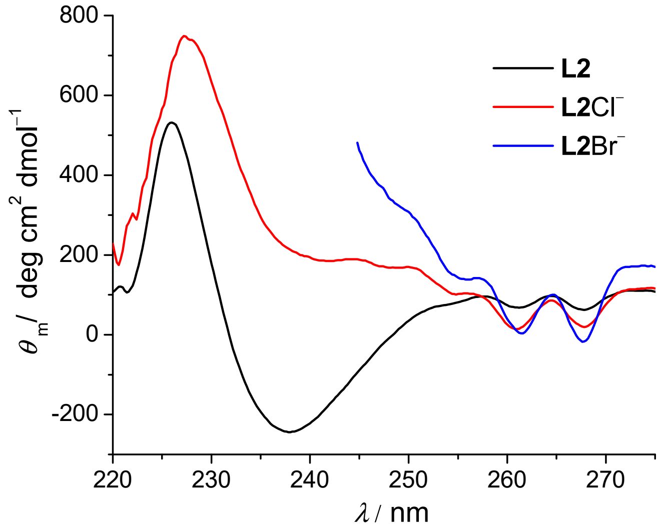

Figure S34. Molar CD spectra of **L2**-anion complexes in acetonitrile determined by CD titration experiments.

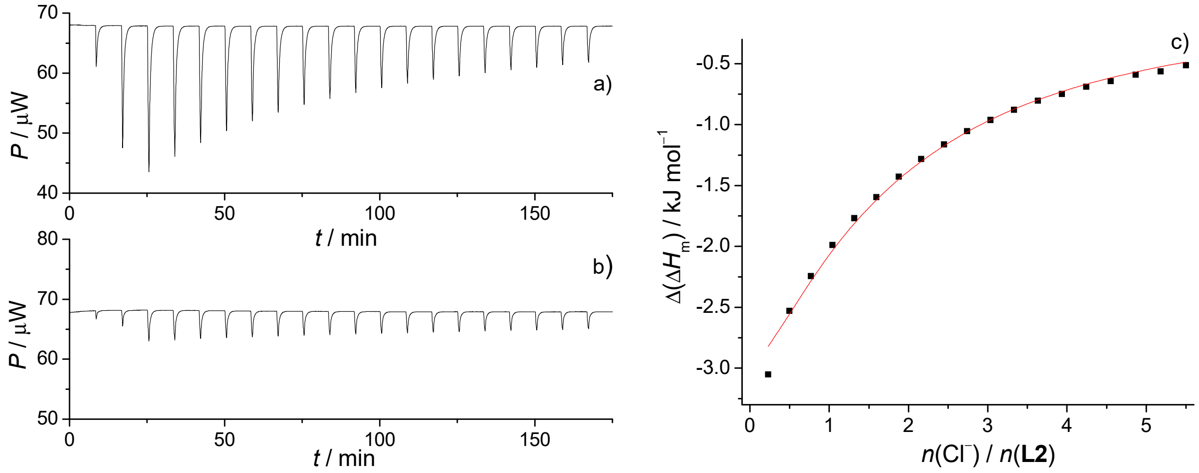

Figure S35. a) Microcalorimetric titration of **L2** ($c = 1.01 \times 10^{-3}$ mol dm^{-3}. $V = 1.4331$ cm^3) with TEACl ($c = 0.0251$ mol dm^{-3}) in acetonitrile; $\vartheta = 25$ °C. b) Microcalorimetric titration of acetonitrile with TEACl ($c = 0.0251$ mol dm^{-3}); $\vartheta = 25$ °C. c) Dependence of molar successive enthalpy change on $n(Cl^-)$ / n(**L2**) ratio. ■ experimental; — calculated.

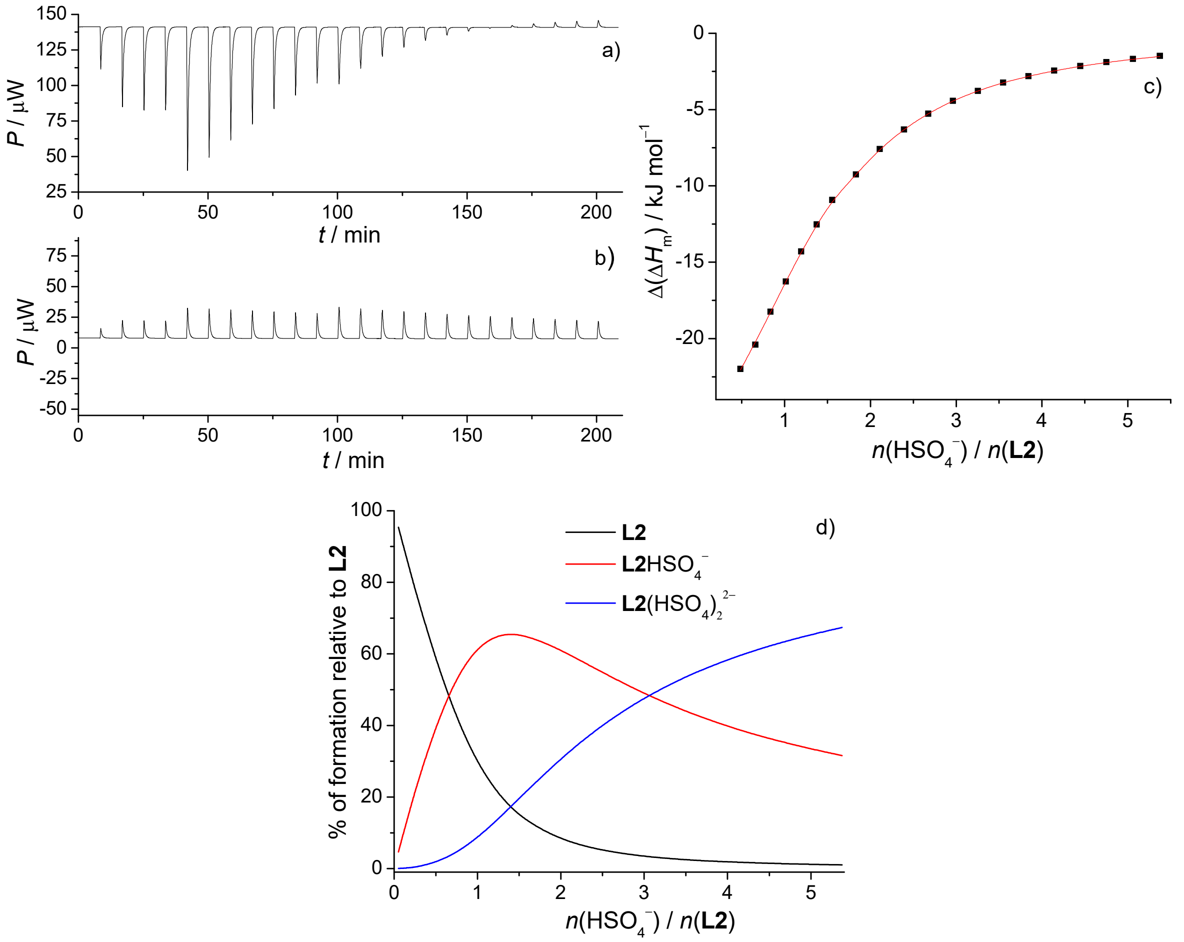

Figure S36. a) Microcalorimetric titration of **L2** ($c = 1.02 \times 10^{-3}$ mol dm^{-3}. $V = 1.4331$ cm^3) with TBAHSO$_4$ ($c = 0.249$ mol dm^{-3}) in acetonitrile; $\vartheta = 25$ °C. b) Microcalorimetric titration of acetonitrile with TBAHSO$_4$ ($c = 0.0249$ mol dm^{-3}); $\vartheta = 25$ °C. c) Dependence of molar successive enthalpy change on $n(HSO_4^-)$ / n(**L2**) ratio. ■ experimental; — calculated. d) Distribution of complex species during the titration of peptide **L2** with TBAHSO$_4$.

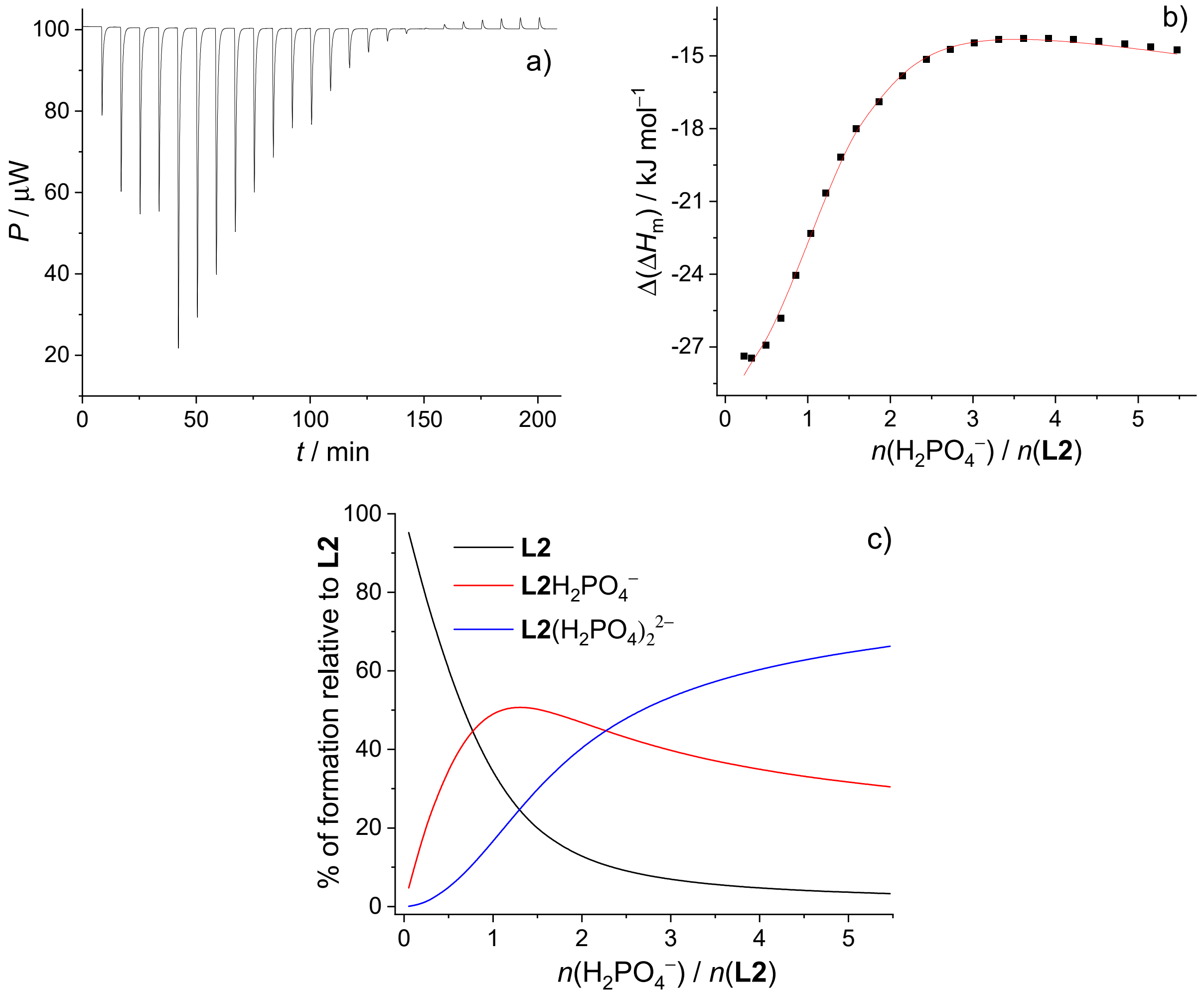

Figure S37. a) Microcalorimetric titration of **L2** ($c = 1.00 \times 10^{-3}$ mol dm^{-3}. $V = 1.4331$ cm^3) with TBAH_2PO_4 ($c = 0.0250$ mol dm^{-3}) in acetonitrile; $\vartheta = 25$ °C. b) Dependence of molar successive enthalpy change on $n(H_2PO_4^-)$ / n(**L2**) ratio. ■ experimental; — calculated. c) Distribution of complex species during the titration of peptide **L2** with TBAH_2PO_4.

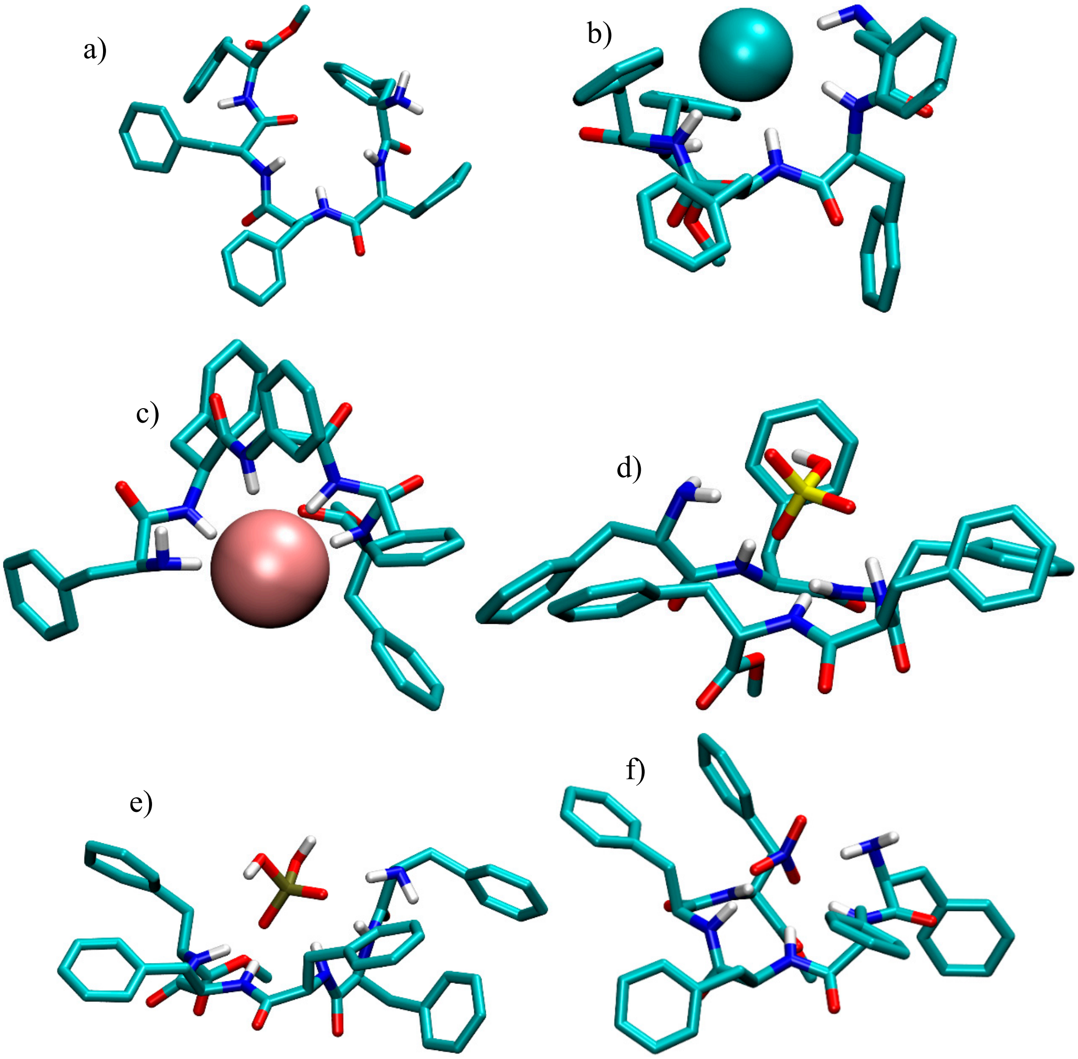

Figure S38. Representative structures of a) free peptide **L2** and its complexes with b) Cl^{-}, c) Br^{-}, d) HSO_4^{-}, e) $H_2PO_4^{-}$, and f) NO_3^{-} in acetonitrile obtained by MD simulations in acetonitrile. Peptide hydrogen atoms bound to carbon atoms are omitted for clarity.

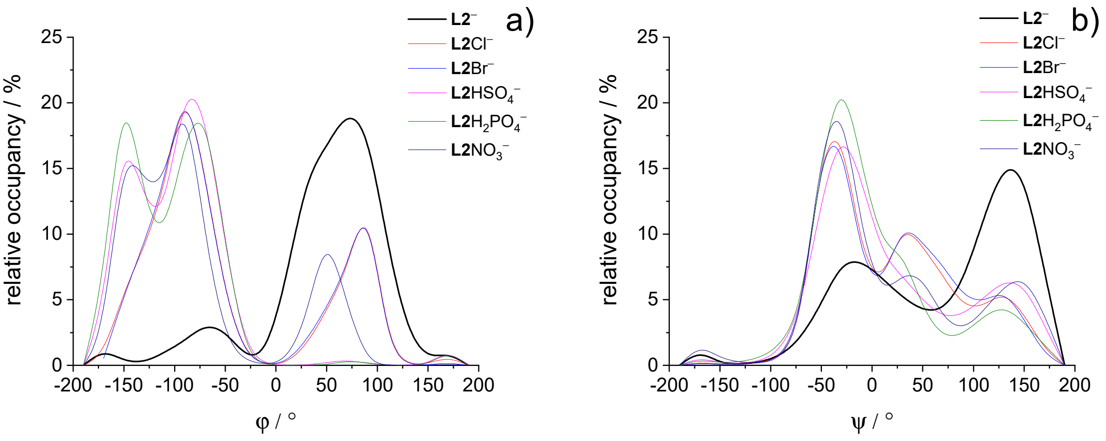

Figure S39. Distribution of a) *φ*, b) *ψ* angles of free peptide **L2** and its complexes with anions obtained by MD simulation in acetonitrile at 25 °C.

Table S3. Energies of interactions between different species (**L2**, $A^{–}$, and MeCN) obtained by MD simulations in acetonitrile at 25 °C.

	free	$Cl^{–}$	$Br^{–}$	$HSO_4^{–}$	$H_2PO_4^{–}$	$NO_3^{–}$
E(**L2**–$A^{–}$) / kJ $mol^{–1}$	–	–226	–214	–161	–252	–203
E(**L2**–MeCN) / kJ $mol^{–1}$	–460	–343	–342	–363	–349	–353
E($A^{–}$–MeCN) / kJ $mol^{–1}$	–	–59	–56	–79	–78	–61

Table S4. Distances between nitrogen atom of N terminus and carbon atom of C terminus on free peptide **L2** and its complexes with anions obtained by MD simulations in acetonitrile at 25 °C.

	free	$Cl^{–}$	$Br^{–}$	$HSO_4^{–}$	$H_2PO_4^{–}$	$NO_3^{–}$
d(C–N) / Å	11.7 ± 2.3	7.9 ± 1.2	8.0 ± 1.0	8.6 ± 2.0	7.1 ± 1.3	7.3 ± 1.2

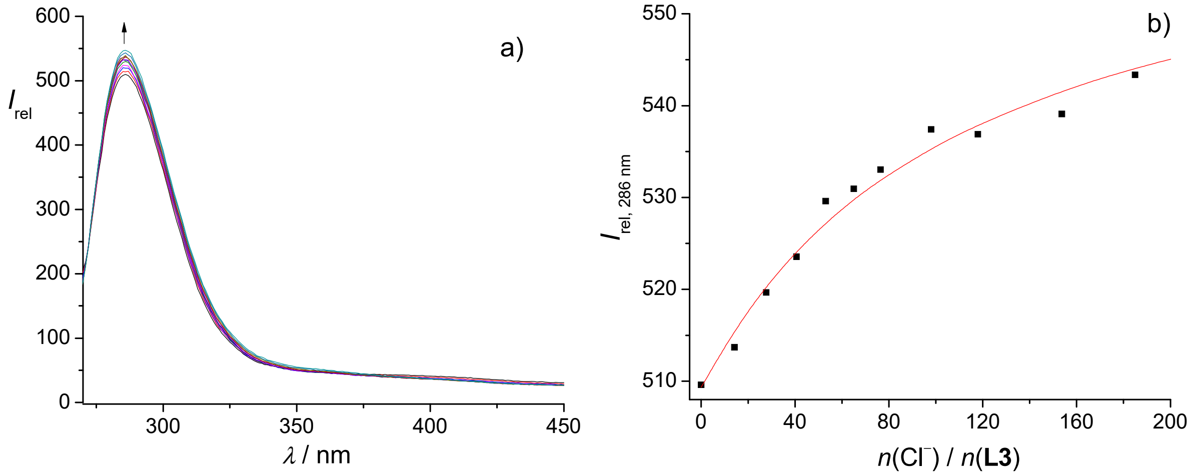

Figure S40. a) Spectrofluorimetric titration of **L3** ($c = 1.57 \times 10^{-4}$ mol dm^{-3}) with TEACl ($c = 0.100$ mol dm^{-3}) in acetonitrile at 25.0 °C; V_0(**L3**) = 2.20 cm^3; $\lambda_{ex} = 260$ nm; excitation slit 5 nm, emission slit 10 nm. Spectra are corrected for dilution. b) Relative fluorescence intensity at 286 nm as a function of anion to peptide molar ratio. ■ experimental; – calculated.

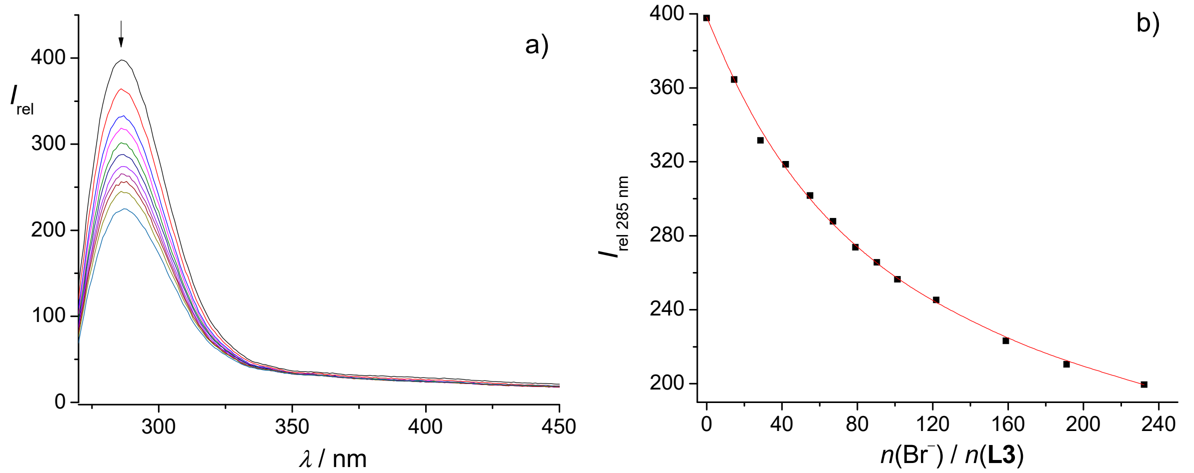

Figure S41. a) Spectrofluorimetric titration of **L3** ($c = 1.24 \times 10^{-4}$ mol dm^{-3}) with TBABr ($c = 0.082$ mol dm^{-3}) in acetonitrile at 25.0 °C; V_0(**L3**) = 2.20 cm^3; $\lambda_{ex} = 260$ nm; excitation slit 5 nm, emission slit 10 nm. Spectra are corrected for dilution. b) Relative fluorescence intensity at 285 nm as a function of anion to peptide molar ratio. ■ experimental; – calculated.

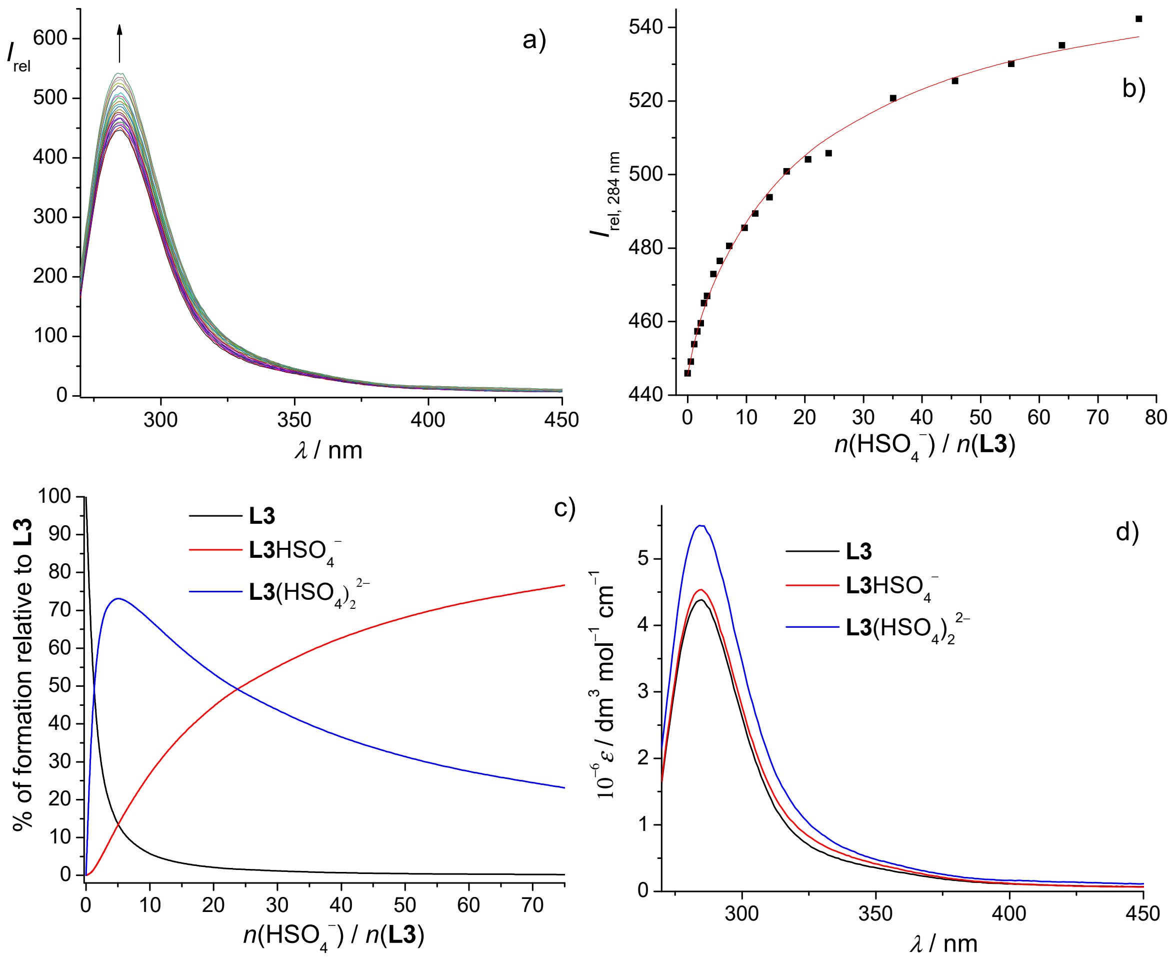

Figure S42. a) Spectrofluorimetric titration of **L3** ($c = 1.02 \times 10^{-4}$ mol dm^{-3}) with **L3** containing solution of $TBAHSO_4$ ($c = 0.025$ mol dm^{-3}) in acetonitrile at 25.0 °C; V_0(**L3**) = 2.20 cm^3; λ_{ex} = 260 nm; excitation slit 5 nm, emission slit 10 nm. Spectra are corrected for dilution. b) Relative fluorescence intensity at 285 nm as a function of anion to peptide molar ratio. ■ experimental; – calculated. c) Distribution of complex species during the titration of peptide **L3** with $TBAHSO_4$. d) Molar spectra of free peptide **L3** and its complexes with HSO_4^- anion.

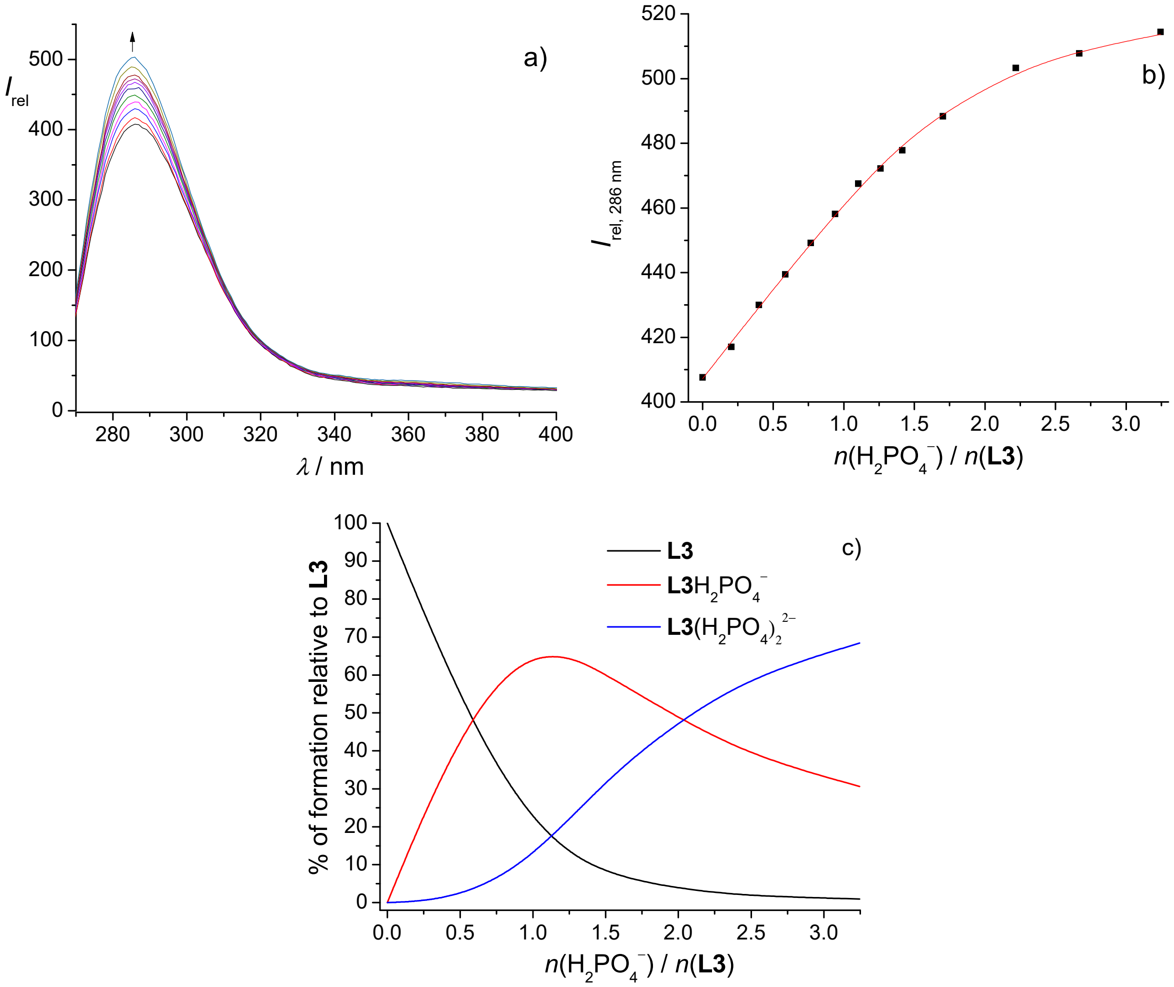

Figure S43. a) Spectrofluorimetric titration of **L3** ($c = 1.24 \times 10^{-4}$ mol dm^{-3}) with TBAH$_2$PO$_4$ ($c = 1.14 \times 10^{-3}$ mol dm^{-3}) in acetonitrile at 25.0 °C; V_0(**L3**) = 2.20 cm^3; λ_{ex} = 260 nm; excitation slit 5 nm, emission slit 10 nm. Spectra are corrected for dilution. b) Relative fluorescence intensity at 286 nm as a function of anion to peptide molar ratio. ■ experimental; – calculated. c) Distribution of complex species during the titration of peptide **L3** with TBAH$_2$PO$_4$.

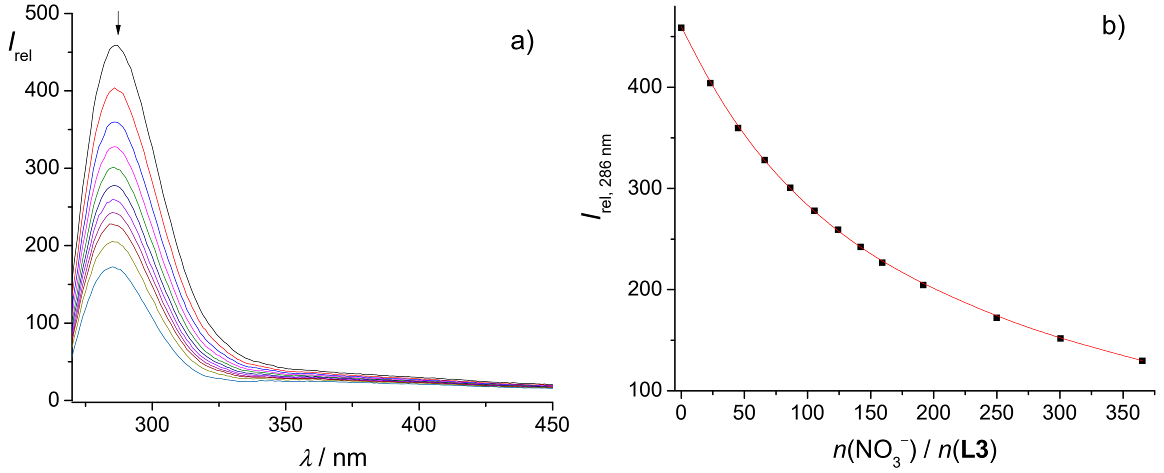

Figure S44. a) Spectrofluorimetric titration of **L3** (c = 1.57 × 10^{-4} mol dm^{-3}) with TBANO$_3$ (c = 0.11 mol dm^{-3}) in acetonitrile at 25.0 °C; V_0(**L3**) = 2.20 cm^3; λ_{ex} = 260 nm; excitation slit 5 nm, emission slit 10 nm. Spectra are corrected for dilution. b) Relative fluorescence intensity at 286 nm as a function of anion to peptide molar ratio. ■ experimental; – calculated.

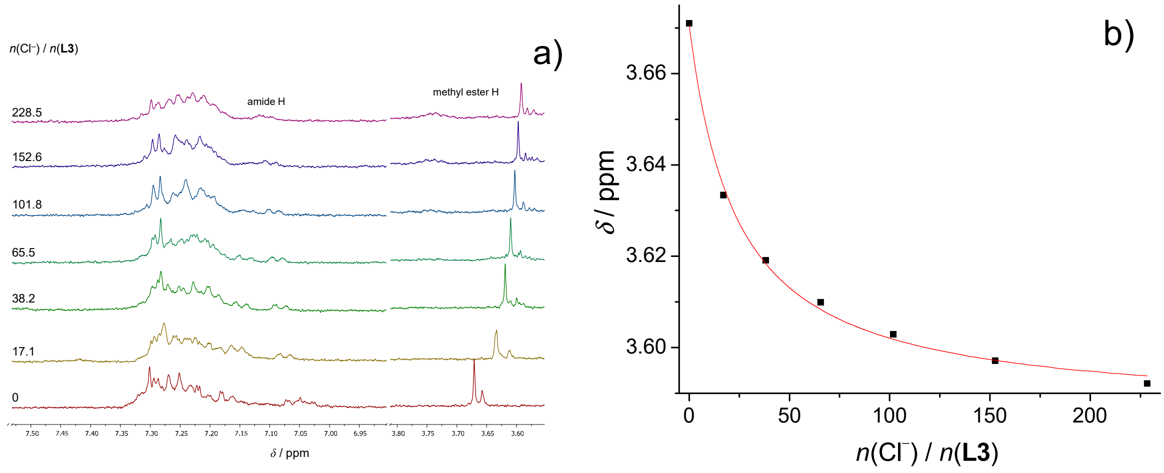

Figure S45. a) ^{1}H NMR titration of **L3** (c = 1.09 × 10^{-4} mol dm^{-3}) with TEACl (c = 0.061 mol dm^{-3}) in deuterated acetonitrile at 25.0 °C; V_0(**L3**) = 0.50 cm^3. b) Chemical shift of methyl ester protons at 3.67 ppm as a function of anion to peptide molar ratio. ■ experimental; – calculated.

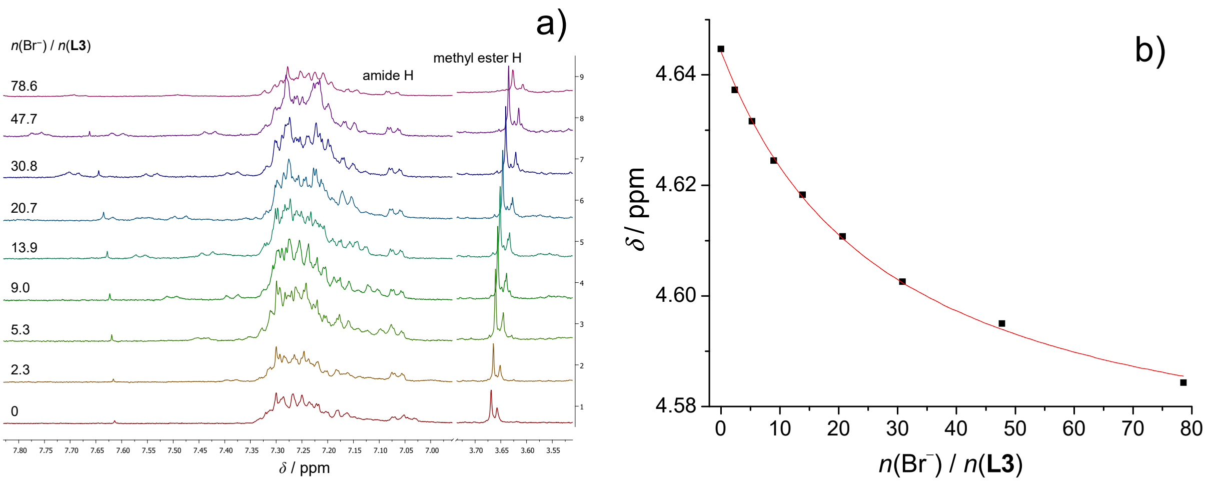

Figure S46. a) ^{1}H NMR titration of **L3** ($c = 4.91 \times 10^{-4}$ mol dm^{-3}) with TBABr ($c = 0.083$ mol dm^{-3}) in deuterated acetonitrile at 25.0 °C; V_0(**L3**) = 0.50 cm^3. b) Chemical shift of C–α protons at 4.65 ppm as a function of anion to peptide molar ratio. ■ experimental; – calculated.

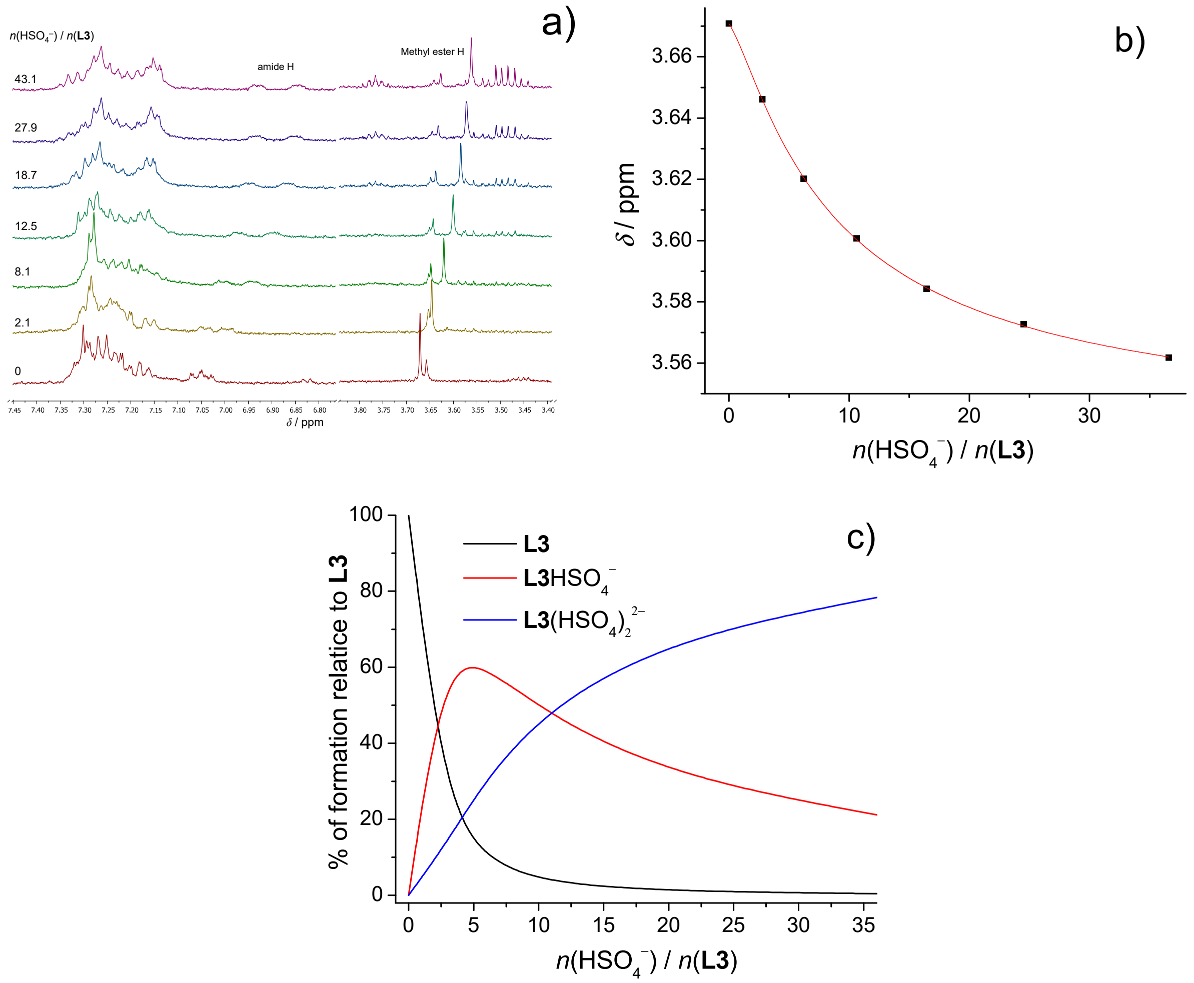

Figure S47. a) ^{1}H NMR titration of **L3** ($c = 1.04 \times 10^{-4}$ mol dm^{-3}) with TBAHSO$_4$ ($c = 9.87 \times 10^{-3}$ mol dm^{-3}) in deuterated acetonitrile at 25.0 °C; V_0(**L3**) = 0.50 cm^3. b) Chemical shift of methyl ester protons at 3.67 ppm as a function of anion to peptide molar ratio. ■ experimental; – calculated. c) Distribution of complex species during the titration of peptide **L3** with TBAHSO$_4$.

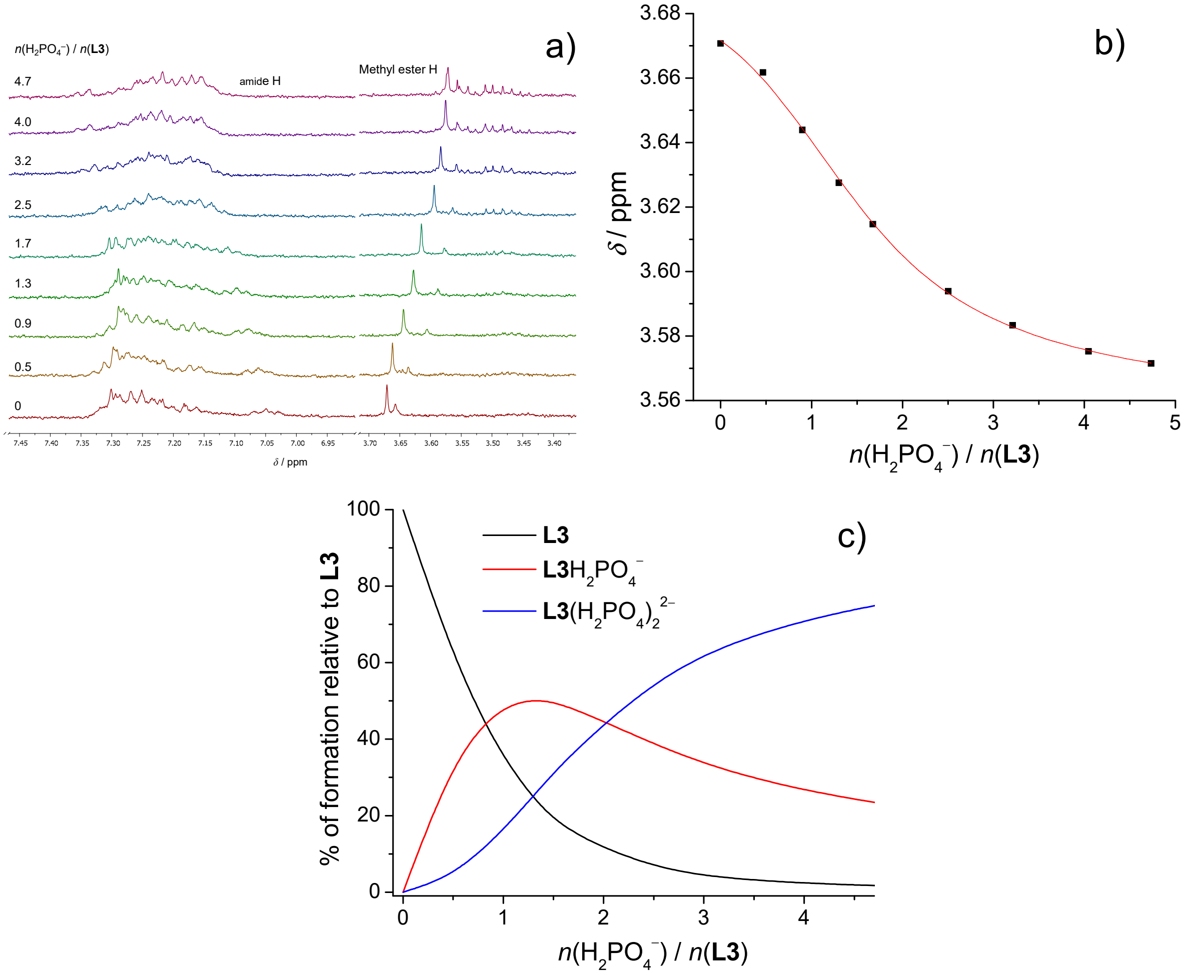

Figure S48. a) ^{1}H NMR titration of **L3** ($c = 9.56 \times 10^{-5}$ mol dm^{-3}) with TBAH$_2$PO$_4$ ($c = 1.54 \times 10^{-3}$ mol dm^{-3}) in deuterated acetonitrile at 25.0 °C; V_0(**L3**) = 0.50 cm^3. b) Chemical shift of methyl ester protons at 3.67 ppm as a function of anion to peptide molar ratio. ■ experimental; – calculated. c) Distribution of complex species during the titration of peptide **L3** with TBAH$_2$PO$_4$.

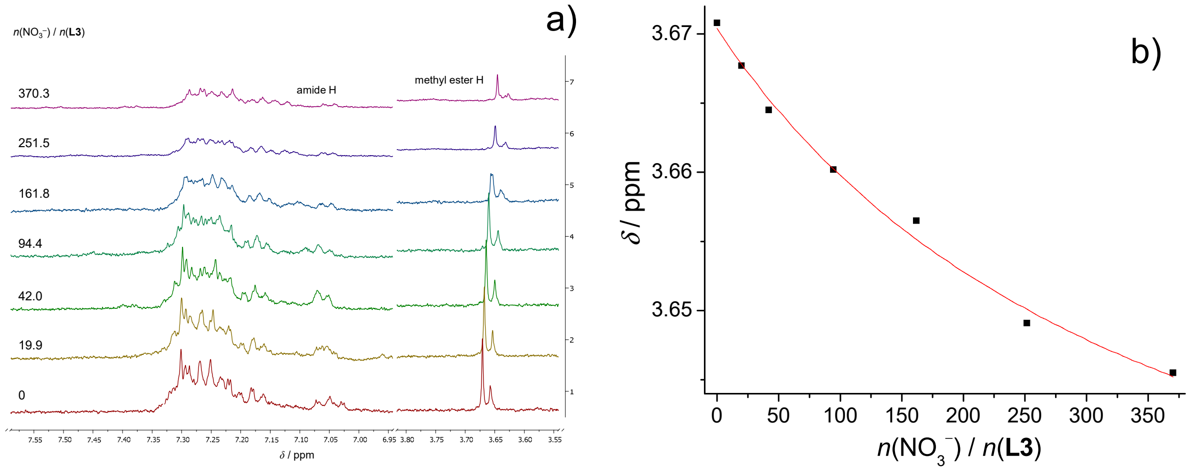

Figure S49. a) ^{1}H NMR titration of **L3** ($c = 2.05 \times 10^{-4}$ mol dm^{-3}) with TBANO$_3$ ($c = 0.15$ mol dm^{-3}) in deuterated acetonitrile at 25.0 °C; V_0(**L3**) = 0.50 cm^3. b) Chemical shift of methyl ester protons at 3.67 ppm as a function of anion to peptide molar ratio. ■ experimental; – calculated.

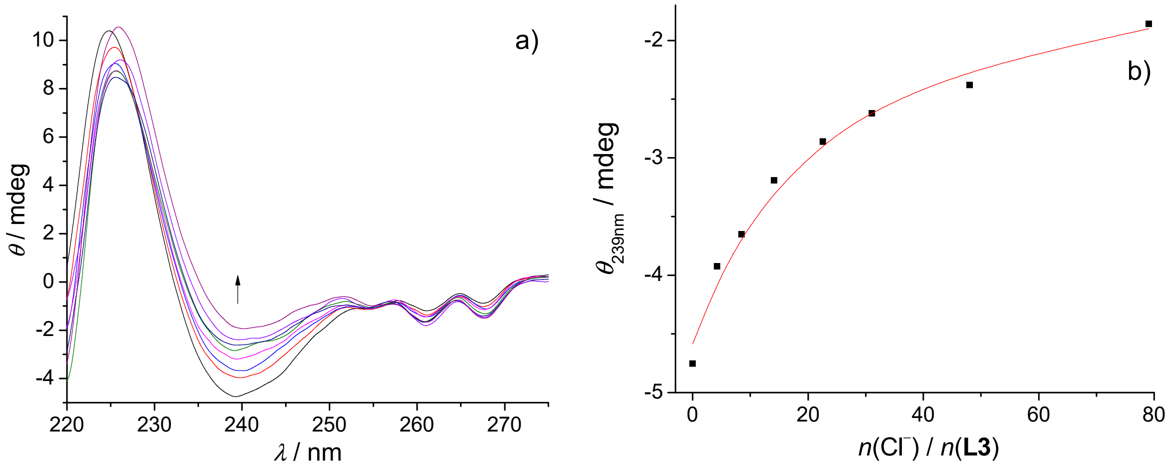

Figure S50. a) CD titration of **L3** ($c = 1.08 \times 10^{-4}$ mol dm^{-3}) with TEACl ($c = 0.061$ mol dm^{-3}) in acetonitrile at 25.0 °C; V_0(**L3**) = 2.00 cm^3; $l = 1$ cm. b) Dependence of ellipticity at 239 nm as a function of anion to peptide molar ratio. ■ experimental; – calculated.

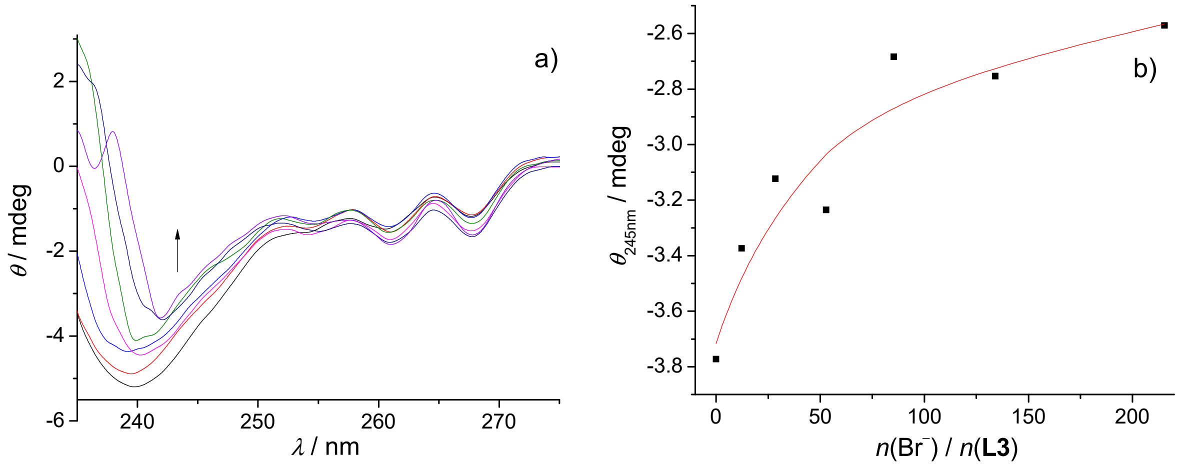

Figure S51. a) CD titration of **L3** ($c = 1.08 \times 10^{-4}$ mol dm^{-3}) with TBABr ($c = 0.088$ mol dm^{-3}) in acetonitrile at 25.0 °C; V_0(**L3**) = 2.00 cm^3; l = 1 cm. b) Dependence of ellipticity at 245 nm as a function of anion to peptide molar ratio. ■ experimental; – calculated.

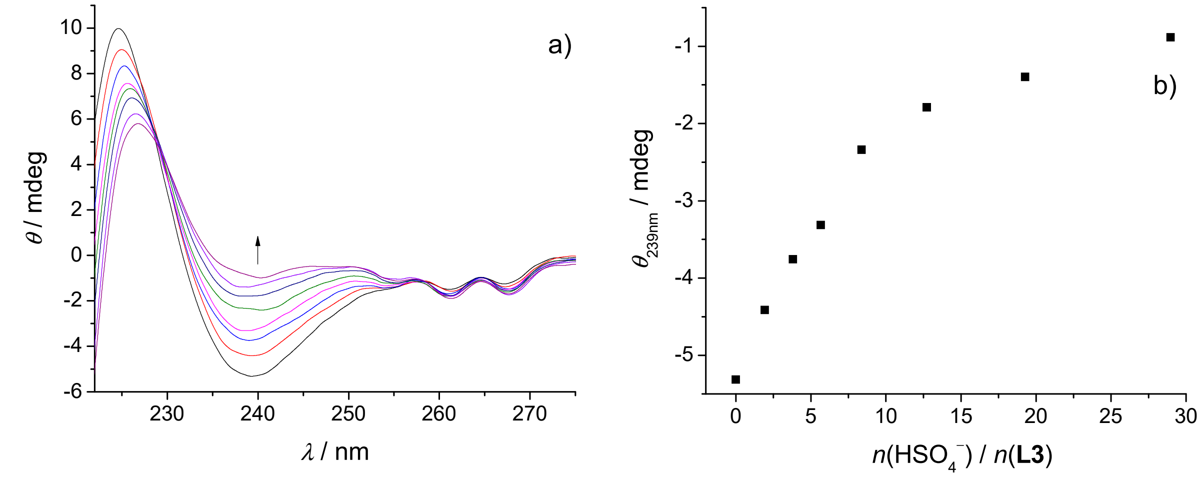

Figure S52. a) CD titration of **L3** ($c = 1.08 \times 10^{-4}$ mol dm^{-3}) with TBAHSO$_4$ ($c = 0.021$ mol dm^{-3}) in acetonitrile at 25.0 °C; V_0(**L3**) = 2.00 cm^3; l = 1 cm. b) Dependence of ellipticity at 239 nm as a function of anion to peptide molar ratio.

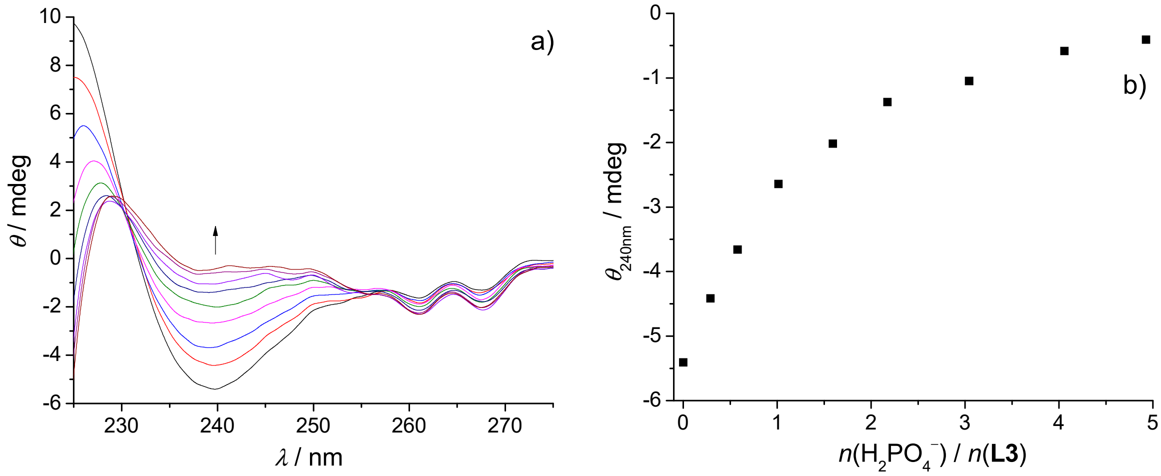

Figure S53. a) CD titration of **L3** (c = 1.08 × 10^{-4} mol dm^{-3}) with TBAH_2PO_4 (c = 3.13 × 10^{-3} mol dm^{-3}) in acetonitrile at 25.0 °C; V_0(**L3**) = 2.00 cm^3; l = 1 cm. b) Dependence of ellipticity at 240 nm as a function of anion to peptide molar ratio.

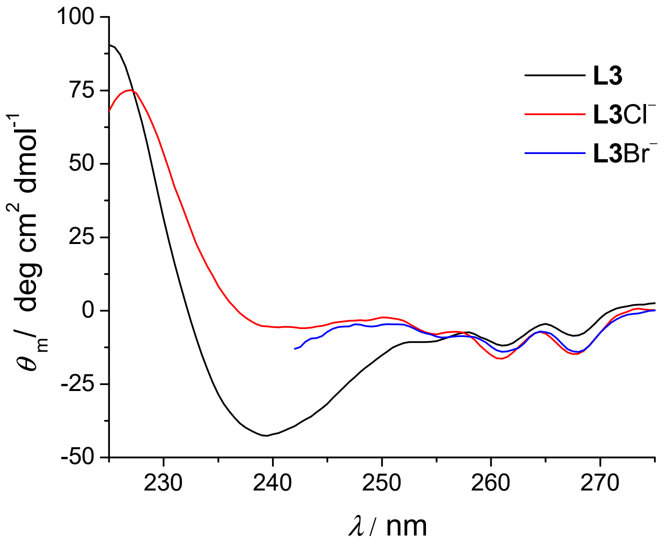

Figure S54. Molar CD spectra of **L3**-anion complexes in acetonitrile determined by CD titration experiments.

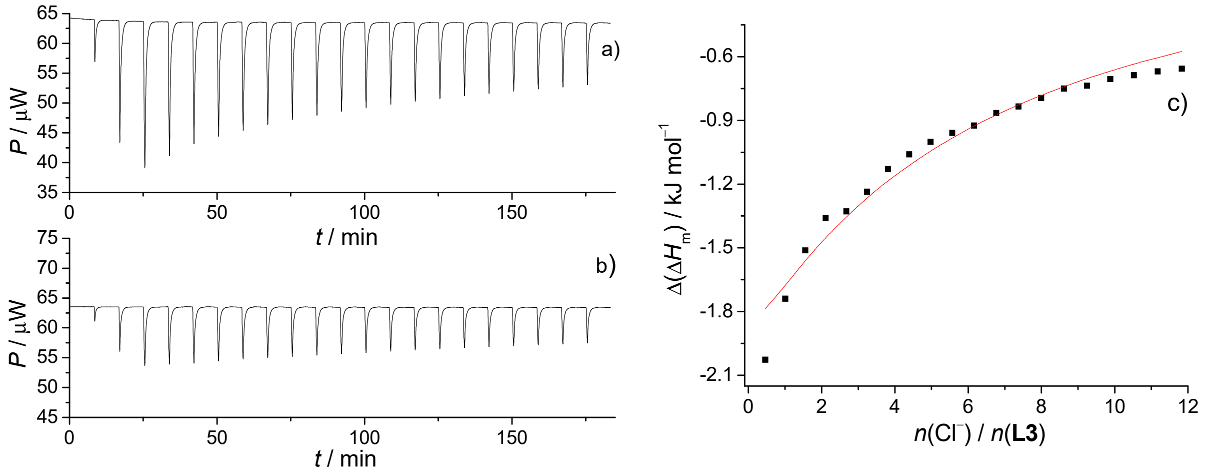

Figure S55. a) Microcalorimetric titration of **L3** (c = 4.92 × 10^{-4} mol dm^{-3}. V = 1.4295 cm^3) with TEACl (c = 0.0250 mol dm^{-3}) in acetonitrile; ϑ = 25 °C. b) Microcalorimetric titration of acetonitrile with TEACl (c = 0.0250 mol dm^{-3}); ϑ = 25 °C. c) Dependence of molar successive enthalpy change on n(Cl$^-$) / n(**L3**) ratio. ■ experimental; — calculated.

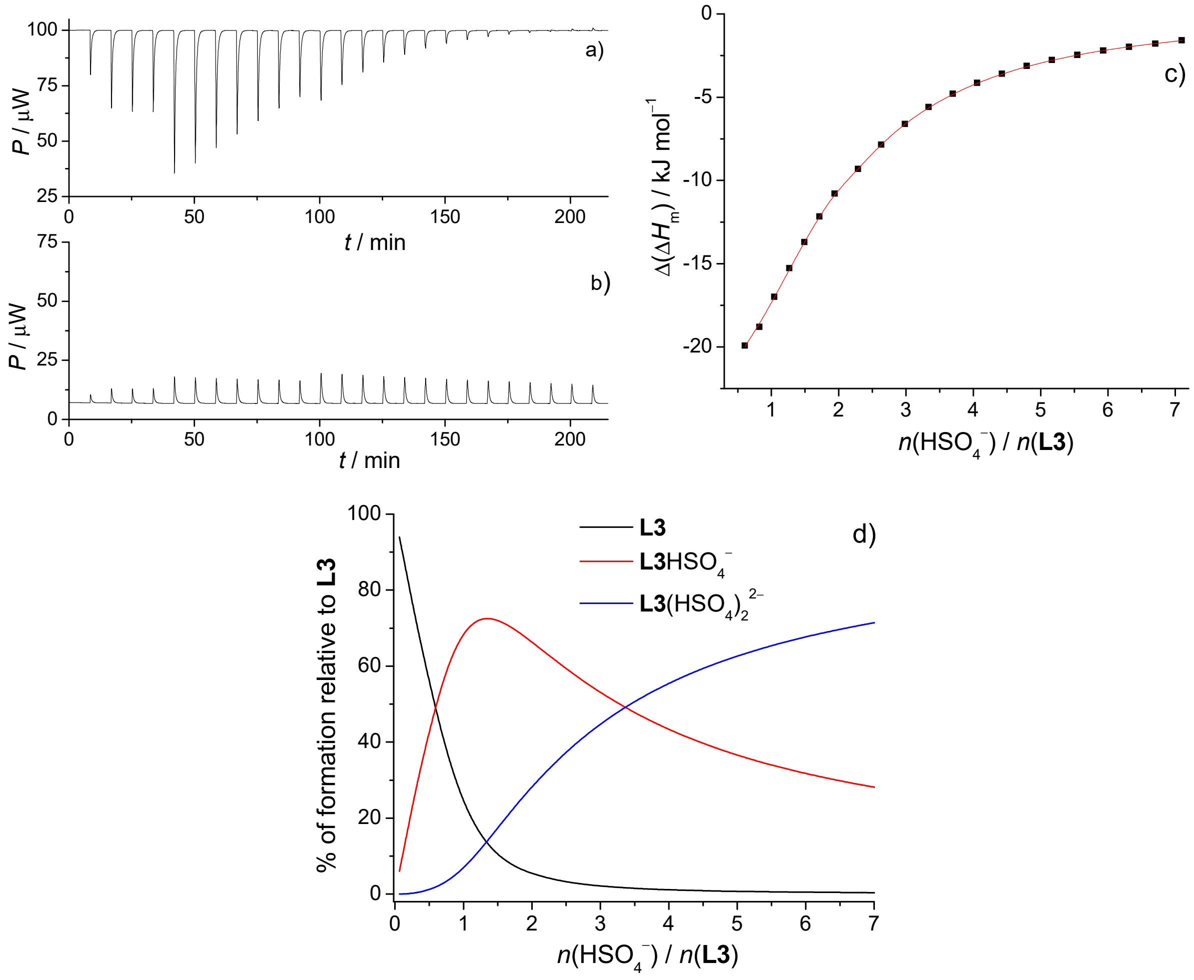

Figure S56. a) Microcalorimetric titration of **L3** ($c = 4.92 \times 10^{-4}$ mol dm^{-3}. $V = 1.4295$ cm^{3}) with TBAHSO$_4$ (c = 0.0150 mol dm^{-3}) in acetonitrile; ϑ = 25 °C. b) Microcalorimetric titration of acetonitrile with TBAHSO$_4$ (c = 0.0150 mol dm^{-3}); ϑ = 25 °C. c) Dependence of molar successive enthalpy change on $n(HSO_4^-)$ / n(**L3**) ratio. ■ experimental; — calculated. d) Distribution of complex species during the titration of peptide **L3** with TBAHSO$_4$.

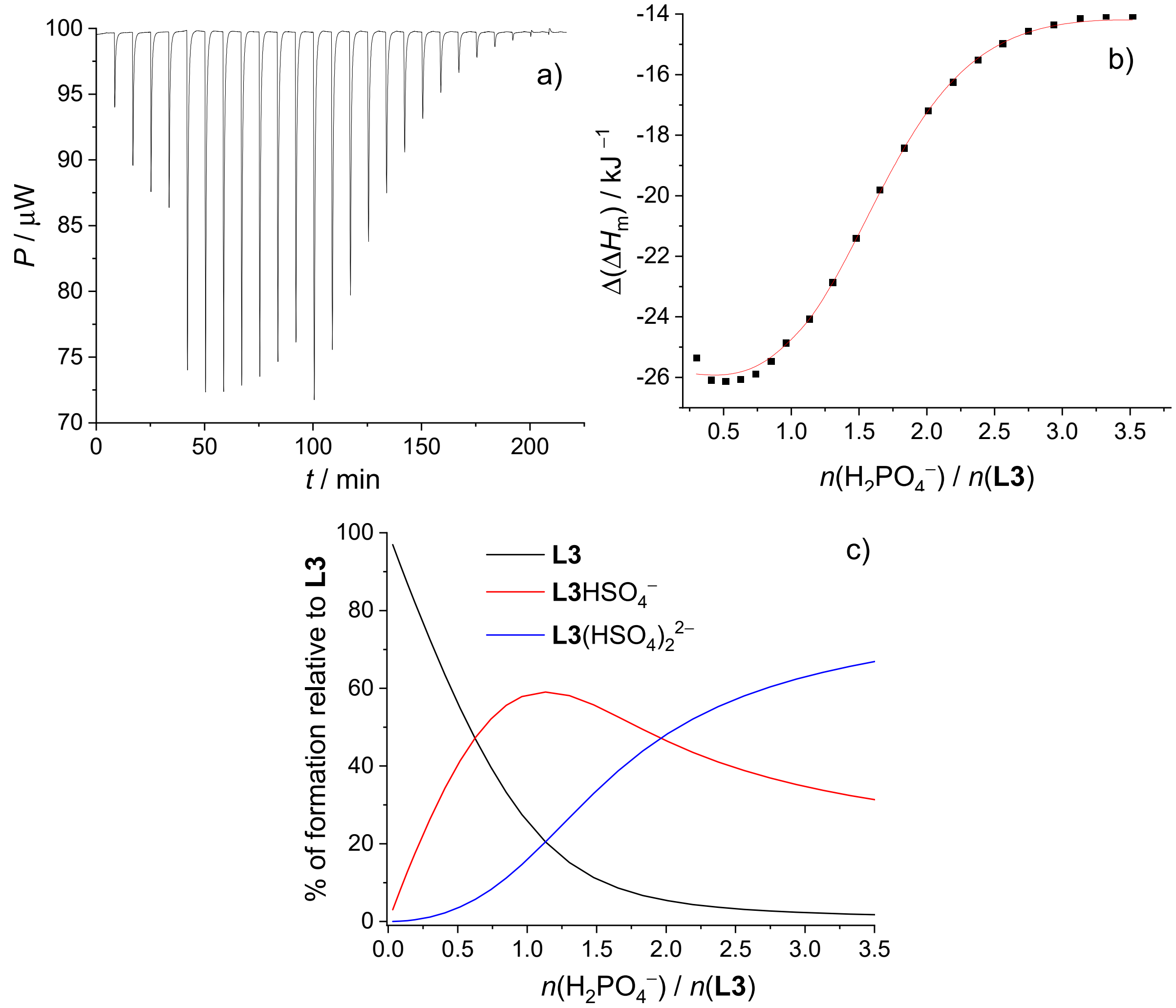

Figure S57. a) Microcalorimetric titration of **L3** ($c = 4.87 \times 10^{-4}$ mol dm^{-3}. $V = 1.4295$ cm^3) with TBAH$_2$PO$_4$ ($c = 7.36 \times 10^{-3}$ mol dm^{-3}) in acetonitrile; $\vartheta = 25$ °C. b) Dependence of molar successive enthalpy change on $n(H_2PO_4^-)$ / n(**L3**) ratio. ■ experimental; — calculated. c) Distribution of complex species during the titration of peptide **L3** with TBAH$_2$PO$_4$.

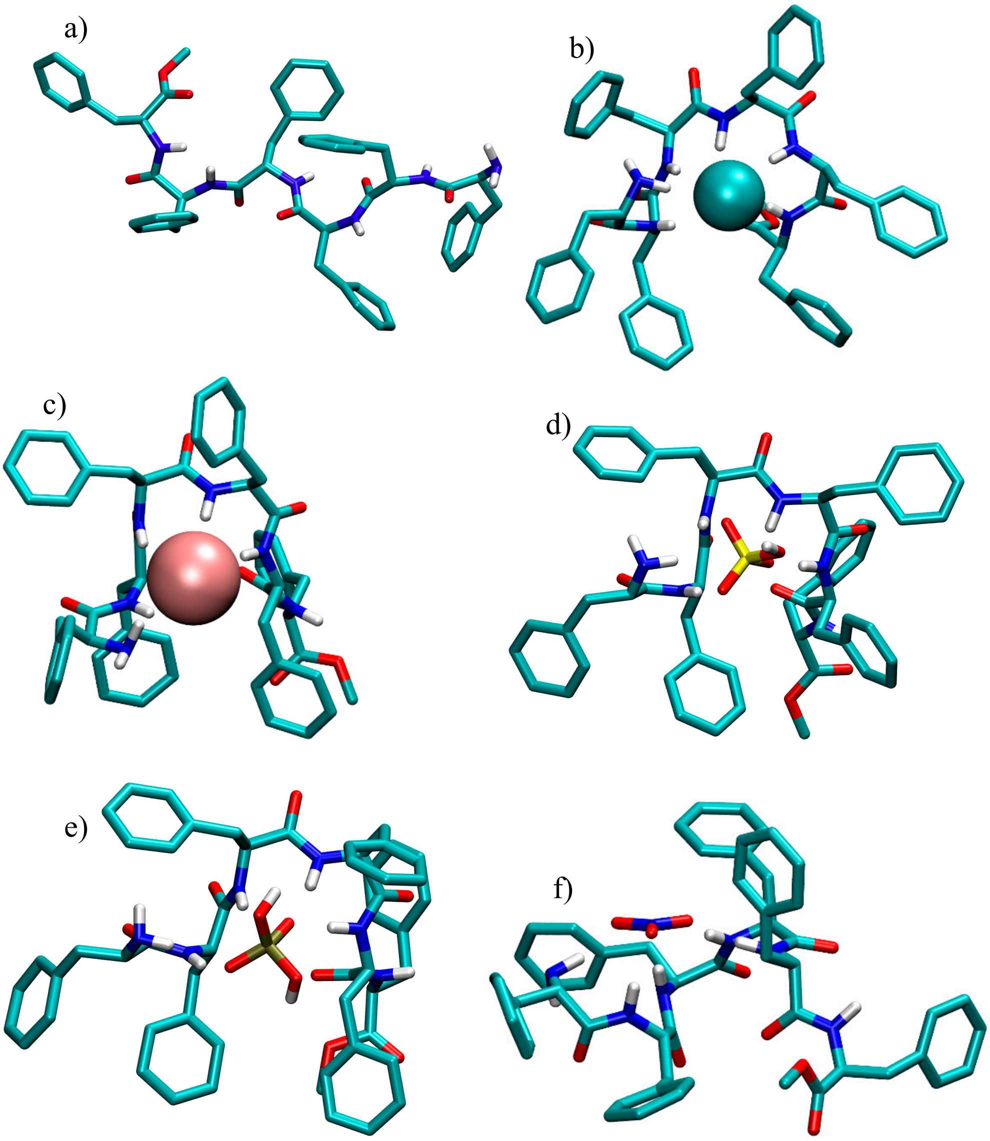

Figure S58. Representative structures of a) free peptide **L3** and its complexes with b) Cl^{-}, c) Br^{-}, d) HSO_4^{-}, e) $H_2PO_4^{-}$, and f) NO_3^{-} in acetonitrile obtained by MD simulations in acetonitrile. Peptide hydrogen atoms bound to carbon atoms are omitted for clarity.

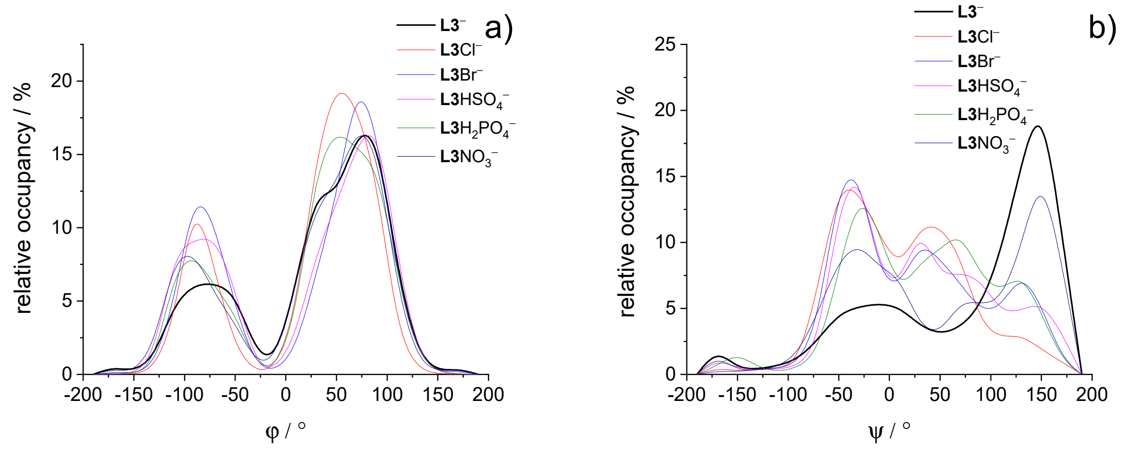

Figure S59. Distribution of a) φ, b) ψ angles of free peptide **L3** and its complexes with anions obtained by MD simulation in acetonitrile at 25 °C.

Table S5. Energies of interactions between different species (**L3**, A^-, and MeCN) obtained by MD simulations in acetonitrile at 25 °C.

	free	Cl^-	Br^-	HSO_4^-	$H_2PO_4^-$	NO_3^-
E(**L3**–A^-) / kJ mol^{-1}	–	–270	–228	–225	–275	–128
E(**L3**–MeCN) / kJ mol^{-1}	–549	–397	–403	–417	–424	–446
E(A^-–MeCN) / kJ mol^{-1}	–	–49	–55	–80	–77	–109

Table S6. Distances between nitrogen atom of N terminus and carbon atom of C terminus on free peptide **L3** and its complexes with anions obtained by MD simulations in acetonitrile at 25 °C.

	free	Cl^-	Br^-	HSO_4^-	$H_2PO_4^-$	NO_3^-
d(C–N) / Å	13.3 ± 3.6	8.1 ± 0.9	9.3 ± 1.4	10.4 ± 1.2	10.0 ± 1.6	9.3 ± 3.1

Anion complexation by peptides in DMF

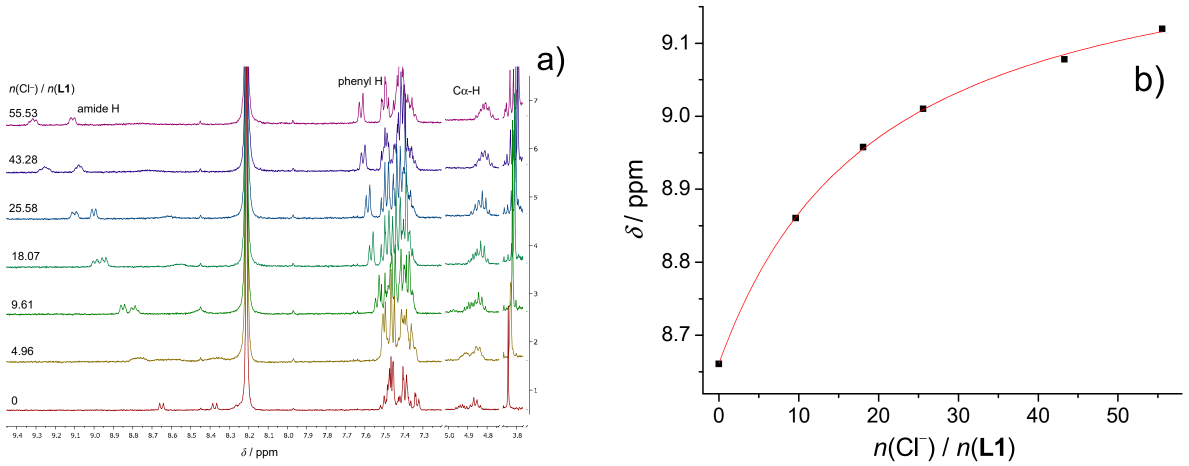

Figure S60. a) ^{1}H NMR titration of **L1** (c = 5.31 × 10^{-4} mol dm^{-3}) with TEACl (c = 0.0825 mol dm^{-3}) in deuterated DMF at ϑ = 25 °C; V_0 (**L1**) = 0.50 cm^{3}. b) The dependence of chemical shift of **L1** amide protons at 8.65 ppm on n(Cl^{-}) / n(**L1**) molar ratio.

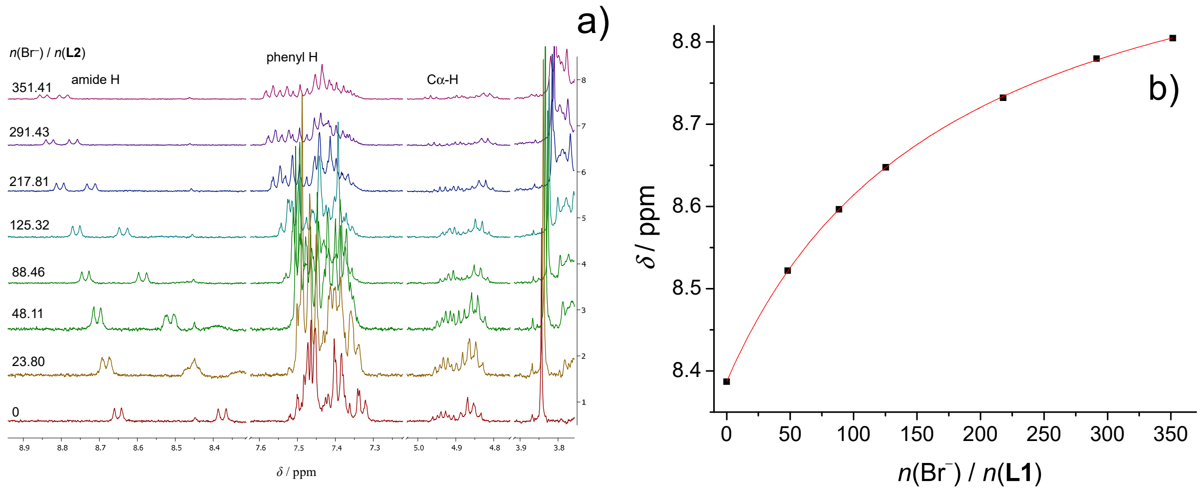

Figure S61. a) ^{1}H NMR titration of **L1** (c = 5.31 × 10^{-4} mol dm^{-3}) with TBABr (c = 0.513 mol dm^{-3}) in deuterated DMF at ϑ = 25 °C; V_0 (**L1**) = 0.50 cm^{3}. b) The dependence of chemical shift of **L1** amide protons at 8.40 ppm on n(Br^{-}) / n(**L1**) molar ratio (right).

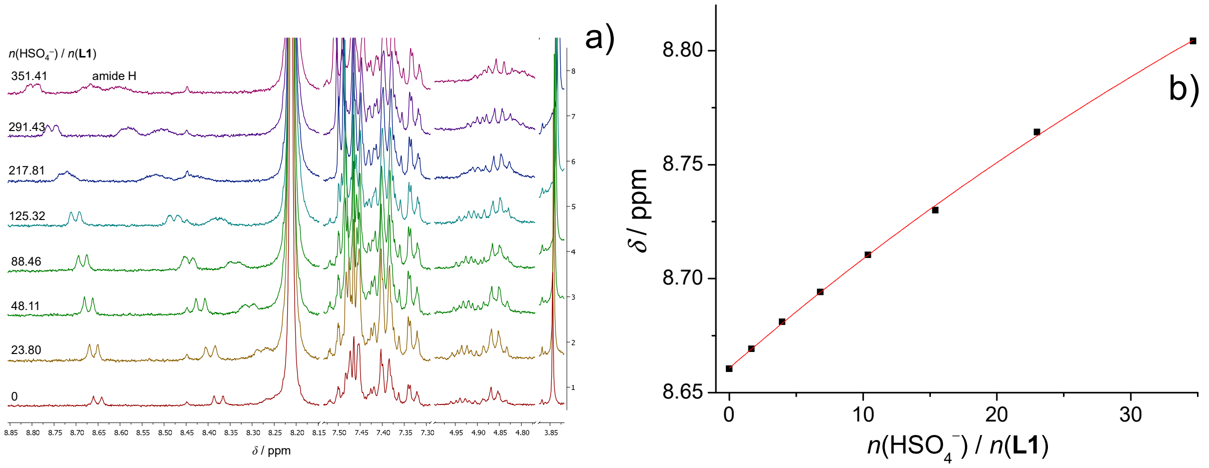

Figure S62. a) ^{1}H NMR titration of **L1** (c = 4.36 × 10^{-4} mol dm^{-3}) with TBAHSO4 (c = 0.0406 mol dm^{-3}) in deuterated DMF at ϑ = 25 °C; V_0 (**L1**) = 0.50 cm^3. b) The dependence of chemical shift of **L1** amide protons at 8.65 ppm on $n(HSO_4^-)$ / n(**L1**) molar ratio.

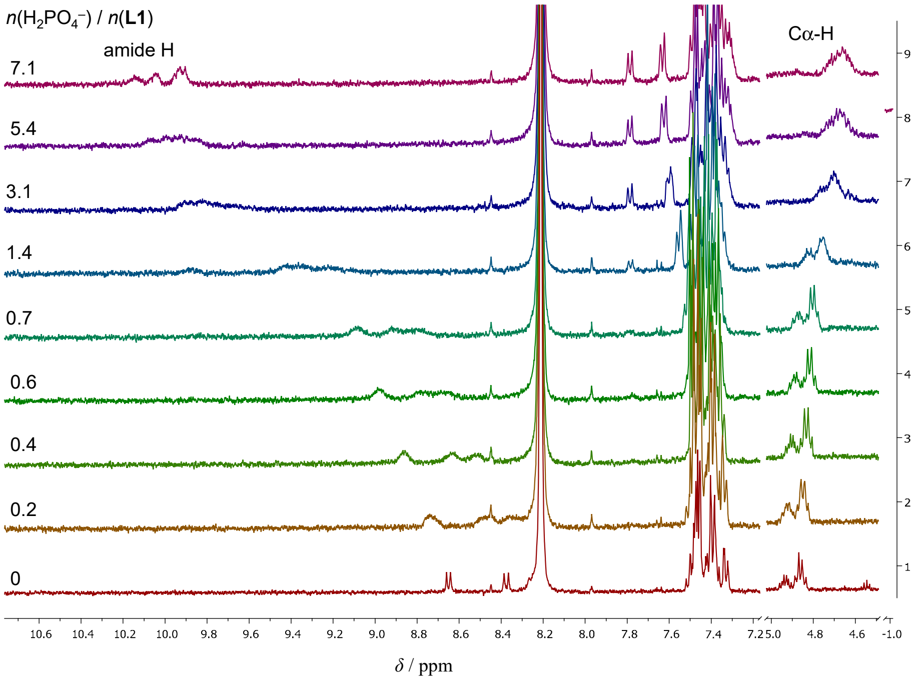

Figure S63. ^{1}H NMR titration of **L1** (c = 4.36 × 10^{-4} mol dm^{-3}) with TBAH2PO4 (c = 8.15 × 10^{-3} mol dm^{-3}) in deuterated DMF at ϑ = 25 °C; V_0 (**L1**) = 0.50 cm^3.

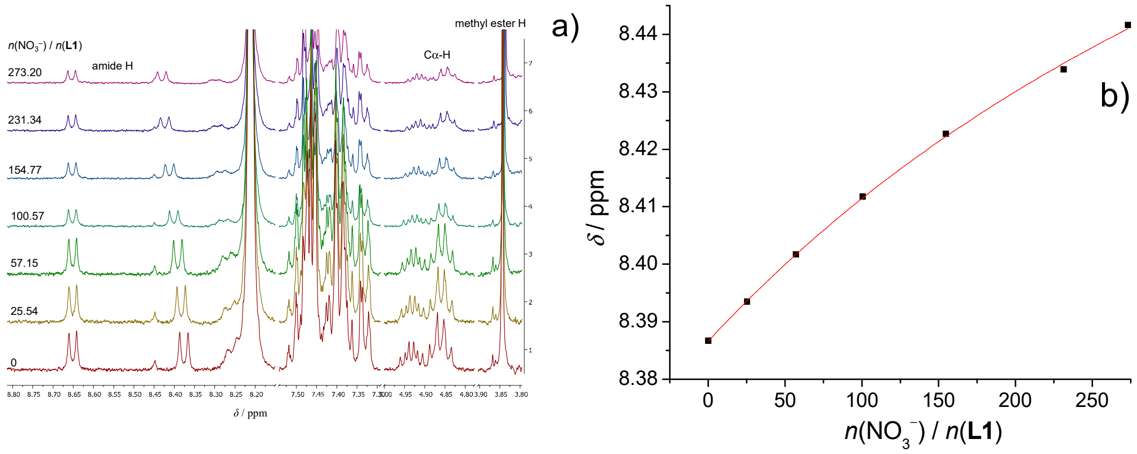

Figure S64. a) ^{1}H NMR titration of **L1** (c = 4.36 × 10^{-4} mol dm^{-3}) with $TBANO_3$ (c = 0.299 mol dm^{-3}) in deuterated DMF at ϑ = 25 °C; V_0 (**L1**) = 0.50 cm^3. b) The dependence of chemical shift of **L1** amide protons at 8.38 ppm on $n(NO_3^-)$ / n(**L1**) molar ratio.

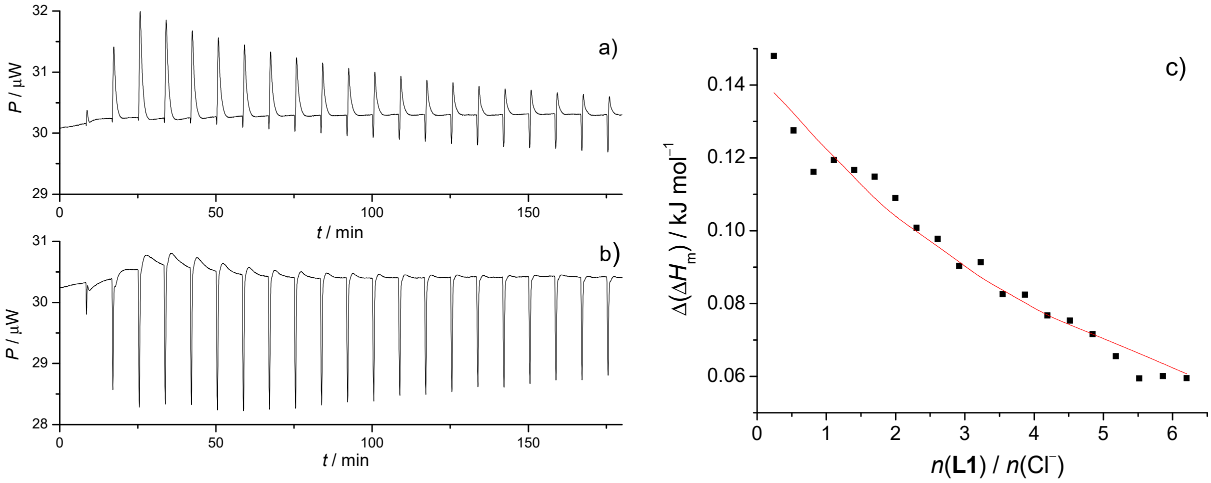

Figure S65. a) Microcalorimetric titration of TEACl (c = 2.63 × 10^{-3} mol dm^{-3}, V = 1.4295 cm^3) with **L1** (c = 0.07 mol dm^{-3}) in DMF; ϑ = 25 °C. b) Microcalorimetric titration of DMF with **L1** (c = 0.07 mol dm^{-3}); ϑ = 25 °C. c) Dependence of molar successive enthalpy change on n(**L1**) / $n(Cl^-)$ ratio. ■ experimental; — calculated.

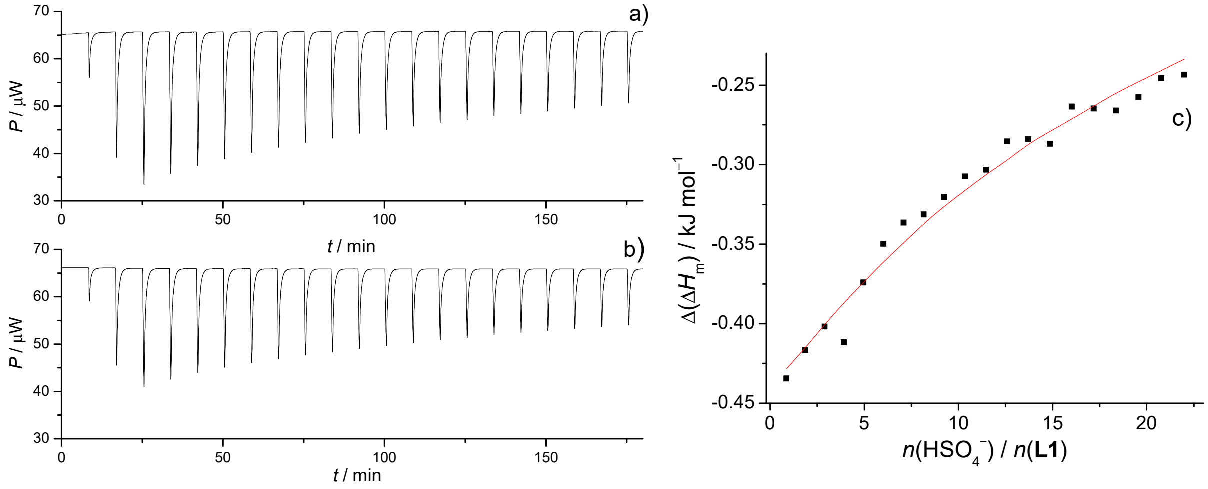

Figure S66. a) Microcalorimetric titration of **L1** ($c = 5.30 \times 10^{-4}$ mol dm^{-3}, $V = 1.4295$ cm^3) with TBAHSO4 (c = 0.0500 mol dm^{-3}) in DMF; ϑ = 25 °C. b) Microcalorimetric titration of DMF with TBAHSO4 ($c = 0.0500$ mol dm^{-3}); $\vartheta = 25$ °C. c) Dependence of molar successive enthalpy change on $n(HSO_4^-)$ / n(**L1**) ratio. ■ experimental; — calculated.

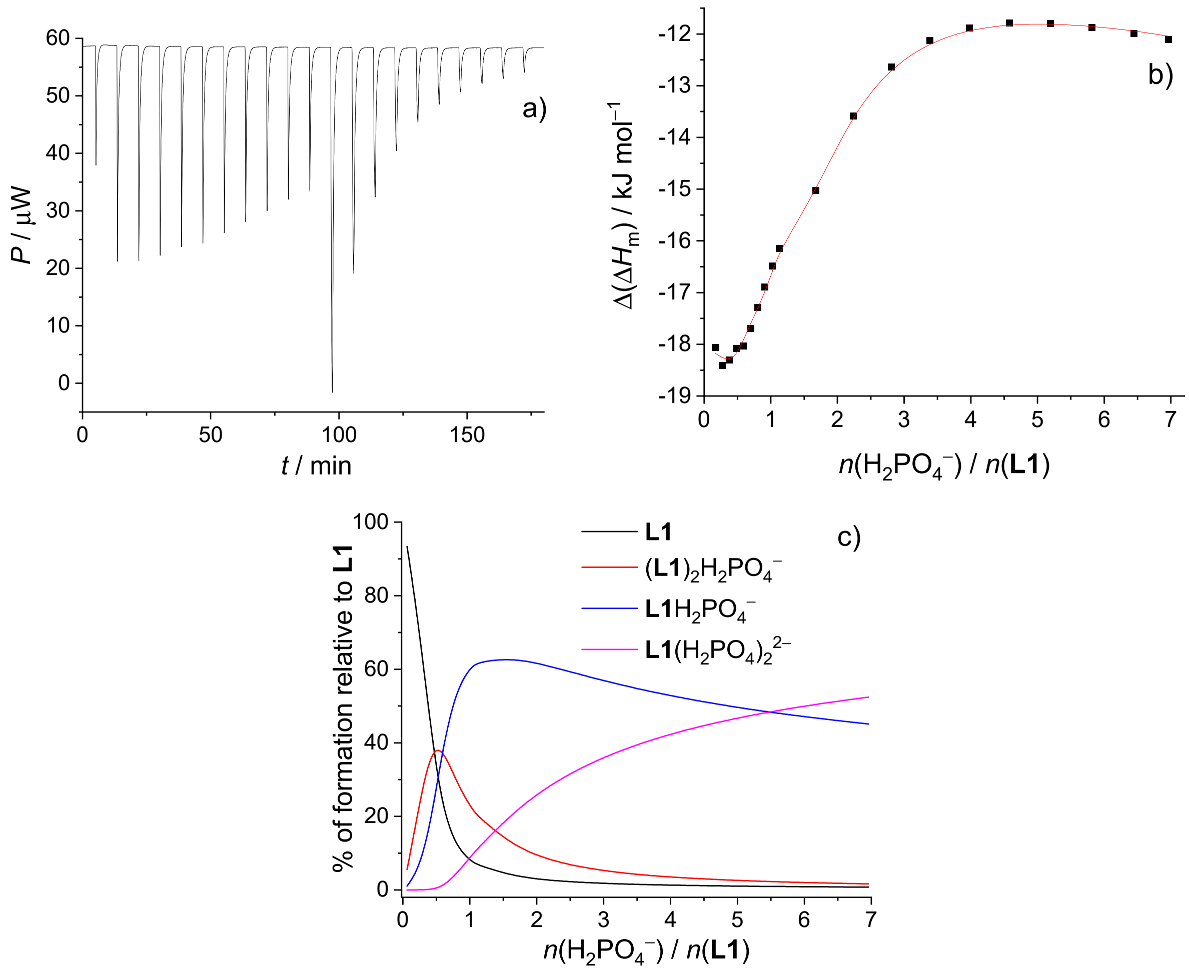

Figure S67. a) Microcalorimetric titration of **L1** ($c = 1.00 \times 10^{-3}$ mol dm^{-3}, $V = 1.4295$ cm^3) with $TBAH_2PO_4$ (c = 0.0299 mol dm^{-3}) in DMF; ϑ = 25 °C. b) Dependence of molar successive enthalpy change on $n(H_2PO_4^-)$ / n(**L1**) ratio. ■ experimental; — calculated. c) Distribution of complex species during the titration of peptide **L1** with $TBAH_2PO_4$.

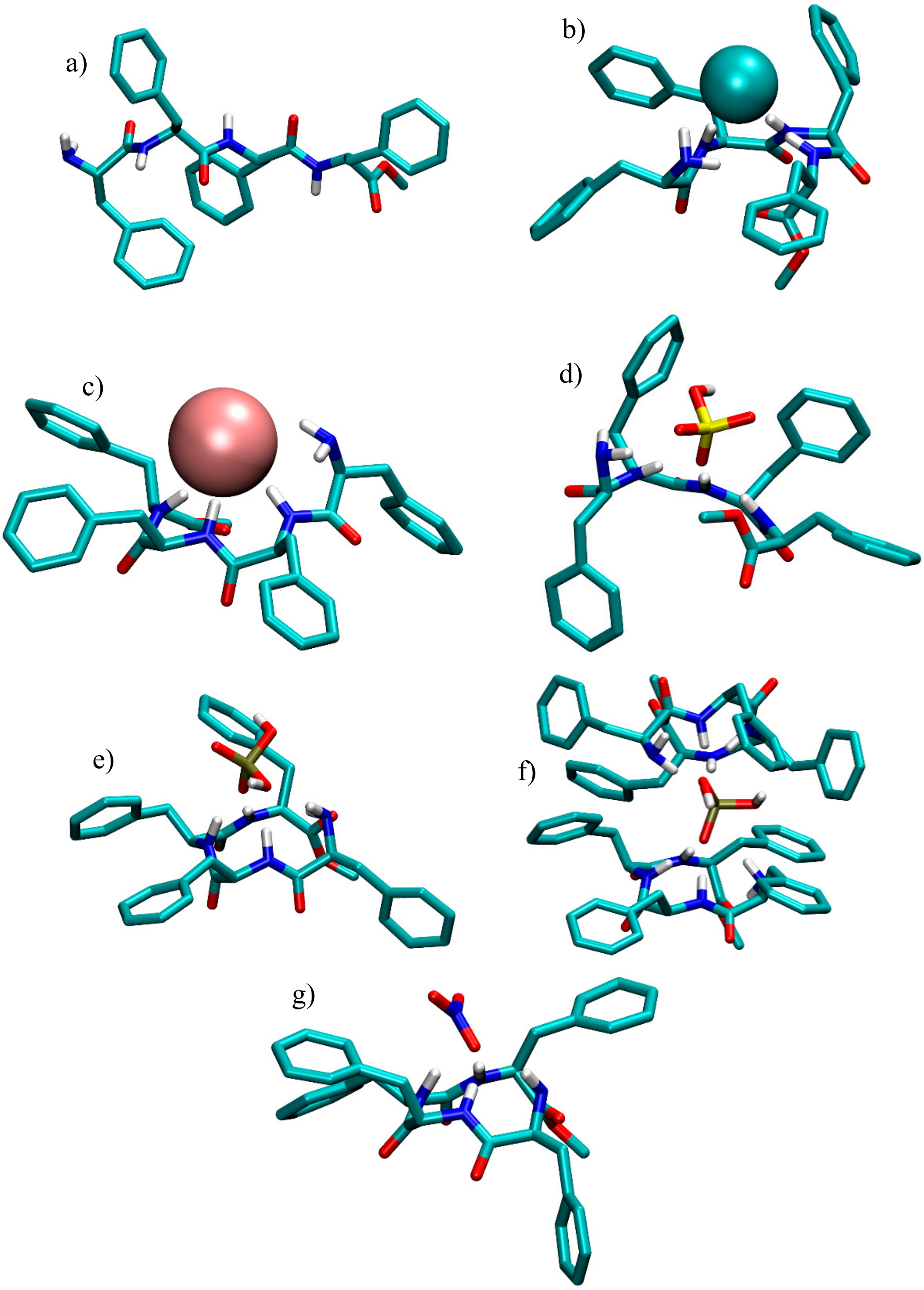

Figure S68. Representative structures of a) free peptide **L1** and its complexes with b) Cl^-, c) Br^-, d) HSO_4^-, e) $H_2PO_4^-$, f) sandwich complex with $H_2PO_4^-$, and g) NO_3^- obtained by MD simulations in DMF. Peptide hydrogen atoms bound to carbon atoms are omitted for clarity.

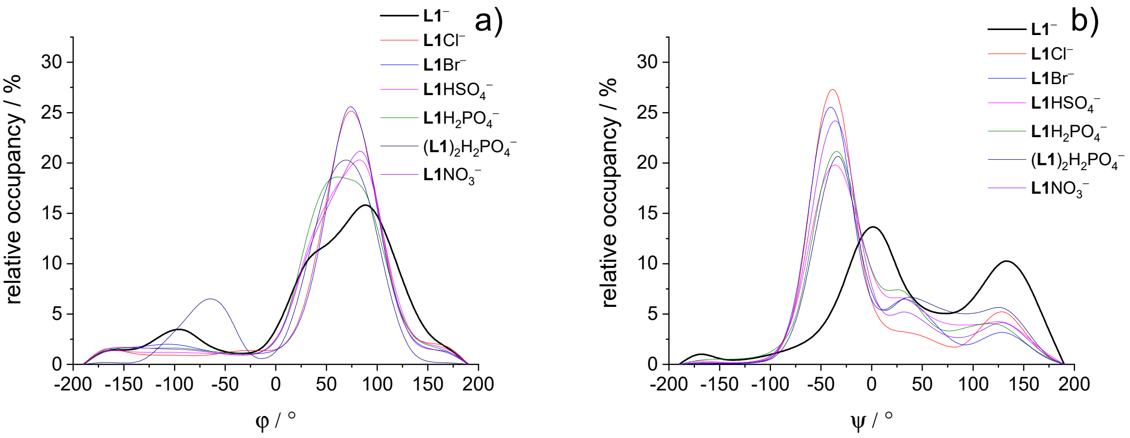

Figure S69. Distribution of a) φ, b) ψ angles of free peptide **L1** and its complexes with anions obtained by MD simulation in DMF at 25 °C.

Table S7. Energies of interactions between different species (**L1**, A$^-$, and DMF) obtained by MD simulations in DMF at 25 °C.

	free	Cl$^-$	Br$^-$	HSO_4^-	$H_2PO_4^-$		NO_3^-
					(**L1**)$_2H_2PO_4^-$	**L1**$H_2PO_4^-$	
E(**L1**–A$^-$) / kJ mol^{-1}	–	–198	–187	–183	–394	–228	–211
E(**L1**–DMF) / kJ mol^{-1}	–471	–382	–378	–379	–694	–381	–384
E(A$^-$–DMF) / kJ mol^{-1}	–	–63	–61	–92	–30	–89	–85

Table S8. Distances between nitrogen atom of N terminus and carbon atom of C terminus on free peptide **L1** and its complexes with anions obtained by MD simulations in DMF at 25 °C.

	free	Cl$^-$	Br$^-$	HSO_4^-	$H_2PO_4^-$	NO_3^-
d(C–N) / Å	8.6 ± 2.1	4.7 ± 0.9	5.3 ± 0.9	5.6 ± 1.0	5.3 ± 0.9	5.2 ± 1.1

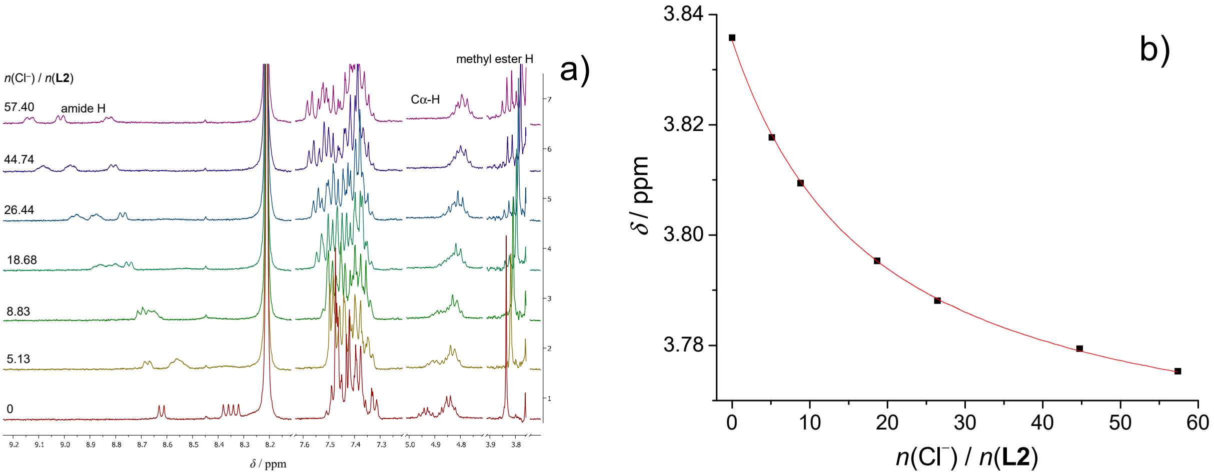

Figure S70. a) ^{1}H NMR titration of **L2** ($c = 5.21 \times 10^{-4}$ mol dm^{-3}) with TEACl ($c = 8.13 \times 10^{-4}$ mol dm^{-3}) in deuterated DMF at $\vartheta = 25$ °C; V_0 (**L2**) = 0.50 cm^3. b) The dependence of chemical shift of **L2** methyl ester protons at 3.84 ppm on $n(\mathrm{Cl}^-)$ / n(**L2**) molar ratio.

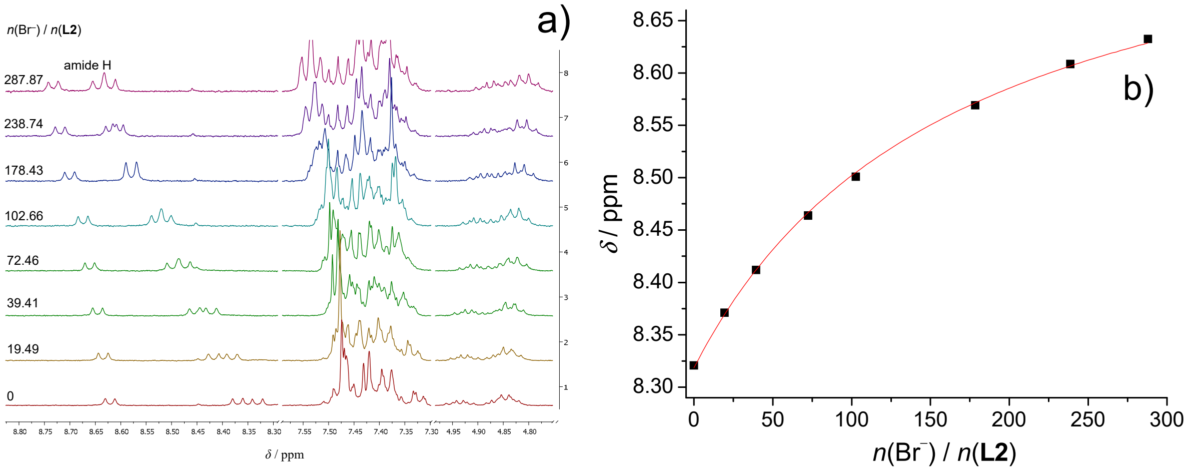

Figure S71. a) ^{1}H NMR titration of **L2** ($c = 5.21 \times 10^{-4}$ mol dm^{-3}) with TBABr ($c = 0.401$ mol dm^{-3}) in deuterated DMF at $\vartheta = 25$ °C; V_0 (**L2**) = 0.50 cm^3. b) The dependence of chemical shift of **L2** amide protons at 8.30 ppm on $n(\mathrm{Br}^-)$ / n(**L2**) molar ratio.

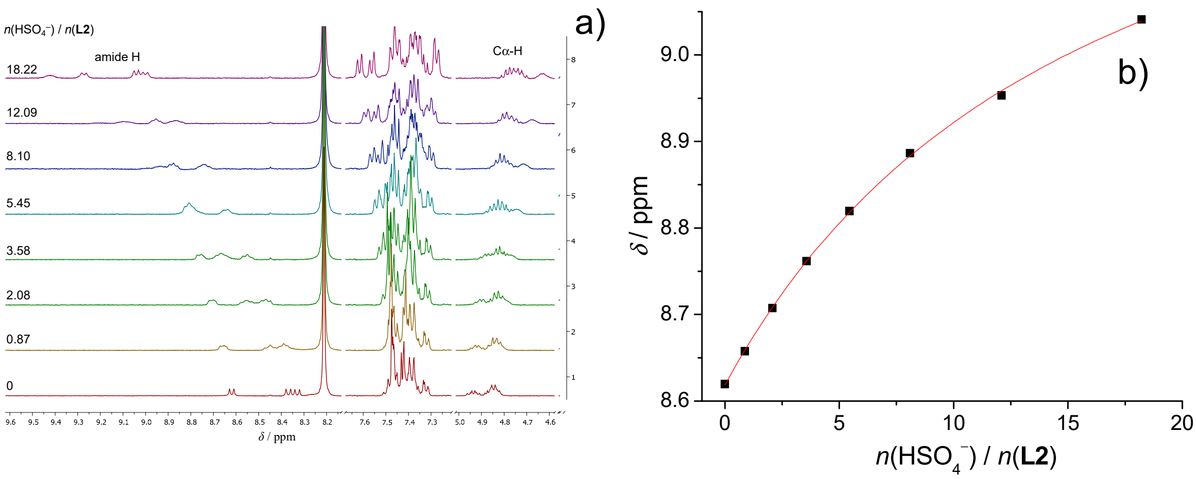

Figure S72. a) ^{1}H NMR titration of **L2** (c = 1.05 × 10^{-3} mol dm^{-3}) with TBAHSO$_4$ (c = 0.0516 mol dm^{-3}) in deuterated DMF at ϑ = 25 °C; V_0 (**L2**) = 0.50 cm^3. b) The dependence of chemical shift of **L2** amide protons at 8.60 ppm on $n(HSO_4^-)$ / n(**L2**) molar ratio.

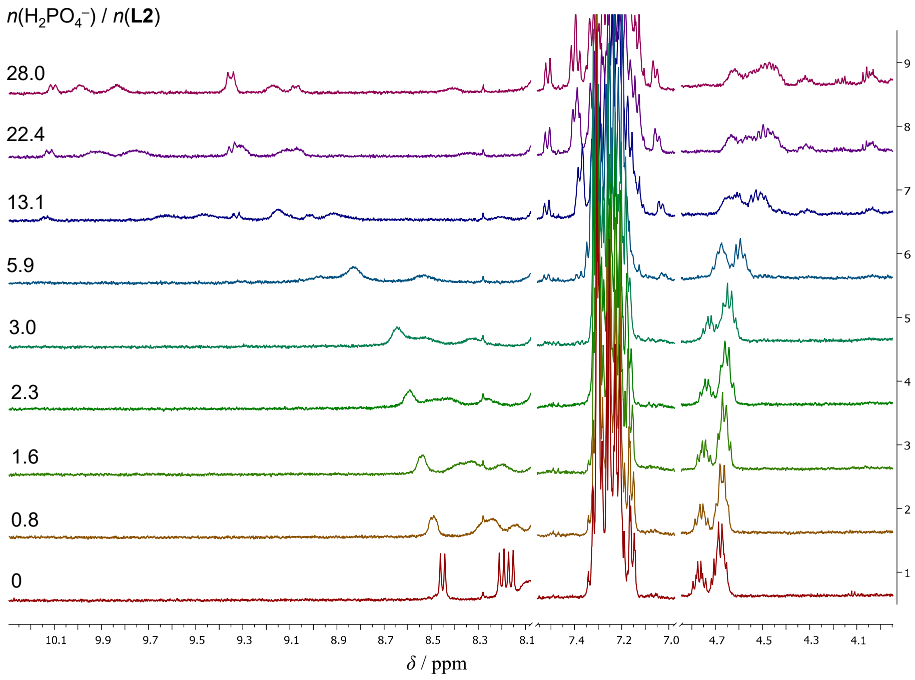

Figure S73. ^{1}H NMR titration of **L2** (c = 1.05 × 10^{-3} mol dm^{-3}) with TBAH$_2$PO$_4$ (c = 0.0795 mol dm^{-3}) in deuterated DMF at ϑ = 25 °C; V_0 (**L2**) = 0.50 cm^3.

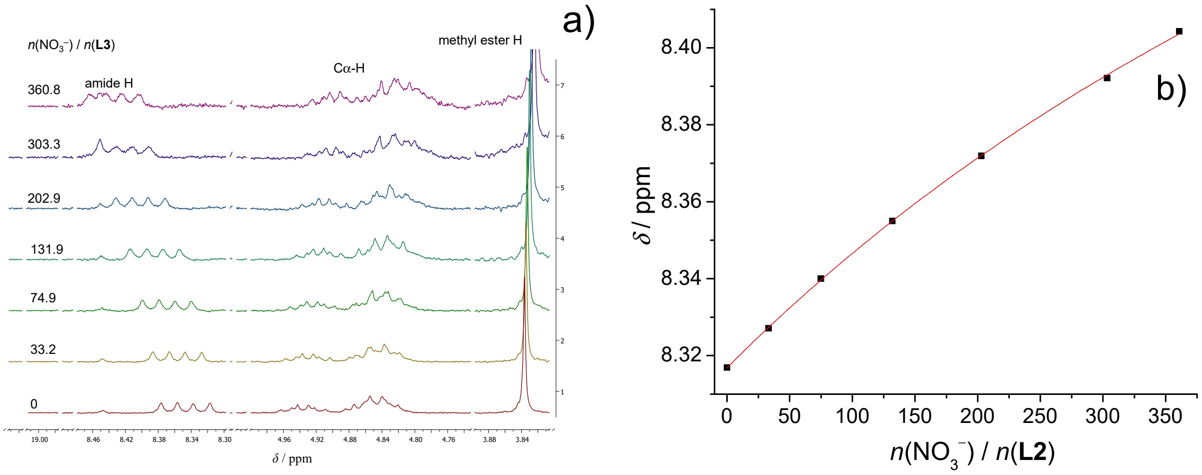

Figure S74. a) ^{1}H NMR titration of **L2** (c = 4.98 × 10^{-4} mol dm^{-3}) with TBANO$_3$ (c = 0.452 mol dm^{-3}) in deuterated DMF; ϑ = 25 °C at V_0 (**L2**) = 0.50 cm^3. b) The dependence of chemical shift of **L2** amide protons at 8.32 ppm on $n(NO_3^-)$ / n(**L2**) molar ratio.

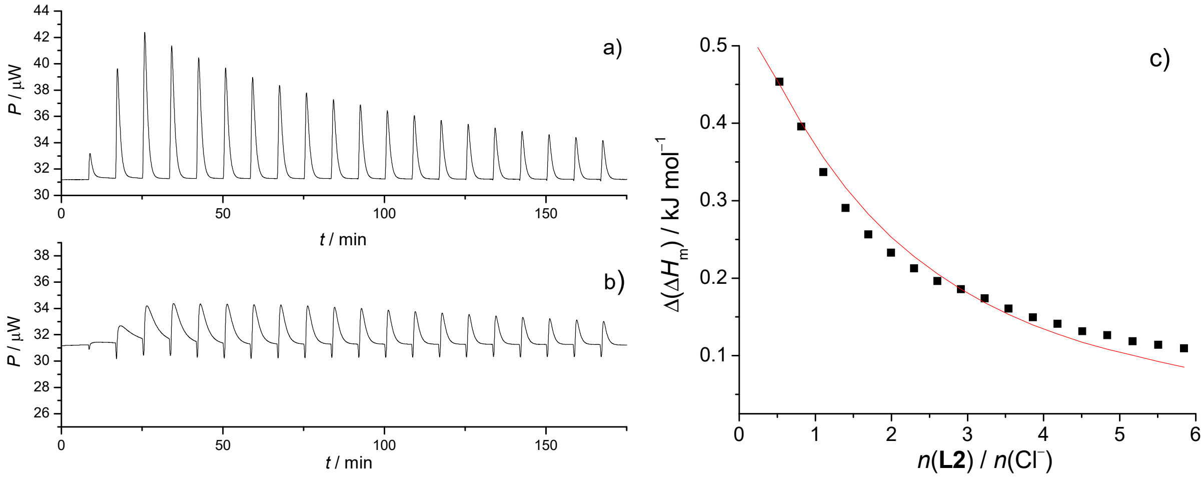

Figure S75. a) Microcalorimetric titration of TEACl (c = 2.63 × 10^{-3} mol dm^{-3}, V = 1.4331 cm^3) with **L2** (c = 0.0701 mol dm^{-3}) in DMF; ϑ = 25 °C. b) Microcalorimetric titration of DMF with **L2** (c = 0.0701 mol dm^{-3}); ϑ = 25 °C. c) Dependence of molar successive enthalpy change on n(**L2**) / $n(Cl^-)$ ratio. ■ experimental; — calculated.

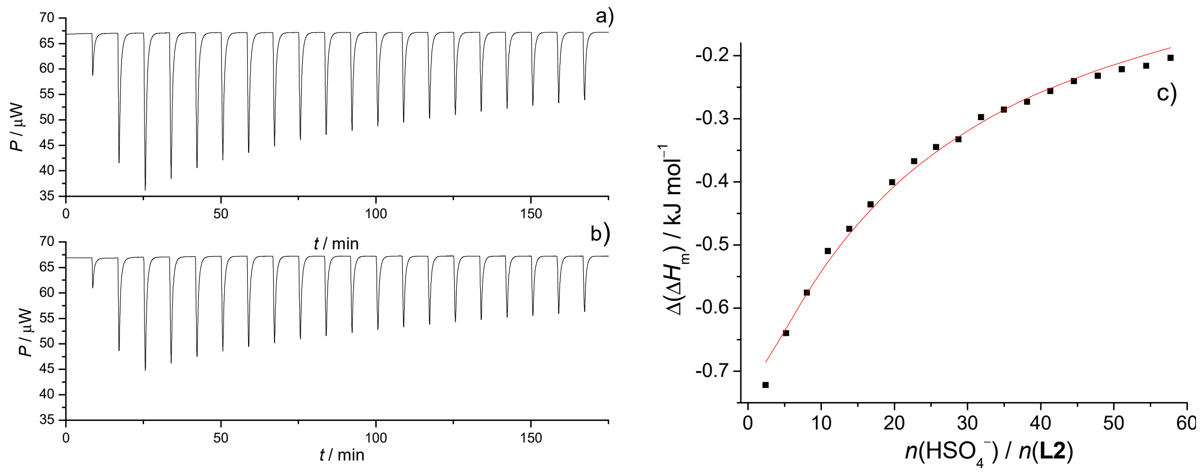

Figure S76. a) Microcalorimetric titration of **L2** (c = 1.90 × 10^{-4} mol dm^{-3}, V = 1.4331 cm^3) with $TBAHSO_4$ (c = 0.0500 mol dm^{-3}) in DMF; ϑ = 25 °C. b) Microcalorimetric titration of DMF with $TBAHSO_4$ (c = 0.0500 mol dm^{-3}); ϑ = 25 °C. c) Dependence of molar successive enthalpy change on $n(HSO_4^-)$ / n(**L2**) ratio. ■ experimental; — calculated.

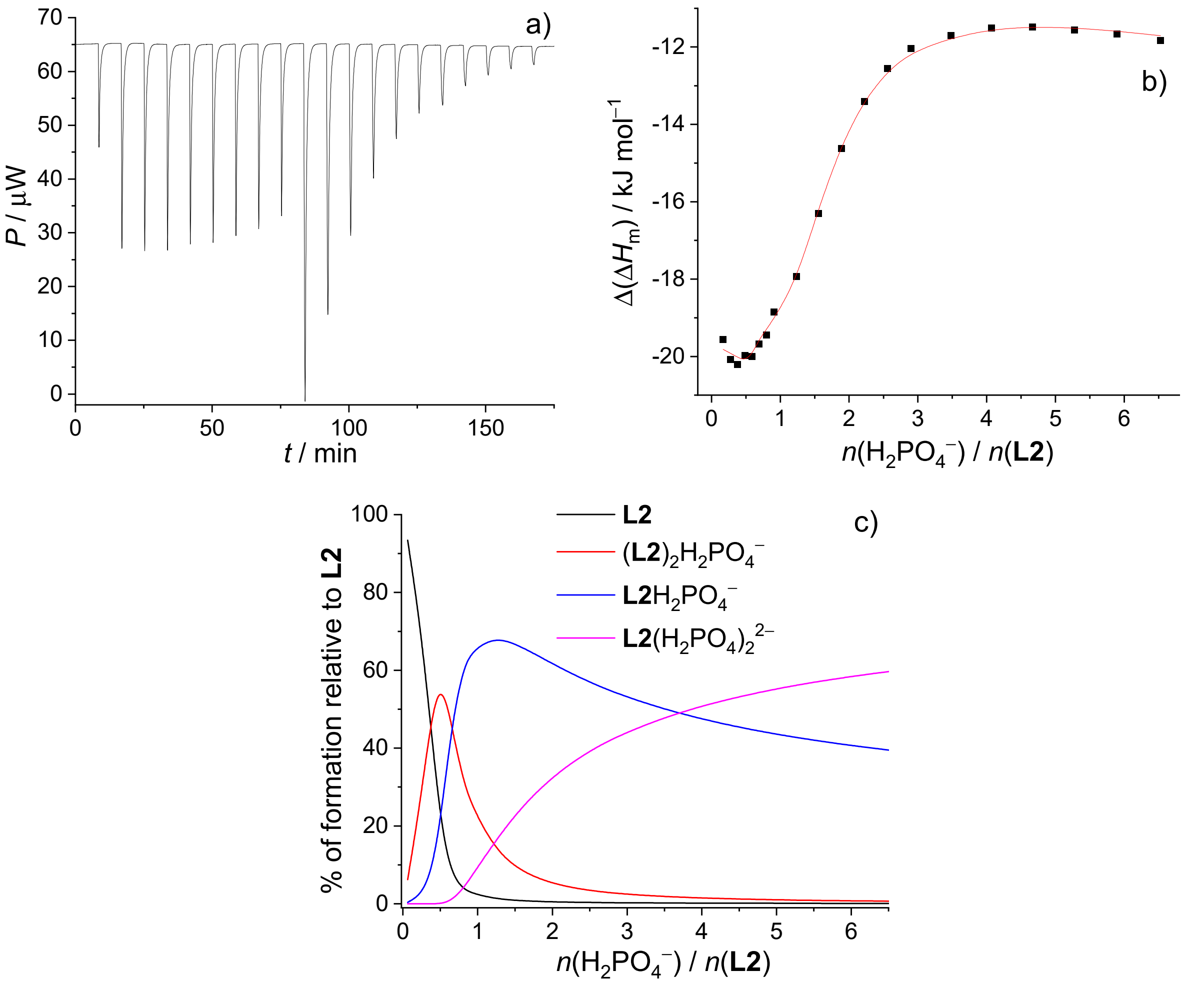

Figure S77. a) Microcalorimetric titration of **L2** ($c = 1.02 \times 10^{-3}$ mol dm^{-3}, $V = 1.4331$ cm^3) with $TBAH_2PO_4$ ($c = 0.0302$ mol dm^{-3}) in DMF; $\vartheta = 25$ °C. b) Dependence of molar successive enthalpy change on $n(H_2PO_4^-)$ / n(**L2**) ratio. ■ experimental; — calculated. c) Distribution of complex species during the titration of peptide **L2** with $TBAH_2PO_4$.

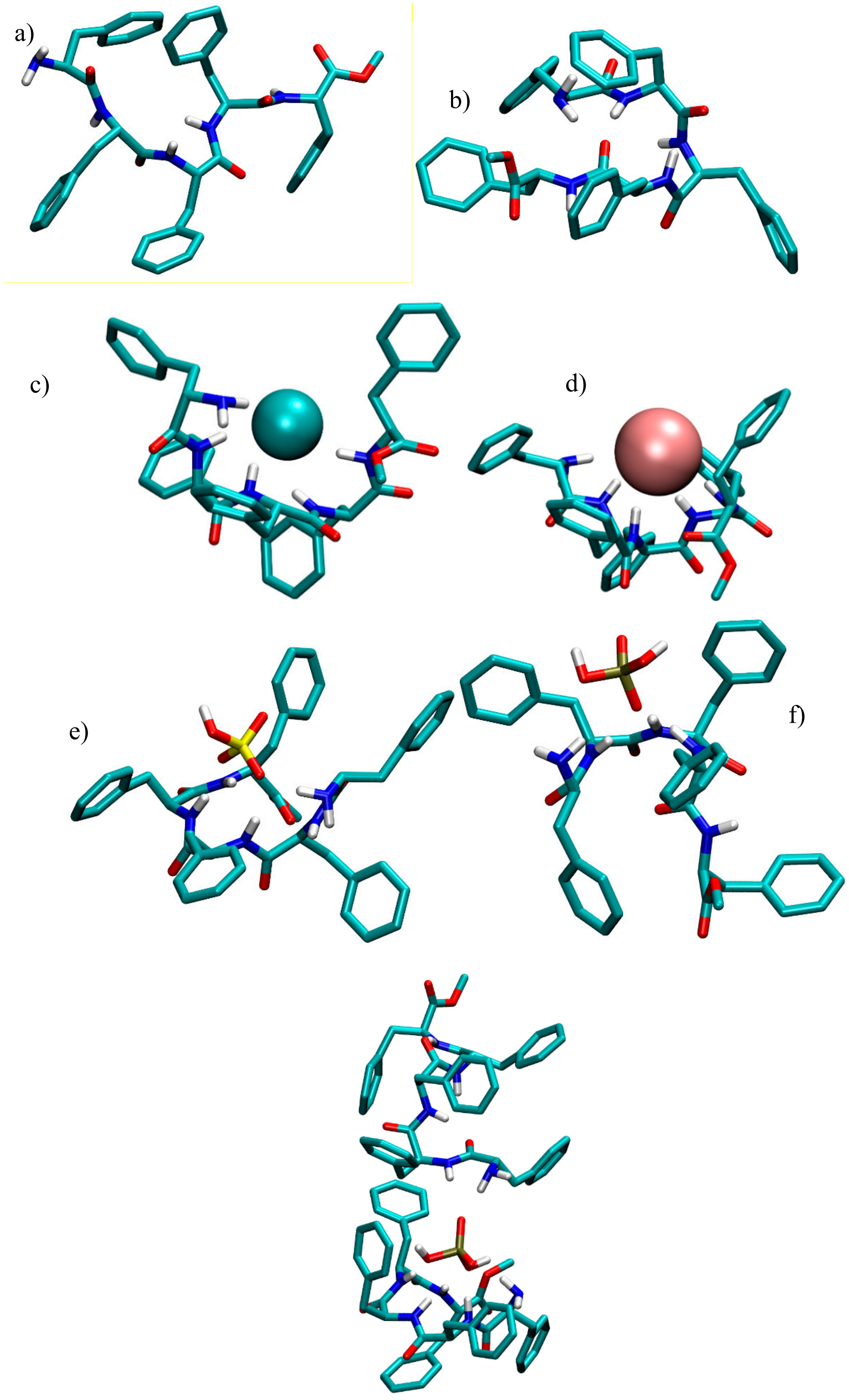
a)
b)
c)
d)
e)
f)

g)

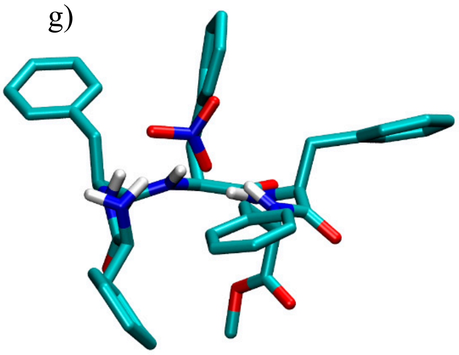

Figure S78. Representative structures of a) free peptide **L2** and its complexes with b) Cl^-, c) Br^-, d) HSO_4^-, e) $H_2PO_4^-$, f) sandwich complex with $H_2PO_4^-$, and g) NO_3^- obtained by MD simulations in DMF. Peptide hydrogen atoms bound to carbon atoms are omitted for clarity.

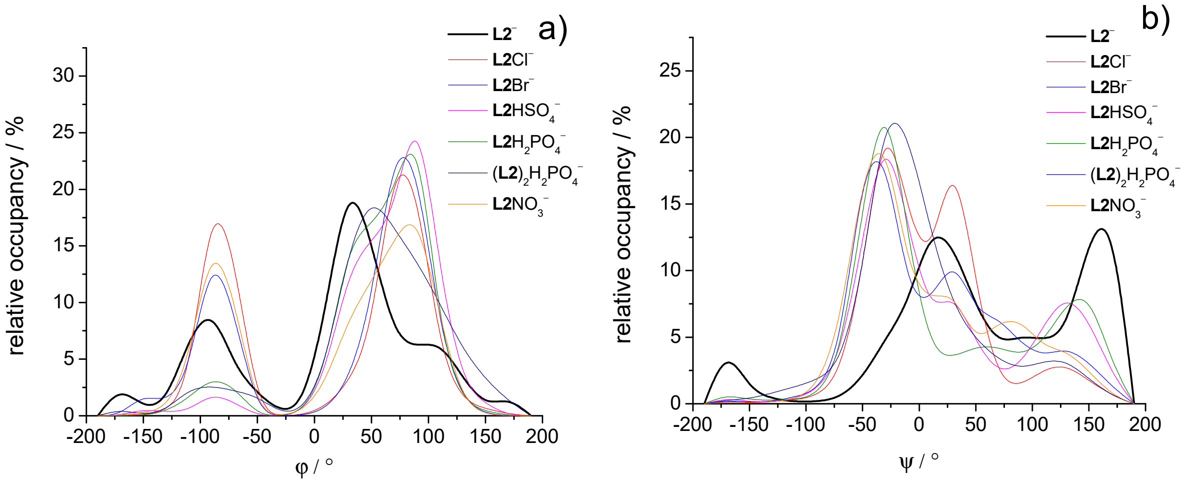

Figure S79. Distribution of a) φ, b) ψ angles of free peptide **L2** and its complexes with anions obtained by MD simulation in DMF at 25 °C.

Table S9. Energies of interactions between different species (**L2**, A^-, and DMF) obtained by MD simulations in DMF at 25 °C.

	free	Cl^-	Br^-	HSO_4^-	$H_2PO_4^-$		NO_3^-
					(**L2**)$_2H_2PO_4^-$	**L2**$H_2PO_4^-$	
E(**L2**–A^-) / kJ mol^{-1}	–	–237	–220	–181	–359	–232	–249
E(**L2**–DMF) / kJ mol^{-1}	–550	–470	–455	–487	–798	–470	–454
E(A^-–DMF) / kJ mol^{-1}	–	–50	–50	–71	–30	–88	–70

Table S10. Distances between nitrogen atom of N terminus and carbon atom of C terminus on free peptide **L2** and its complexes with anions obtained by MD simulations in DMF at 25 °C.

	free	Cl^-	Br^-	HSO_4^-	$H_2PO_4^-$	NO_3^-
d(C–N) / Å	9.9 ± 2.9	7.9 ± 1.0	7.7 ± 1.0	8.6 ± 1.3	6.6 ± 1.5	7.1 ± 1.6

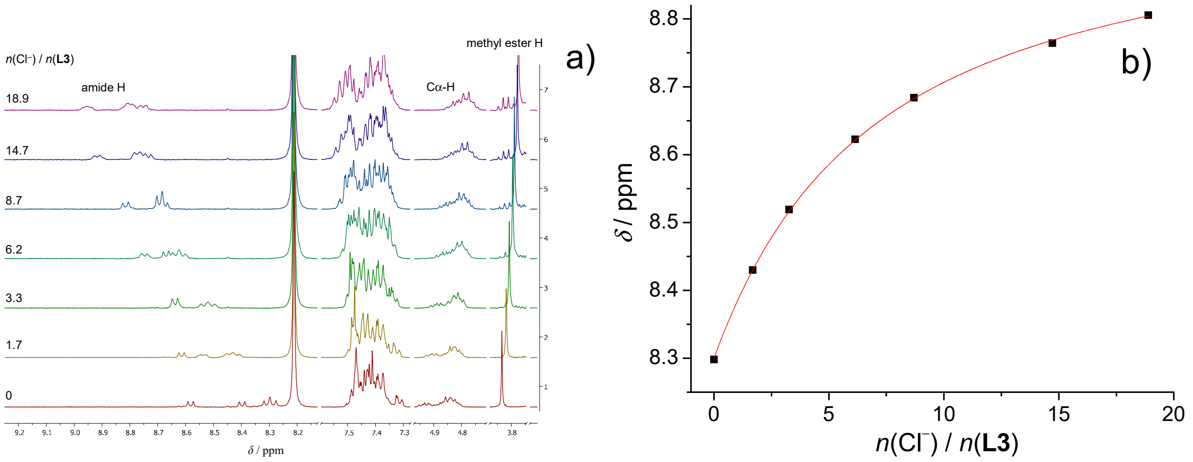

Figure S80. a) ^{1}H NMR titration of **L3** (c = 1.00 × 10^{-3} mol dm^{-3}) with TEACl (c = 0.515 mol dm^{-3}) in deuterated DMF at ϑ = 25 °C; V_0 (**L3**) = 0.50 cm^3. b) The dependence of chemical shift of **L3** amide protons at 8.30 ppm on $n(Cl^-)$ / n(**L3**) molar ratio.

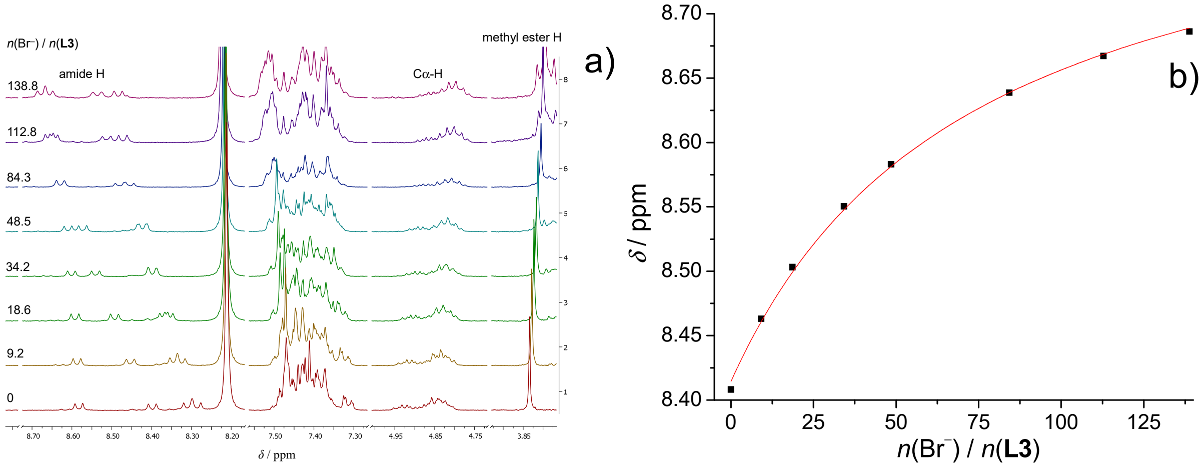

Figure S81. a) ^{1}H NMR titration of **L3** (c = 1.00 × 10^{-3} mol dm^{-3}) with TBABr (c = 0.365 mol dm^{-3}) in deuterated DMF at ϑ = 25 °C; V_0 (**L3**) = 0.50 cm^3. b) The dependence of chemical shift of **L3** amide protons at 8.40 ppm on $n(Br^-)$ / n(**L3**) molar ratio.

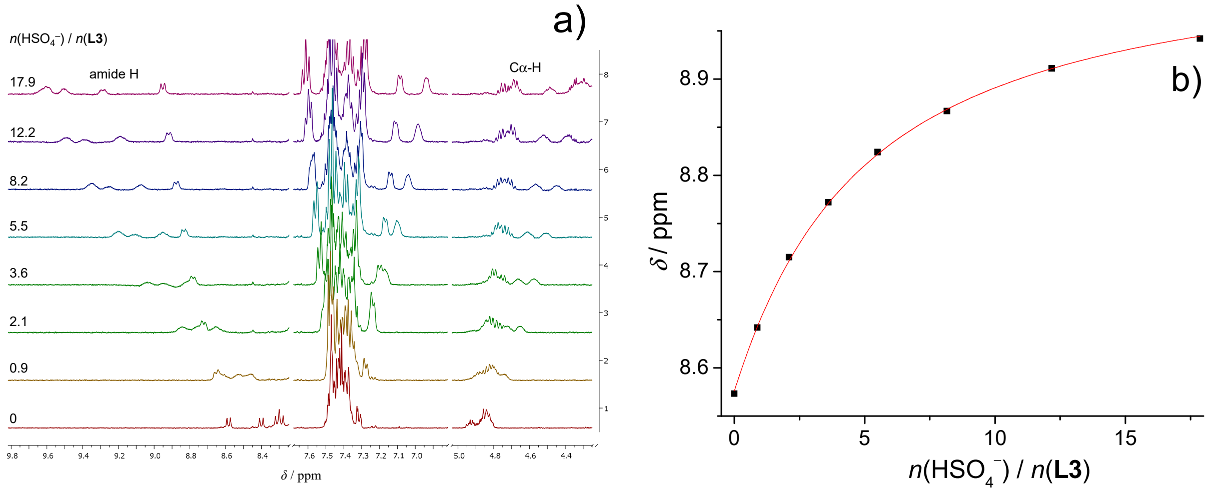

Figure S82. a) ^{1}H NMR titration of **L3** (c = 9.77 × 10^{-4} mol dm^{-3}) with TBAHSO$_4$ (c = 0.0486 mol dm^{-3}) in deuterated DMF at ϑ = 25 °C; V_0 (**L3**) = 0.50 cm^3. b) The dependence of chemical shift of **L3** amide protons at 8.55 ppm on $n(HSO_4^{-})$ / n(**L3**) molar ratio.

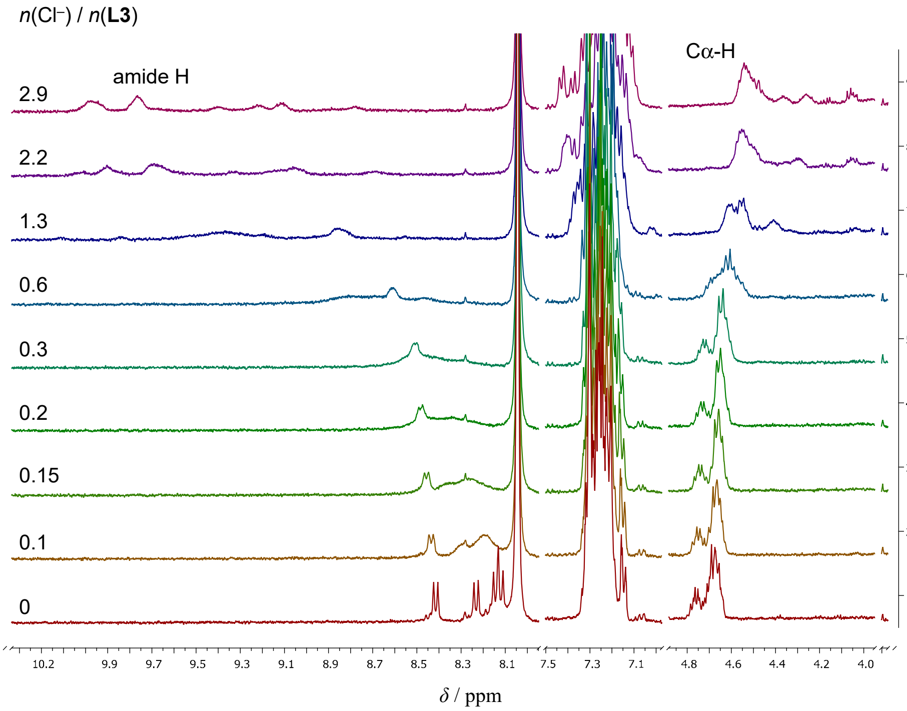

Figure S83. ^{1}H NMR titration of **L3** (c = 9.77 × 10^{-4} mol dm^{-3}) with TBAH$_2$PO$_4$ (c = 7.60 × 10^{-3} mol/dm^3) in deuterated DMF at ϑ = 25 °C; V_0 (**L3**) = 0.50 cm^3.

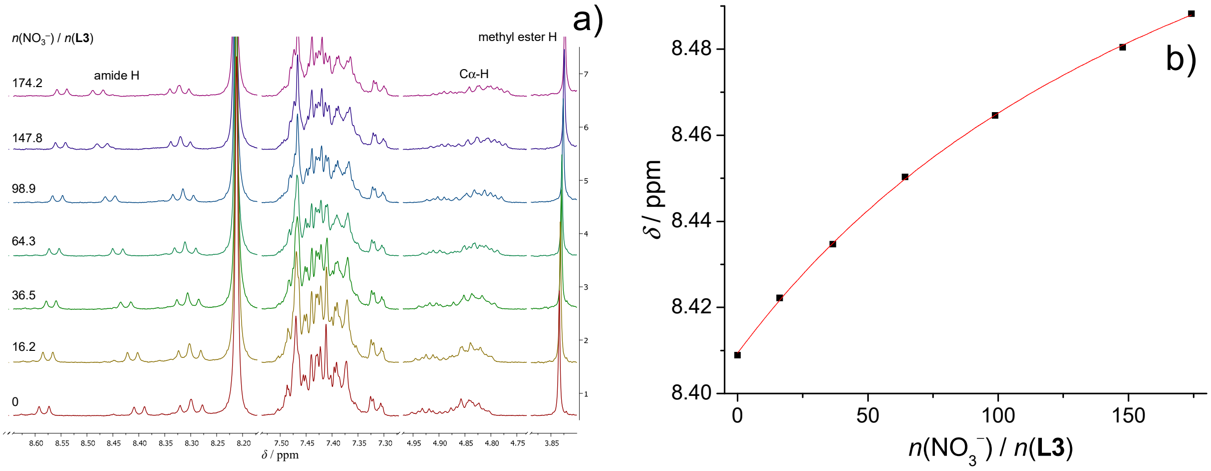

Figure S84. a) ^{1}H NMR titration of **L3** ($c = 9.96 \times 10^{-4}$ mol dm^{-3}) with $TBANO_3$ ($c = 0.441$ mol dm^{-3}) in deuterated DMF at $\vartheta = 25$ °C; V_0 (**L3**) = 0.50 cm^3. b) The dependence of chemical shift of **L3** amide protons at 8.41 ppm on $n(NO_3^-)$ / n(**L3**) molar ratio.

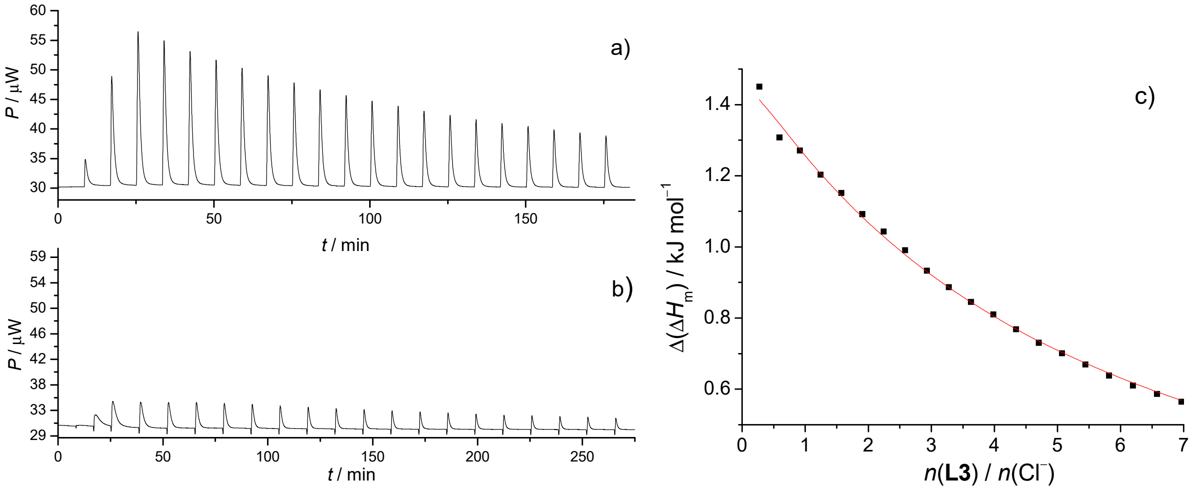

Figure S85. a) Microcalorimetric titration of TEACl ($c = 2.01 \times 10^{-3}$ mol dm^{-3}, $V = 1.4295$ cm^3) with **L3** ($c = 0.0601$ mol dm^{-3}) in DMF; $\vartheta = 25$ °C. b) Microcalorimetric titration of DMF with **L3** ($c = 0.0601$ mol dm^{-3}); $\vartheta = 25$ °C. c) Dependence of molar successive enthalpy change on n(**L3**) / n(Cl$^-$) ratio. ■ experimental; — calculated.

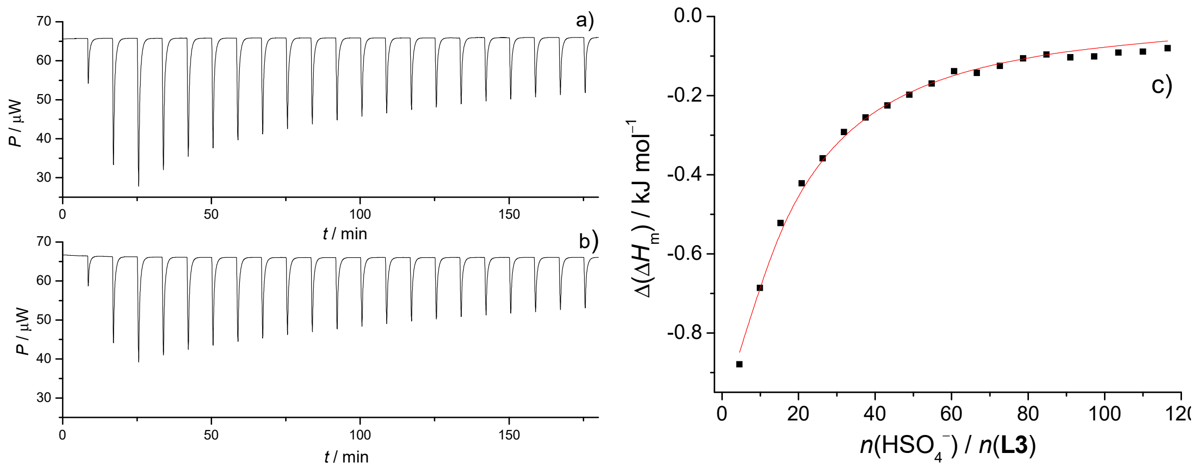

Figure S86. a) Microcalorimetric titration of **L3** ($c = 1.00 \times 10^{-4}$ mol dm^{-3}, $V = 1.4295$ cm^{3}) with TBAHSO4 (c = 0.0500 mol dm^{-3}) in DMF; ϑ = 25 °C. b) Microcalorimetric titration of DMF with TBAHSO4 ($c = 0.0500$ mol dm^{-3}); $\vartheta = 25$ °C. c) Dependence of molar successive enthalpy change on $n(HSO_4^-)$ / n(**L3**) ratio. ■ experimental; — calculated.

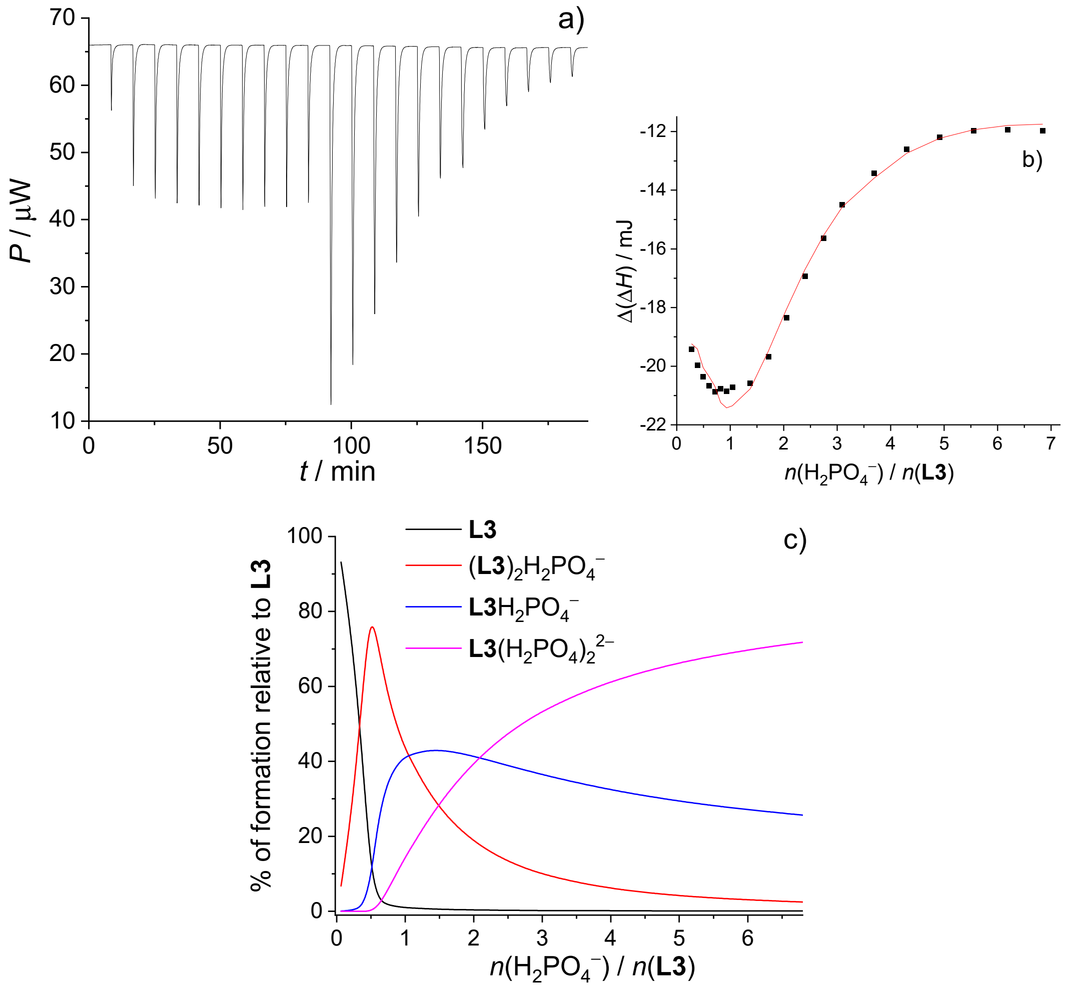

Figure S87. a) Microcalorimetric titration of **L3** ($c = 5.02 \times 10^{-4}$ mol dm^{-3}, $V = 1.4295$ cm^3) with $TBAH_2PO_4$ (c = 0.0153 mol dm^{-3}) in DMF; ϑ = 25 °C. b) Dependence of molar successive enthalpy change on $n(H_2PO_4^-)$ / n(**L3**) ratio. ■ experimental; — calculated. c) Distribution of complex species during the titration of peptide **L3** with $TBAH_2PO_4$.

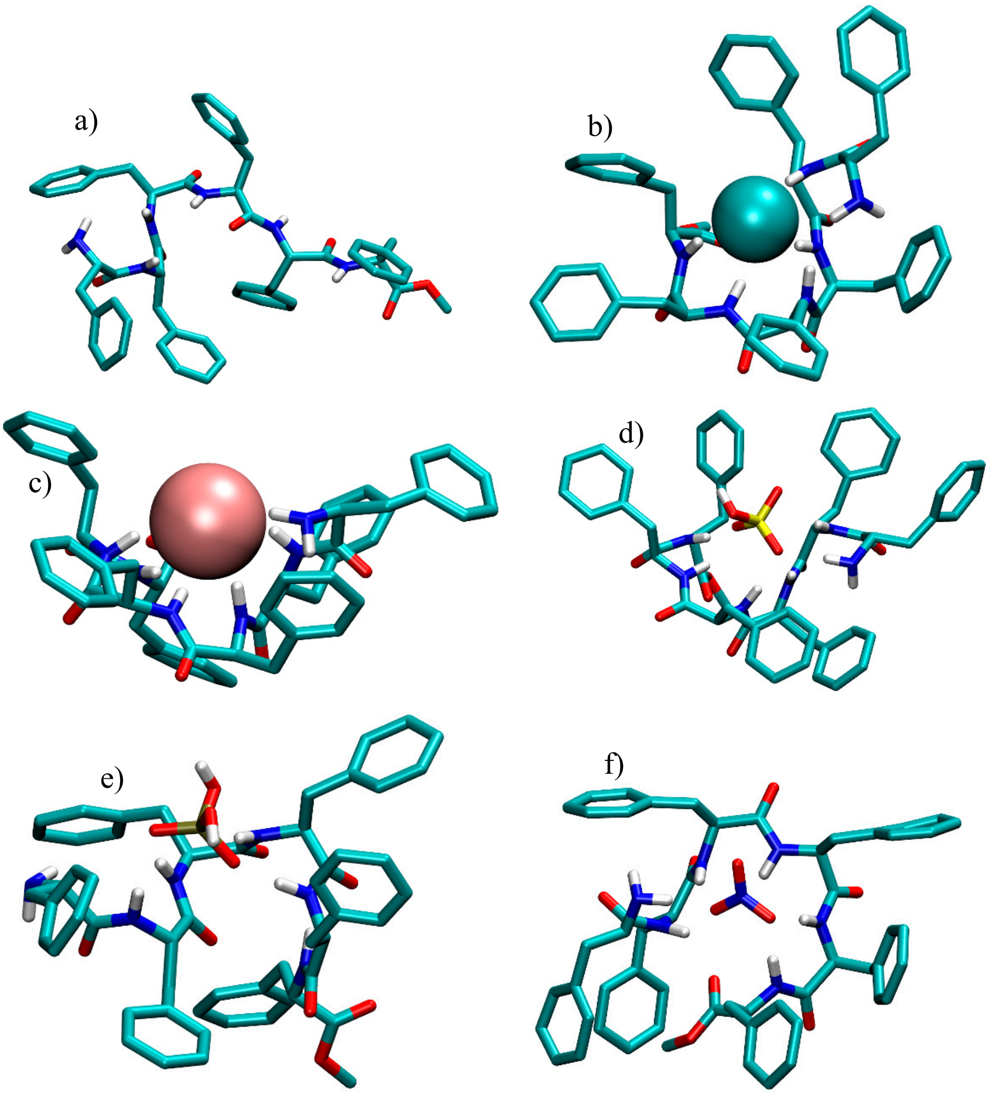

Figure S88. Representative structures of a) free peptide **L3** and its complexes with b) Cl^{-}, c) Br^{-}, d) HSO_4^{-}, e) $H_2PO_4^{-}$, and f) NO_3^{-}obtained by MD simulations in DMF. Peptide hydrogen atoms bound to carbon atoms are omitted for clarity.

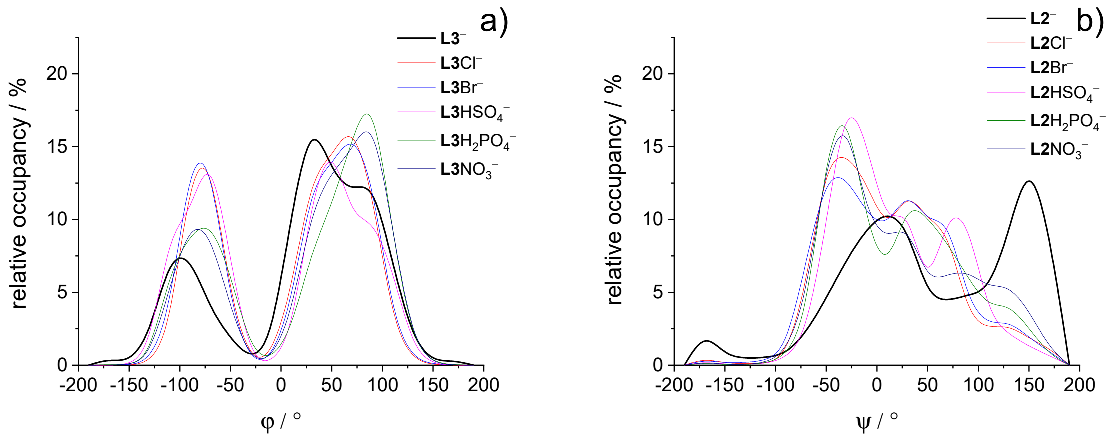

Figure S89. Distribution of a) φ, b) ψ angles of free peptide **L3** and its complexes with anions obtained by MD simulation in DMF at 25 °C.

Table S11. Energies of interactions between different species (**L3**, A^-, and DMF) obtained by MD simulations in DMF at 25 °C.

	free	Cl^-	Br^-	HSO_4^-	$H_2PO_4^-$	NO_3^-
E(**L3**–A^-) / kJ mol^{-1}	–	–276	–252	–268	–300	–270
E(**L3**–DMF) / kJ mol^{-1}	–647	–513	–512	–483	–524	–529
E(A^-–DMF) / kJ mol^{-1}	–	–39	–42	–58	–65	63

Table S12. Distances between nitrogen atom of N terminus and carbon atom of C terminus on free peptide **L3** and its complexes with anions obtained by MD simulations in DMF at 25 °C.

	free	Cl^-	Br^-	HSO_4^-	$H_2PO_4^-$	NO_3^-
d(C–N) / Å	11.1 ± 3.4	7.8 ± 0.6	8.3 ± 1.0	8.4 ± 0.6	9.5 ± 1.2	8.9 ± 1.6
